# Unveiling the absorbed bioactive constituents of *Cuscuta* seeds: A systematic strategy integrating automated MS annotation, *in vivo* chemometric screening and bioactivity evaluation

**DOI:** 10.1016/j.fochx.2026.103564

**Published:** 2026-01-20

**Authors:** Xi-yang Tang, Ming-jia Ma, Meng-le Du, Lv-qi Xie, Jia-jia Chen, Ze-xi Tan, Zhi-jian Su, Zi-qin Dai, Lei Huang, Yi Dai

**Affiliations:** a State Key Laboratory of Bioactive Molecules and Druggability Assessment, Guangdong Basic Research Center of Excellence for Natural Bioactive Molecules and Discovery of Innovative Drugs, Institute of Traditional Chinese Medicine & Natural Products, College of Pharmacy, Guangdong Province Key Laboratory of Pharmacodynamic Constituents of TCM and New Drugs Research, and International Cooperative Laboratory of Traditional Chinese Medicine Modernization and Innovative Drug Development of Ministry of Education (MOE) of China, Jinan University, Guangzhou 510632, PR China; b Department of Pharmacy, First People's Hospital of Yancheng, Yancheng 224006, PR China; c Department of Food Science and Engineering, Department of Developmental and Regenerative Biology, Biopharmaceutical R&D Center, Jinan University, Guangzhou 510632, PR China; d Guangzhou Huibiao Testing Technology Center, Guangzhou 510632, PR China

**Keywords:** *Cuscuta* seed (CS), MATLAB automated MS annotation, *In vivo* chemometric screening, Absorbed bioactive constituents, H_2_O_2_-induced R2C leydig cell, Progesterone

## Abstract

The strategy integrated MATLAB automated MS annotation, *in vivo* chemometric screening, and progesterone assessment under oxidative stress was established for the identification of absorbed bioactive constituents of *Cuscuta* seed (CS). MATLAB platform characterized 203 components (flavonols, alkaloids, phenolic acids, etc) in CS extract, outperforming commercial software through its combinatorial “parent molecules + group fragments” database and automated neutral loss/diagnostic ion matching. This approach was designed to effectively minimize in-source fragmentation false positives, while its dual-dimension similarity algorithm refined molecular networking. Furthermore, 20 prototypes and 46 metabolites were discovered and identified in plasma and urine after oral administration of CS by OPLS-DA and MATLAB analysis platform. Cuscutamine and *p*-coumaric acid exhibited high systemic exposure. Based on *in vivo* metabolic analysis, 9 major absorbed constituents were revealed. Hyperoside, ferulic acid, cuscutamine, kaempferol, and quercetin demonstrated significant bioactivity by attenuating H₂O₂-induced oxidative damage in R2C Leydig cells and restoring progesterone levels.

## Highlights

• Novel MATLAB platform identified 203 compounds in *Cuscuta* seed extract.
• Dual dimensional similarity algorithm enhanced molecular network visualization for components.
• 20 prototypes and 46 metabolites were identified *in vivo*, 9 major constituents were revealed.
• Hyperoside, ferulic acid, cuscutamine, kaempferol, quercetin improved progesterone level under oxidative stress.

## 1 Introduction

*Cuscuta* seeds (CS), an edible resource in Asian countries, have garnered growing interest as a functional food due to their abundant bioactive compounds, including flavonols and phenolic acid (Changli et al., 2023; Ju et al., 2021; Liang et al., 2024; Ye et al., 2005; Yen et al., 2008; Zhang et al., 2025). Currently, CS was considered to regulate the body's endocrine system, control hormone levels, and mitigate the damage caused by endocrine disruption to the male or female reproductive organs or the reproductive system, serving to protect the reproductive system (Changli et al., 2023; Liang et al., 2024; K. Sun et al., 2012). Flavonols from CS could reverse this effect of H_2_O_2_-induced oxidative damage in human ovarian granulosa cells by promoting AMPK pathways (Ban & Li, 2023). The ethanol extract of CS exhibited significant antioxidant activity by increasing the levels of superoxide dismutase (SOD), catalase (CAT) and glutathione peroxidase (GPx) and decreasing the levels of malondialdehyde (MDA) (Yen et al., 2007). Historically consumed in health drink (Ahmad et al., 2017) and dietary nutritional supplements (Hong Bui et al., 2025; Yen et al., 2008), CS is valued for its purported benefits in promoting hair growth (Ahmad et al., 2017), improving reproductive health (Shu et al., 2022), and mitigating oxidative stress (Ahmad et al., 2017). Therefore, flavonols and phenolic acids from CS have dominated studies in antioxidant for use as functional food sources.

Currently, chemical characterization of CS is largely dependent on liquid chromatography-mass spectrometry (LC-MS) technology in combination with data processing tools such as Unifi, GNPS, *etc.* (Wang et al., 2022; Ye et al., 2005). However, *in vivo* metabolic profiles after oral administration of CS are still poorly characterized. This step is crucial for linking dietary intake to biological activity, since absorbed components usually mediate physiological effects. General analytical tools have inherent limitations for analysis of CS component *in vitro* and *in vivo*. First, common software algorithms of annotation (Unifi and GNPS) are biased toward known compound databases. Novel compounds and unknown metabolites from CS are often overlooked during annotation. Second, analytical challenges arise from adduct ion interference and in-source fragmentation. These ions, particularly those from flavonols, phenolic acids, and their phase II metabolites, are often misidentified as precursor ions, resulting in compound misidentification. Third, MS similarity metrics (*e.g.*, cosine scores) emphasize diagnostic ion peak intensity patterns and are unable to account for neutral losses specific to CS components, resulting in omissions in the visualization of compound classification. To overcome these challenges, there is an urgent need to develop an automated analysis system to address the annotation of unknown compounds, adduct ion interference, in-source fragmentation, and molecular network visualization in analysis of CS component *in vitro* and *in vivo.*

As a steroid hormone essential for regulating reproductive function and maintaining endocrine homeostasis, and the precursor to testosterone, progesterone serves as a primary indicator of reproductive function (Wiltbank et al., 2014). Oxidative stress is a key factor contributing to hormonal imbalances in metabolic disorders (Al-Kufaishi & Al-Musawi, 2024; Al-Suhaimi et al., 2024). The R2C rat Leydig tumor cell line retains the expression of key steroid synthases such as StAR, CYP11A1, and 3β-HSD, and exhibits reproducible responsiveness to various exogenous compounds. Therefore, it is considered an important *in vitro* model for screening substances that may affect endocrine function (M. W. Li et al., 2017; J. X. Sun et al., 2013; Wen et al., 2018). Progesterone synthesis in R2C cells has been reported to be blocked under conditions of oxidative stress (R. X. Li et al., 2023; Wen et al., 2018). To investigate the protective role of CS components against this dysfunction, a H_2_O_2_-induced oxidative damage model of rat R2C cells was used to recapitulate the redox imbalance of hormonal dysregulation. A direct link between the *in vivo* components of CS and endocrine protection efficacy was established by evaluating the ability of the major absorbed component of CS to restore progesterone levels in damaged R2C cells.

**Fig. 1:**
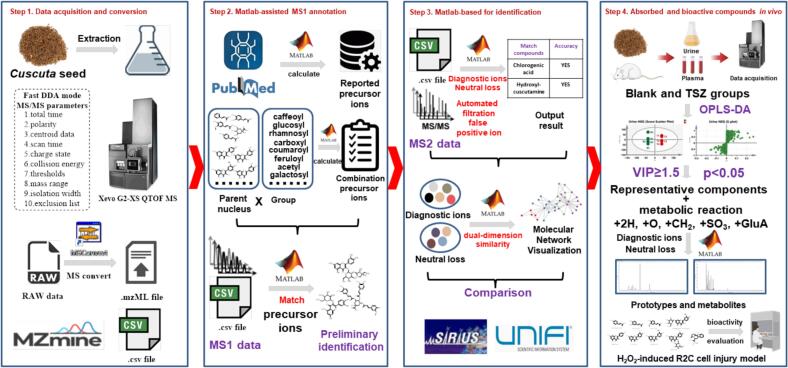
Flowchart for MATLAB automated MS annotation, *in vivo* chemometric screening, and progesterone assessment under oxidative stress to identify absorbed and bioactive compounds in *Cuscuta* seeds.

In this study, as shown in Fig. 1, MATLAB automated MS data analysis platform combined with *in vivo* chemometric analysis for intelligent assisted UPLC-Q-TOF/MS data processing and visualization was established to characterize chemical compounds in CS extract and absorbed compounds *in vivo*. Moreover, a rat leydig cell (R2C) injury model caused by hydrogen peroxide (H_2_O_2_) was used to evaluate the activities of major absorbed components and parent components of their metabolites on progesterone levels.

## 2 Material and methods

### 2.1 Chemical reagents and materials

*Cuscuta* seeds (*Cuscuta chinensis* Lam.) were obtained from Shaanxi, China. Authentic standards of raffinose, sucrose, neochlorogenic acid, chlorogenic acid, cryptochlorogenic acid, caffeic acid, *p*-coumaric acid, hyperoside, isoquercitrin, astragalin, isorhamnetin-3-β-O-glucoside, isorhamnetin-7-O-Glucoside, azelaic acid, isochlorogenic acid C, ferulic acid, cuscutamine, sesaminol diglucoside, kaempferol, isorhamnetin, and LPC (16:0) were purchased from Chengdu Push Bio-technology Co., Ltd. (Chengdu, China). Progesterone was obtained from Chengdu Alfa Biotechnology Co., Ltd. (Chengdu, China). Progesterone-d_9_ was purchased from Shanghai Zzbio Co., Ltd**.** (Shanghai, China). The purity of each compound was more than 98% determined by HPLC analysis. Distilled water (HPLC-grade) was purchased from Beijing Watson's Distilled Water (Beijing, China). Methanol and acetonitrile (LC-MS grade) were purchased from Fisher Scientific (Fair Lawn, New Jersey, USA). Formic acid (LC-MS grade) was purchased from Sigma-Aldrich (ST. Missouri, USA). Other reagents were analytical grade and purchased on the market. R2C was purchased from ATCC (Manassas, VA, USA).

### 2.2 Animal experiments

Male Sprague–Dawley rats (3–4 months, 220 ± 20 g) were obtained from the experimental animal center of Guangdong province (Guangzhou, China). The animal experiment protocol has been reviewed and approved by Laboratory Animal Ethics Committee of Jinan University (IACUC-20240318-03). Animals were housed in the specific pathogen free facility at the Jinan University Laboratory Animal Center and cared in accordance with the Public Health Services policy for the Guide for the Care and Use of Laboratory Animals. Rats were housed in environmentally controlled animal chambers (22–25 °C, 50–60% relative humidity, 12-h dark photoperiod) and were given water and fed normal food for two weeks prior to the experiment.

The rats were randomly divided into two groups, each consisting of five rats: CS group and blank group. The CS extracts were administered orally to rats (*n* = 5) at a dose of 1 g/kg. The blank group (n = 5) received the equivalent volume of water in the same way. Orbital venous blood was collected into heparinized tubes at 0.5 h, 1 h, 2 h, and 4 h under isoflurane anesthesia after oral administration of CS. All blood samples were then centrifuged at 14,000 rpm for 15 min at 4 °C and mixed to produce the pooled plasma. Urine samples were collected everyday along with the drug administration. The blank plasma/urine sample was taken by the same method from the rats in blank group. Furthermore, the rats were euthanized by cervical dislocation under isoflurane anesthesia. All samples were centrifuged immediately at 14,000 rpm for 10 min at 4 °C, and then stored at −80 °C until analysis.

### 2.3 Sample preparation

For chemical characterization and drug administration, CS (50 g) was extracted three times (each for 2 h) with 500 mL of 75% ethanol under heating reflux. All of the extract solutions were combined and evaporated to bring the final concentration to 0.6 g/mL (equivalent to the weight of CS) at 37 °C under reduced pressure, and the extracts were then stored at 4 °C before use.

An aliquot of 1 mL plasma samples were treated with 3 mL acetonitrile and vortex mixed for 3 min to precipitated protein. After centrifuging at 14,000 rpm for 10 min, the supernatant was dried under nitrogen gas at room temperature. The residue was dissolved in 100 μL methanol before UPLC-Q-TOF/MS and UPLC-QQQ-MS analysis.

The urine samples were loaded on pre-activated HLB columns (6 cc, 200 mg, Waters Oasis, Ireland) directly, then washed off by 2 mL 5% methanol and eluted by 2 mL 95% methanol. The 95% methanol eluate was collected and dried under nitrogen gas at room temperature. The residue was reconstituted in 200 μL methanol before UPLC-Q-TOF/MS analysis.

For quantification of 9 compounds in rat plasma, *p*-coumaric acid, caffeic acid, ferulic acid, isorhamnetin-7-O-glucoside, kaempferol, quercetin, isorhamnetin, hyperoside and cuscutamine and icariin (internal standard) were accurately weighed and dissolved in methanol to construct the stock solutions of 0.1 mg/mL. Working solutions containing the nine analytes were prepared by mixing the respective stock solutions. These mixtures were then serially diluted with methanol to achieve the desired concentration ranges. The internal standard (IS) working solution was prepared in methanol at a concentration of 20 ng/mL. All solutions were stored at 4 °C. The calibration standard and quality control (QC) were prepared by spiking the working solution and IS solution into blank plasma.

### 2.4 UPLC-Q-TOF/MS conditions for rat plasma and urine sample

The Waters Acquity™ UPLC system (Waters, Milford, MA, USA) was employed for chromatographic separation utilizing a Waters Acquity UPLC BEH C18 analytical column (100 mm × 2.1 mm, 1.7 μm). ‌The mobile phase consisted of two solvents: mobile phase A comprising 0.1% formic acid in aqueous solution and mobile phase B containing 0.1% formic acid in acetonitrile. These phases were delivered at a flow rate of 0.3 mL/min by using a linear gradient program as follows: 0–3.5 min, 2%–15% B; 3.5–12.5 min, 15%–36% B; 12.5–19.5 min, 36%–55% B; 19.5–23.5 min, 55%–80% B; 23.5–26 min, 80%–100% B; 26–28 min, 100% B; 28–29 min, 100%–2% B; 29–30 min, 2% B.

MS data were collected in the positive and negative electrospray ionization (ESI) modes through Fast-DDA mode. This automated acquisition strategy prioritized precursor ions based on signal intensity for subsequent MS/MS fragmentation, with data-dependent acquisitions specifically designed to support the construction of molecular network. Instrument parameters were optimized as follows: mass detection range 50–1500 Da; capillary voltages 3.0 kV (positive) and 2.1 kV (negative); sample cone voltage maintained at 35 V; ionization source temperature 140 °C; desolvation temperature 500 °C; cone gas flow rate 50 L/h; desolvation gas flow 1000 L/h. A dual-collision energy ramp was implemented with low-mass-range energies (6–30 eV) and high-mass-range energies (40–90 V). Real-time system calibration employed leucine-enkephalin solution as LockSpray™ reference (5 μL/min infusion), monitoring *m/z* 556.2771 [M+H]^+^ in positive mode and *m/z* 554.2615 [M − H]^−^ in negative mode. Acquisition thresholds were configured to trigger MS/MS mode when total ion current (TIC) exceeded 5000 intensity/s, reverting to full-scan MS mode after five consecutive MS/MS events. Both full-scan MS and MS/MS acquisitions used 0.2 s dwell times, with the system automatically selecting the top five most intense ions per survey scan for fragmentation. All experimental control and data recording were managed through MassLynx 4.2 software (Waters Corporation, USA).

### 2.5 UPLC-QQQ-MS conditions for rat plasma sample

For chromatographic separation, a Waters Acquity™ UPLC system (Waters, Milford, MA, USA) equipped with the Waters Acquity UPLC BEH C18 column (100 mm × 2.1 mm, 1.7 μm) was used. The mobile phase, delivered at a constant flow rate of 0.3 mL/min, was composed of (A) 0.1% formic acid in water and (B) 0.1% formic acid in acetonitrile. The gradient elution program was operated as follows: 0–0.5 min, 2%–15% B; 0.5–1.5 min, 15%–28% B; 1.5–4.5 min, 28%–36% B; 4.5–5.5 min, 36%–55% B; 5.5–6.5 min, 55%–80% B; 6.5–7.0 min, 80%–100% B; 7.0–7.5 min, 100% B; 7.5–8.0 min, 100%–2% B; 8.0–9.0 min, 2% B.

Mass spectrometric detection was performed using a Xevo TQ-S micro triple quadrupole mass spectrometer (Waters, Milford, MA, USA) equipped with an electrospray ionization (ESI) source and controlled by MassLynx 4.2 software (Waters). The MS parameters were optimized as follows: capillary voltage, 3.0 kV for both ESI^+^ and ESI^−^ modes; source temperature, 120 °C; desolvation temperature, 550 °C; desolvation gas flow, 1000 L/h; and cone gas flow, 150 L/h. Analytes were detected in multiple reaction monitoring (MRM) mode with positive/negative polarity switching. Specific MRM transitions, cone voltages, and collision energies for each compound are provided in **Table S1**.

### 2.6 MATLAB-assisted UPLC data processing

**Fig. 2:**
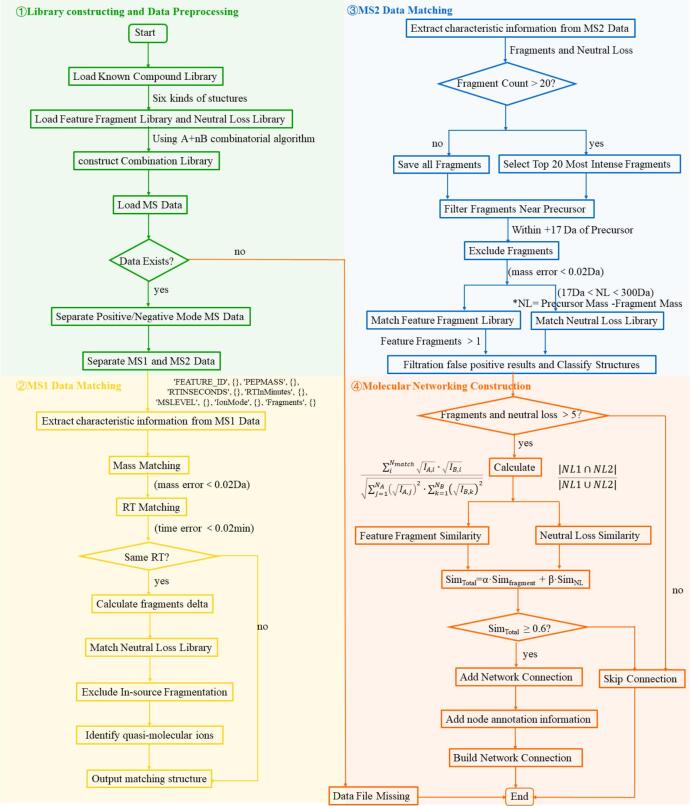
Illustration for the development of a MATLAB-assisted automated data processing and visualization methodology.

As shown in Fig. 2, a MATLAB-assisted automated data processing and visualization methodology was developed. The specific construction ideas and parameter settings are as follows.

#### 2.6.1 Predictive database construction for compounds of CS

**Fig. 3:**
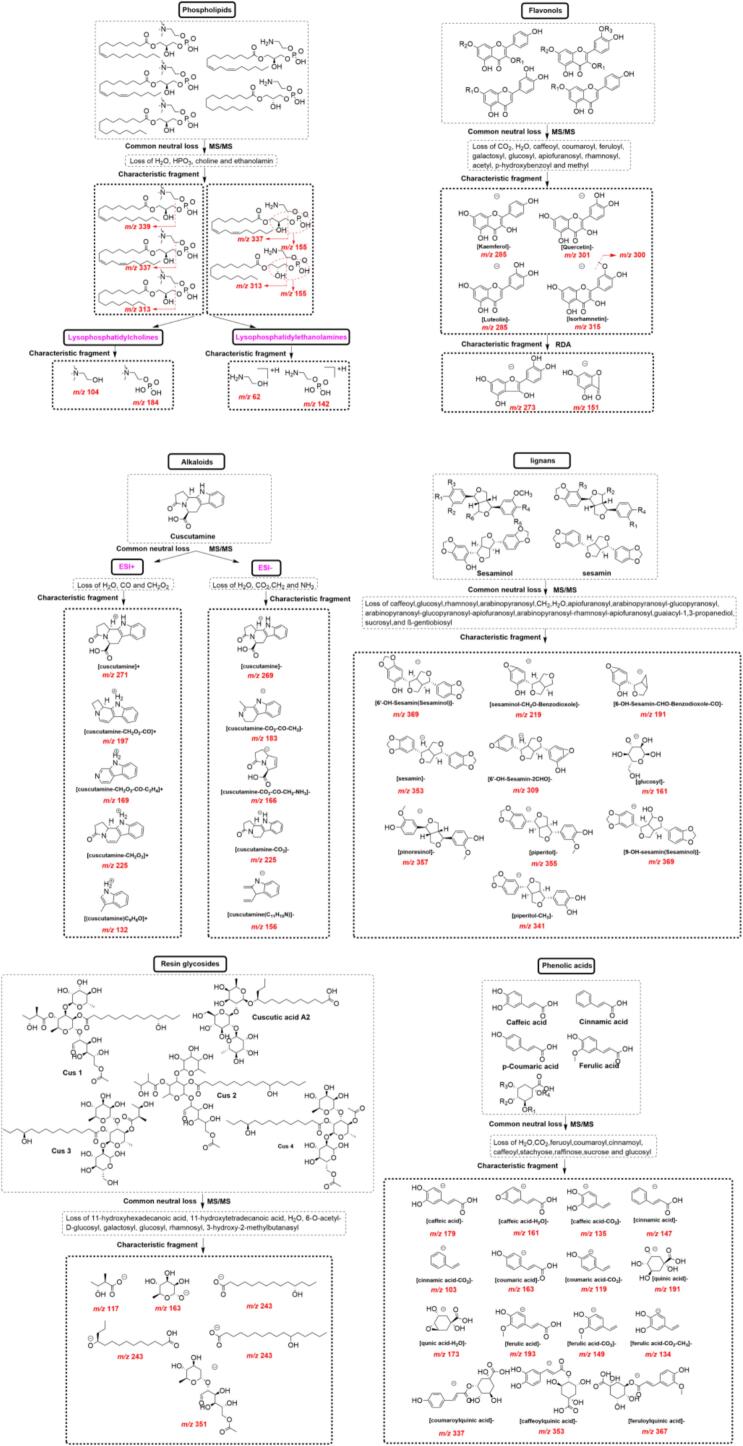
Characteristic fragments and neutral losses of phenolic acids, flavonols, resin glycosides, lignans, alkaloids and phospholipids in *Cuscuta* seeds.

According to literature and reference standards, CS related databases including reported compounds (**Fig. S1** and **Table S2**), parent molecules (A) plus group fragment (nB) combination compounds (**Fig. S2**), diagnostic ions and netural losses (Fig. 3) were established in MATLAB R2024a (MathWorks, USA).

Structural types, exact masses, formulas and references of 154 known constituents (flavonols, phenolic acids, lignans, phospholipids, resin glycosides, alkaloids, etc) from CS were listed in **Table S2**. In the MATLAB setup program, the precursor ions ([M+H]^+^, [M + Na]^+^, [M + NH_4_]^+^ [M−H]^−^, and [M+HCOO]^−^) of reported compounds are automatically generated to form a library of known compounds.

All possible combinations were generated by the “A + nB” combinatorial algorithm by adding, for each parent molecules, from 0 to n groups (*n* ≤ 5), calculating the molecular formula and the precursor ions of each combination ([M+H]^+^, [M + Na]^+^, [M−H]^−^, and [M+HCOO]^−^), in order to generate a combinatorial library with 91,908 structures, containing 8008 flavonols, 1260 phenolic acids, 504 phospholipids, 68544 lignans, 448 alkaloids, and 13,104 resin glycosides.

The diagnostic ions (ESI^+^: 41, ESI^−^: 46) and neutral losses (ESI^+^: 39, ESI^−^: 49) of six major structural types (flavonols, phenolic acids, lignans, phospholipids, resin glycosides, and alkaloids) were collated based on the MS/MS information reported in the literature and MS/MS information of 20 reference standards, and finally constructed into a library in CSV format (names, exact mass, and molecular formulae of the diagnostic ions and neutral losses).

#### 2.6.2 Automated MS1/MS2 annotation workflow

The mass-to-charge ratios (*m*/*z*) of MS1 and MS2 features were extracted by MZmine 2.53. The exact mass of the compounds in each ion mode was compared with the list of precursor ions (MS1 features) to screen the precursor ions with mass differences within tolerance (≤ 0.02 Da), and the mass differences were calculated for the MS1 information with the same retention time (≤ 0.02 min) and matched with a neutral loss library to exclude the false-positive ions caused by in-source cleavage, and to output the preliminary prediction results.

MS information on the first 20 strongly responsive MS2 fragment ions was extracted, and the data were further matched with the libraries of diagnostic ions and neutral losses to output compound identification and classification results.

#### 2.6.3 Molecular networking and visualization

The similarity between two features was calculated based on feature fragment and neutral loss, and molecular network connections were made between compounds with similarity greater than 0.6.

The common feature fragments and neutral loss of the compounds were weighted by the similarity algorithm with the following equation:

$$\mathrm{Similarity}=\alpha \times {\mathrm{Sim}}_{\mathrm{fragment}}+\beta \times {\mathrm{Sim}}_{\mathrm{neutral loss}}$$

Similarity represents the dual-dimension similarity score; α, β are the weight coefficients of each item, respectively; *Sim*_*fragment*_ denotes the similarity of feature fragment; *Sim*_*neutral loss*_ denotes the similarity of neutral loss.

The calculated equation for the similarity of feature fragment is as follows:

$${\mathrm{Sim}}_{\mathrm{fragments}}=\frac{\sum_{i}^{N_{\mathrm{match}}}\sqrt{I_{A,i}}∙\sqrt{I_{B,i}}}{\sqrt{\sum_{j=1}^{N_{A}}{\left(\sqrt{I_{A,j}}\right)}^{2}∙\sum_{k=1}^{N_{B}}{\left(\sqrt{I_{B,k}}\right)}^{2}}}$$

*N*_*match*_ is the number of matched fragment ion pairs; *I*_*A,i*_ and *I*_*B,i*_ *denote* the intensity values of the i-th pair of matched fragments in MS spectrogram A and MS spectrogram B, respectively; *N*_*A*_ represents total number of all valid fragment ions in MS spectrogram A; *N*_*B*_ represents total number of all valid fragment ions in MS spectrogram B.

The calculated equation for the similarity of neutral loss is as follows:

$${\mathrm{Sim}}_{\text{neutral loss}}=\frac{|\mathrm{NL}1\cap \mathrm{NL}2|}{|\mathrm{NL}1\cup \mathrm{NL}2|}$$

*NL1* denotes the neutral loss set of node 1; NL2 denotes the neutral loss set of node 2; *|NL1∩NL2|* is the number of neutral losses common by the two nodes; *|NL1∪NL2|* is the number of all neutral losses of both nodes.

#### 2.6.4 *In vivo* chemometric analysis for exogenous component prediction

The raw LC–MS data for CS group and blank group of plasma/urine samples were processed by OPLS-DA models. The prototypes and metabolites in CS group can be clearly displayed as the dots in the S-plot (VIP ≥ 1.5 and *p* < 0.05). The combinatorial library of representative compounds and common Phase I/II metabolic reactions (+2H, +O, +CH_2_, +SO_3_, +GluA, etc) was established. The precursor ions of each combination ([M+H]^+^, [M + Na]^+^, [M−H]^−^, and [M+HCOO]^−^) were automatically calculated in the MATLAB analysis system. The precursor ions and MS2 data from VIP ≥ 1.5 and *p* < 0.05 were further matched with the libraries of combinatorial metabolites, diagnostic ions and neutral losses to output prototypes and metabolites.

### 2.7 Data processing workflow using UNIFI and SIRIUS software

**UNIFI (v1.9):** Constituent identifications were performed using UNIFI 1.9 (Waters, Milford, MA, USA). The in-house library with 154 known constituents from CS was input to UNIFI. Key parameters with in UNIFI were set as follows: peak detection time, 0–25 min; intensity threshold, 5000 and 500 counts for low energy and high energy acquisitions, respectively; and mass accuracy ±10 ppm.

**SIRIUS (v4.9.12):** Spectral data were processed using text files containing both MS and MS/MS spectral data exported from MZmine 2.53, which included *m/z* values and corresponding relative intensities (%). For molecular formula prediction, parameters were adjusted as follows: instrument type designated as Q-TOF, mass accuracy constrained to 5 ppm, and ionization modes specified as [M+H]^+^, [M + Na]^+^, [M−H]^−^, and [M+HCOO]^−^. Elemental composition searches were limited to C, H, N, P, and O, with a maximum of 10 candidate formulas generated per analysis. Structural annotation *via* CSI: Finger ID incorporated database queries against Natural Products, PubChem, and PubMed.

### 2.8 Effects of H_2_O_2_-induced R2C Leydig cell injury on progesterone levels

#### 2.8.1 Cell culture

Rat Leydig R2C cell lines were maintained in Dulbecco's Modified Eagle Medium/Nutrient Mixture F-12 (DMEM-F12) supplemented with 15% horse serum, 2.5% fetal bovine serum (FBS), 1% penicillin/streptomycin solution, sodium pyruvate, and sodium bicarbonate. Cultures were incubated under controlled conditions (37 °C and 5% CO_2_).

#### 2.8.2 Cell viability assay

Cellular viability was assessed through MTT assay. Cells were plated in 96-well microplates at 1 × 10^4^ cells/well and exposed to series concentrations of compounds and CS extracts for 24 h. 20 μL MTT was introduced to each well and incubated for 4 h. After careful removal of culture media, formazan crystals were solubilized with 150 μL DMSO per well. Absorbance measurements were recorded at 490 nm using a microplate reader (BioTeck, USA).

#### 2.8.3 Progesterone quantification

For quantification of progesterone, cell cultures were challenged with 125 μM H_2_O_2_ alongside 9 compounds and CS extracts for 6 h. Conditioned media samples were collected and clarified by centrifugation (400 ×*g*, 5 min, 4 °C), with supernatants stored at −20 °C until analysis. Progesterone levels were determined *via* UPLC-QQQ-MS. The standard solution preparation and UPLC-QQQ-MS condition were shown in **Support Information S1**.

#### 2.8.4 Statistical analysis

The *in vivo* metabolic experiment was conducted through statistical analysis of results from five rats. The bioactivity evaluation was conducted through statistical analysis of results from five replicate experiments. Experimental results are presented as mean values with standard deviations. Intergroup comparisons were performed through one-way analysis of variance (ANOVA) with post-hoc Tukey test using GraphPad Prism software (Version 6.0, GraphPad Software, USA). Statistical significance was defined at *p* < 0.05.

## 3 Results

### 3.1 The flowchart for identification of absorbed and bioactive compounds in CS

As shown in Fig. 1, MATLAB automated MS annotation, *in vivo* chemometric screening, and progesterone assessment under oxidative stress was established to identify absorbed bioactive compounds in CS. The main workflow was divided into the following four steps:

First, a UPLC-Q-TOF-MS system was used for untargeted chemical and metabolic profiling of *Cuscuta* seeds. Data for MS1 and data-dependent MS2 were acquired in both positive and negative ionization modes. This data was then converted to mzML format using MZmine, which facilitated for noise reduction, peak alignment, and feature extraction. The standardized data was further processed in MATLAB to create a list of precursor ions.

Second, a MATLAB script was developed in-house to automate the annotation of MS1 data. A customized database containing exact masses of known CS compounds (*e.g.*, flavonols, phenolic acids, etc) and potential “Parent molecules + nGroup” (*e.g.*, kaempferol-acyl derivatives) was constructed. The MS1 data were then matched against this library using a mass error tolerance of ≤0.02 Da and a retention time alignment of ±0.02 min. To improve accuracy, source-induced fragments (such as the neutral loss of 162 Da for hexose) were automatically filtered out, which significantly reduced the number of false positives compared to manual workflows (Fig. 2).

Third, the diagnostic ions and neutral loss matching analysis of the MS2 spectra were performed using the MATLAB analysis system platform. The results of identification and classification of compounds were generated based on these matching results. Molecular networks were constructed using a dual-dimension similarity score, which helped reveal structural clusters. Additionally, the accuracy of MATLAB was compared to that of commercial software such as UNIFI and SIRIUS for identifying chemical substances in both positive and negative ion modes.

Finally, the OPLS-DA model compared plasma/urine samples from CS-treated rats with blank control samples. Components with VIP ≥ 1.5 and *p* < 0.05 were screened as potential exogenous markers. It is important to note that phenolic acids and flavonols typically undergo extensive phase II metabolism *in vivo*, resulting in the formation of sulfated (+SO_3_) and glucuronidated (+GluA) metabolites (Heleno et al., 2015; Jiang & Hu, 2012). To annotate these prototypes and metabolites, MATLAB scripts were used to integrate representative compounds and metabolic reactions (*e.g.*, +2H, +O, +CH_2_, +SO_3_, +GluA, *etc.*). Additionally, the effects of the major absorbed components, as well as the parent components of their metabolites, on progesterone levels were evaluated using an H_2_O_2_ induced R2C cell injury model.

### 3.2 Identification of CS components by using MATLAB analysis system platform

**Fig. 4:**
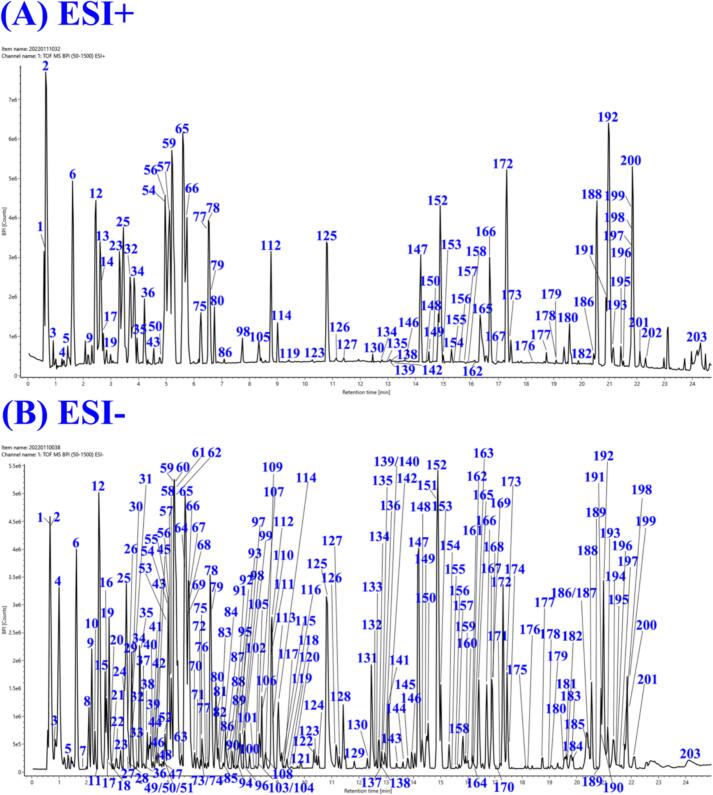
The base peak ion chromatograms (BPI) of *Cuscuta* seed by UPLC-Q-TOF/MS in (A) positive ion mode and (B) negative ion mode.

**Fig. 5:**
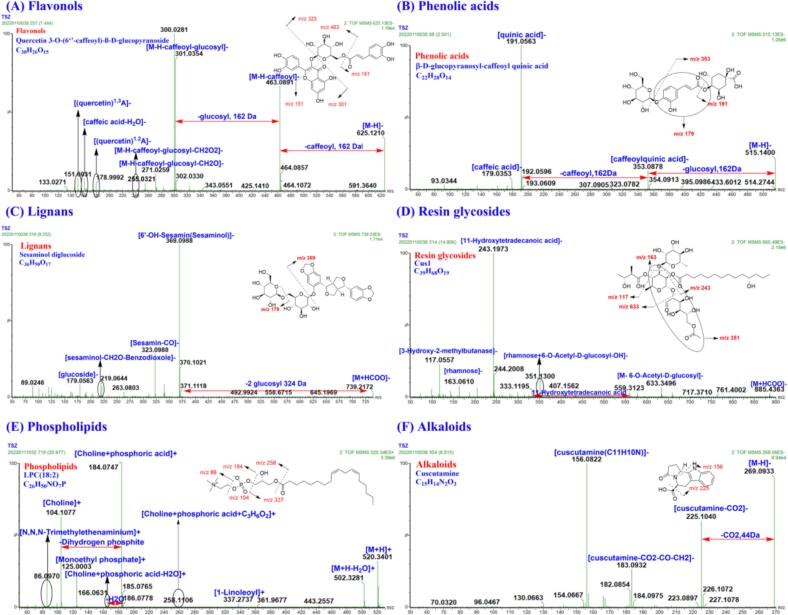
An example demonstrating the MATLAB Platform by using characteristic fragments and neutral losses. (A) Flavonols: Quercetin 3-O-(6″-caffeoyl)-β-D-glucopyranoside; (B) Phenolic acids: β-d-glucopyranosyl-caffeoyl quinic acid; (C) Lignans: Sesaminol diglucoside; (D) Resin glycosides: Cus1; (E) Phospholipids: LPC(18:2); (F) Alkaloids: Cuscutamine.

**Fig. 6:**
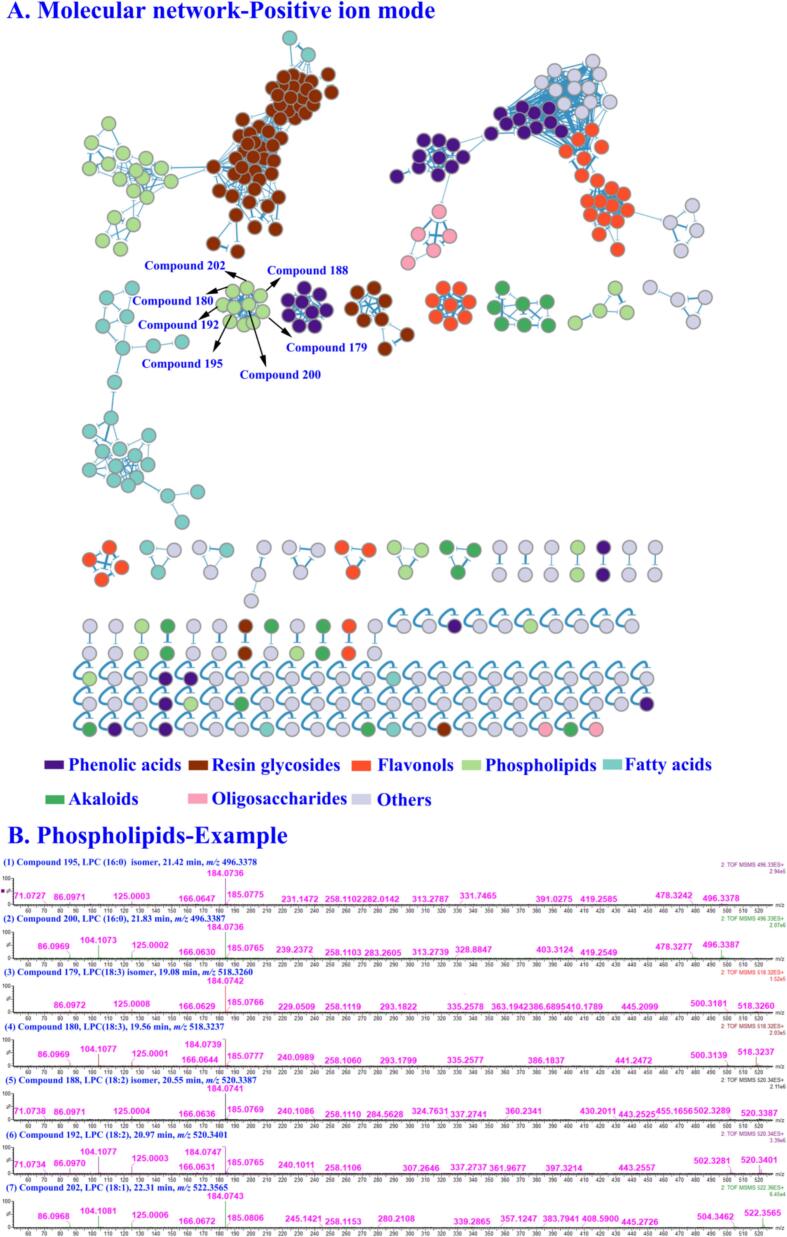
(A) The molecular network for 7 structure types in positive ion mode. (B) The illustration for the phospholipid similarity elucidation based on the MS/MS spectra.

**Fig. 7:**
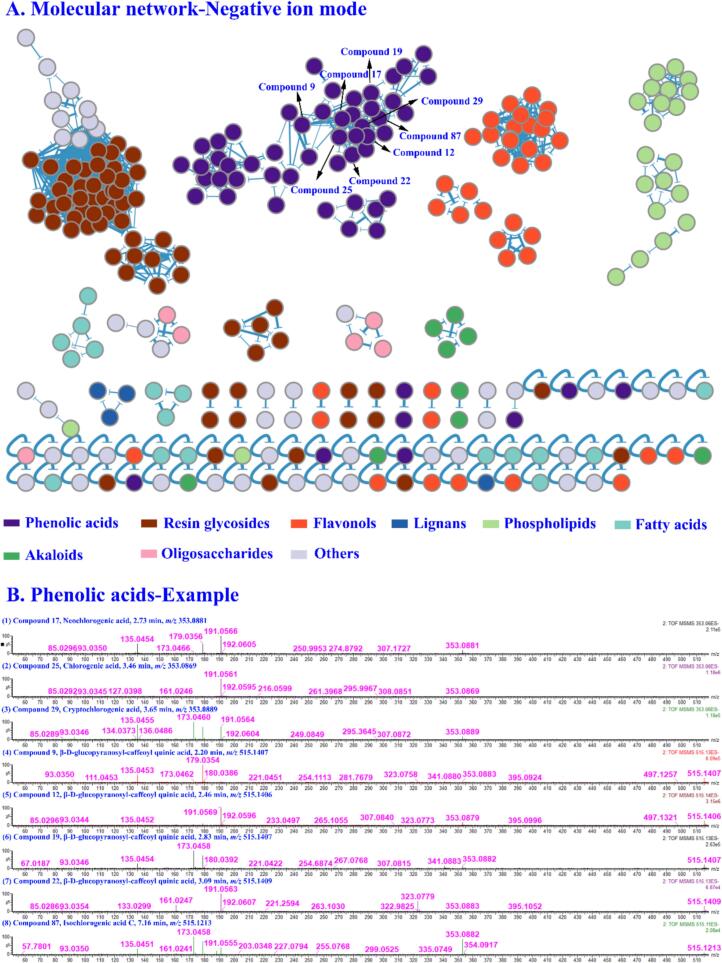
(A) The molecular network for 8 structure types in negative ion mode. (B) The illustration for the phenolic acid similarity elucidation based on the MS/MS spectra.

Based on the CS compound database, the MS1 and MS2 data of CS extracts in positive and negative ion modes were analyzed by taking the MATLAB automatic analysis system. In this study, 1881 and 744 features were obtained by MZmine deconvolution in the positive and negative ion modes, respectively. Then, 368 and 295 features were screened by MZmine excluding isotopic peaks and having MS2 spectra, respectively. Finally, 291 and 215 features were screened to identify using MATLAB, excluding in-source cleavage and mass difference, respectively. As a result, a total of 203 compounds, including 69 phenolic acids, 43 resin glycosides, 32 flavonols, 20 fatty acids, 10 phospholipids, 8 alkaloids, 8 oligosaccharides, 6 lignans, and 7 others, were characterized, among which 20 peaks were further confirmed by comparison with reference standards. The diagnostic ions and neutral losses of phenolic acids, flavonols, resin glycosides, lignans, alkaloids and phospholipids in CS are shown in Fig. 3. The base peak ion chromatograms (BPI) of CS by UPLC-Q-TOF/MS in positive and negative ion modes are shown in Fig. 4. Major fragmentation pathways proposed for the representative components are exhibited in Fig. 5. In addition, phenolic acids, resin glycosides, phospholipids, flavonols, fatty acids, and oligosaccharides were formed molecular clusters based on calculation of the combined similarity values of fragment ions and neutral loss. The MS molecular networks in the positive and negative ion mode are displayed in Fig. 6 and Fig. 7, respectively.

#### 3.2.1 Characterization of phenolic acids

In total, 69 phenolic acids were characterized in CS extract. Neutral losses of H_2_O (18.0106 Da), CO_2_ (43.9898 Da), feruoyl (176.0473 Da), coumaroyl (146.0368 Da), cinnamoyl (130.0419 Da), caffeoyl (162.0317 Da), stachyose (648.2113 Da), raffinose (486.1585 Da), sucrose (324.1057 Da) and glucosyl (162.0528 Da) of phenolic acids were observed by analysis of reference standards and literature. In addition, diagnostic ions at *m/z* 179.0344 ([caffeic acid]^−^), 147.0446 ([cinnamic acid]^−^), 163.0395 ([coumaric acid]^−^), 191.0556 ([quinic acid]^−^), 173.045 ([quinic acid-H_2_O]^−^), 193.0501 ([ferulic acid]^−^), 337.0923 ([coumaroylquinic acid]^−^), 353.0873 ([caffeoylquinic acid]^−^) and 367.1029 ([feruloylquinic acid]^−^) were usually detected in MS/MS spectra. Diagnostic ions and neutral losses of phenolic acids were shown in Fig. 3.

Here, taking compound **12** as an example, the molecular formula was initially postulated as C_22_H_28_O_14_ (*m/z* 515.1406, t_R_ = 2.46 min, mass error = 3.8 mDa). In MS2 spectra, the fragment ion at *m/z* 353.0378 [caffeoylquinic acid]^−^ was generated by neutral loss of glucose, and a fragment ion of parent molecule at *m/z* 191.0563 [quinic acid]^−^ was generated by further loss of caffeoyl. In addition, the diagnostic ion at *m/z* 179.0353 [caffeic acid]^−^ was produced (Fig. 5B). Compound **12** was a phenolic acid and eventually identified it as β-d-glucopyranosyl-caffeoyl quinic acid. According to the Fig. 7, it could be observed in the molecular network with seven highly similar neighboring nodes (compounds **9**, **17**, **19**, **22**, **25**, **29** and **87**). Among them, compounds **9** (t_R_ = 2.20, *m/z* 515.1407), **19** (t_R_ = 2.83, *m/z* 515.1407) and **22** (t_R_ = 3.09, *m/z* 515.1409) had the same the molecular formula with compound **12** after automate MS1 data annotation. The diagnostic ions at *m/z* 353, 191, 179, 173, 135 and 93 were generated to determine their structural type as phenolic acids. Taken together, they were isomers of compound **12**. They were also characterized as β-d-glucopyranosyl-caffeoyl quinic acid. In addition, compounds **17** (t_R_ = 2.73, *m/z* 353.0881, C_16_H_18_O_9_), **25** (t_R_ = 3.46, *m/z* 353.0869, C_16_H_18_O_9_), **29** (t_R_ = 3.65, *m/z* 353.0889, C_16_H_18_O_9_) and **87** (t_R_ = 7.16, *m/z* 515.1213, C_25_H_24_O_12_) had similar diagnostic ions and neutral losses as the above compounds. Based on MATLAB automated MS data analysis platform and comparing with reference standards, they were identified as neochlorogenic acid, chlorogenic acid, cryptochlorogenic acid and isochlorogenic acid C, respectively.

The MS1 and MS2 data of compound **46** were extracted based on Matlab automated analysis platform system. In the negative ion mode, the MS1 data showed the precursor ion [M−H]^−^ as *m/z* 367.1039 with a retention time of 4.62 min. Comparison with the information in the combinatorial library list further led to the preliminary identification of feruloylquinic acid (C_17_H_20_O_9_). Matching the MS2 data with the diagnostic ion and neutral loss databases revealed the fragment ions *m/z* 191.0562. [quinic acid]^−^ and *m/z* 173.0459 [quinic acid-H_2_O]^−^, as well as a neutral loss of 176 Da (feruoyl) (**Fig. S3**). Therefore, compound **46** was a phenolic acid, and it was finally identified as feruloylquinic acid. Among them, the diagnostic ions at *m*/*z* 184, 166, 104 and 86 were all produced.

#### 3.2.2 Characterization of resin glycosides

In this work, 43 resin glycosides were characterized in CS. The diagnostic ion at *m/z* 351.1291 [rhamnose+acetyl-glucosyl-OH-H]^−^, 271.2273 [11-hydroxyhexadecanoic acid-H]^−^, 243.1960 [11-hydroxyhexadecanoic acid-H]^−^, and 117.0557 [3-Hydroxy-2-methylbutanase-H]^−^. Diagnostic ions and neutral losses of resin glycosides were shown in Fig. 3.

The MS1 spectra of compound **152** in the negative ion mode was analyzed by MATLAB analysis system and the quasimolecular ion [M+HCOO]^−^ was determined as *m/z* 885.4331 (C_39_H_68_O_19_, t_R_ = 14.89 min). The system was tentatively predicted to be Cus 1. In MS2 spectra, the fragment ion at *m/z* 633.3496 [M−acetyl-glucosyl-H]^−^ was generated by the loss of acetylated glucose. Then, generation of fatty acid chain fragment ion at *m/z* 243.1973 [11-hydroxytetradecanoic acid-H]^−^ and glycosyl fragment ion at *m/z* 351.1300 [rhamnose+acetyl-glucosyl-OH-H]^−^ by glycosidic bond breakage. Further cleavage yielded fragment ions at *m/z* 163.0610 and 117.0557 (Fig. 5D). Based on the diagnostic ion library, compound **152** was identified as a resin glycoside, Cus 1.

#### 3.2.3 Characterization of flavonols

A total of 32 flavonols were detected in CS. In the negative ion mode, the flavonols eliminated the saccharide and phenolic acid groups to obtain the base peak ions at *m/z* 285.0399 [kaemferol-H]^−^, 301.0348 [quercetin-H]^−^, and 315.0505 [isorhamnetin-H]^−^. The diagnostic ion at *m/z* 151.0031 was yielded *via* RDA cleavage. In the positive ion mode, the diagnostic ions at *m/z* 287.0556 [kaemferol+H]^+^, 303.0505 [quercetin+H]^+^, and 317.0661 [isorhamnetin+H] ^+^ by loss of saccharide and phenolic acid groups. The characteristic ion at *m/z* 153.0188 was produced *via* RDA cleavage. Diagnostic ions and neutral losses of flavonols were shown in Fig. 3.

In MS1 spectra, compound **93** showed a quasi-molecular ion [M−H]^−^ at *m/z* 625.1198 (C_30_H_26_O_15_) with a retention time of 7.43 min. As shown in Fig. 5A, the fragment ion at *m*/*z* 463.0891 [M−H-caffeoyl]^−^ was produced by the loss of caffeoyl. Another aglycone ion at *m*/*z* 301.0354 [quercetin-H]^−^ was generated by the continuous loss of glucose. The diagnostic ions at *m/z* 151.01 and 178.9992 were yield by RDA cleavage from the positions 1, 3 and positions 1, 2 in C-ring, respectively. Based on the known compound library, the diagnostic ion library and the neutral loss library, it was characterized as a flavonol, quercetin 3-O-(6″-caffeoyl)-β-D-glucopyranoside.

As shown in **Table S3** and **Fig. S4**, compound **81** exhibited a protonated ion [M−H]^−^ at *m/z* 447.0930 (C_21_H_20_O_11_) with a retention time of 6.81 min. The product ions of *m/z* 285.0399 [kaempferol-H]^−^, 255.0304 [Kaempferol-CH_2_O-H]^−^ and 227.0350 [Kaempferol-CH_2_O-CO-H]^−^ were produced in MS/MS spectra. The diagnostic ion at *m/z* 151.0046 was produced by RDA cleavage. Based on combination compound library, compound **81** was identified as a flavonol, kaempferol-O-galactoside.

#### 3.2.4 Characterization of phospholipids

In total, 10 phospholipids were identified in CS. In the positive ion mode, phospholipids were apt to produce the typical fragment ions at *m/z* 337.2743 [1-Linoleoyl+H]^+^, 313.2743 [1-Palmitoyl+H]^+^, 184.0739 [Choline+phosphoric acid+H]^+^, 166.0633 [Choline+phosphoric acid-H_2_O + H]^+^, 125.0004 [Monoethyl phosphate+H]^+^, 104.1075 [Choline+H]^+^ and 86.0970 [*N*,*N*,*N*-Trimethylethenaminium+H]^+^. Diagnostic ions and neutral losses of phospholipids were shown in Fig. 3.

As shown in Fig. 5E, compound **192** showed a quasimolecular ion [M+H]^+^ at *m/z* 520.3423 (C_26_H_50_NO_7_P) with a retention time of 20.97 min. In MS2 spectra, the fragment ion at *m/z* 502.3281 [M+H-H_2_O]^+^ was yielded by the loss of H_2_O. Then, the fatty acid chain fragmentation ion at *m/z* 337.2737 [1-Linoleoyl]^+^ was produced. Specially, the strong diagnostic ions at *m/z* 184.0747 [Choline+phosphoric acid+H]^+^, *m/z* 166.0631 [Choline+phosphoric acid-H_2_O + H]^+^, 125.0003 [Monoethyl phosphate+H]^+^ and 104.1077 [Choline+H]^+^ were derived from phosphocholine and choline. Furthermore, the other common ion at *m/z* 86 [*N*,*N*,*N*-Trimethylethenaminium+H]^+^ by the continuous loss of H_2_O. According to the known compound library, the diagnostic ion library and the neutral loss library, it was characterized as a phospholipid, LPC (18:2). As shown in Fig. 6, it could be observed in the molecular network with six highly similar neighboring nodes (compounds **179**, **180**, **188**, **195**, **200** and **202**). The diagnostic ions from above seven compounds at *m/z* 184, 166, 125, 104 and 86 were all produced. Based on MATLAB automated MS data analysis platform, they were identified in **Table S3**.

#### 3.2.5 Characterization of alkaloids

As shown in **Table S3**, 8 indole alkaloids were identified in CS. In the positive ion mode, the diagnostic ions at *m/z* 225.1028, 197.1073, 180.0813, and 169.0766 were formed by successive loss of CH_2_O_2_ (46 Da), CO (28 Da), NH_3_ (17 Da), and C_2_H_4_ (28 Da), respectively. In the negative ion mode, the diagnostic ions at *m/z* 225.1028 and 183.0922 were produced by loss of CO_2_ (44 Da), CO (28 Da) and CH_2_ (14 Da). Diagnostic ions and neutral losses of alkaloids were shown in Fig. 3.

In MS1 spectra, compounds **105** (t_R_ = 8.34 min) and **112** (t_R_ = 8.81 min) showed a protonated molecular ion [M+H]^+^ at *m/z* 271.1084 (C_15_H_14_N_2_O_3_). Fragment ions at *m/z* 225.1032 [M + H-CH_2_O_2_]^+^, 197.1077 [M+H-CH_2_O_2_-CO]^+^, 180.0818 [M + H-CH_2_O_2_-CO-NH_3_]^+^ and 169.0772 [M + H-CH_2_O_2_-CO-C_2_H_4_]^+^ were observed in MS2 spectra (**Fig. S5**). In addition, as shown in Fig. 5F, they gave the [M−H]^−^ ion at *m/z* 269.0938. And their empirical molecular formulas were all supposed as C_15_H_14_N_2_O_3_. The diagnostic ions at *m/z* 225.1040 [M−H-CO_2_]^−^, 183.0932 [M−H-CO_2_-CO-CH_2_]^−^, and 156.0822 [C_11_H_10_N]^−^ were produced in MS2 spectra. Therefore, compound **112** was identified as cuscutamine by comparison with the reference standard and the known compound library. Compound **105** was tentatively characterized as cuscutamine isomer.

#### 3.2.6 Characterization of lignans

In total, 6 lignans were detected in CS. Diagnostic ions and neutral losses of lignans were shown in Fig. 3. The MS1 spectra of compound **117** in negative ion mode was analyzed by MATLAB analysis system and the quasimolecular ion [M + HCOO]^−^ was determined as *m/z* 739.2086 (C_32_H_38_O_17_, t_R_ = 9.25 min). The diagnostic ion at *m/z* 369.0988 [Sesaminol-H]^−^ was yielded by the loss of two glucose. Based on the known compound library, the diagnostic ion library and the neutral loss library, it was characterized as a lignan, sesaminol diglucoside. In addition, compounds **20**, **107**, **113**, **121** and **122** were determined as lignans by MATLAB analysis system. According to the known compound library and the diagnostic ion library, they were identified as cuscutaresinol A/cuscutaresinol B, cuscutoside D, neocuscutoside B, cuscutoside A and cuscutoside B, respectively.

Compared with the six structural types in the pre-database construction process, the oligosaccharide type was added to the identification process. Since fragments such as glucose, xylose, rhamnose, and galactose appear both as parent molecules and as group fragments, a database of oligosaccharide type was formed through the combination of “A + nB”, which led to the emergence of this class of compounds in the later stages of compound identification. Therefore, 8 oligosaccharides were characterized in CS. Moreover, based on the known compound library, 20 fatty acids were tentatively identified. Because the presence of fatty acids in CS has been reported in the literature (as shown in **Table S2**), 20 fatty acids were characterized based on the known compound library.

### 3.3 Fundamental characteristics of the MATLAB analysis system platform

MATLAB automated MS data analysis platform was utilized to improve the accuracy of the quasimolecular ions and to exclude ions produced by in-source cleavage. This cannot be avoided with UNIFI and SIRIUS, resulting in a less accurate process of identifying the components. As an example, the MS1 spectrum of a retention time of 5.60 min showed two highly responsive ions at *m/z* 465.1028 and 303.0534 in positive ion mode (**Fig. S6**). Because in-source cleavage was not considered, the above two ions were determined to be two components by analysis of SIRUS and UNIFI software. However, in the MATLAB automated MS data analysis platform, the *m/z* 303.0534 resulting from in-source cleavage was determined to be a fragment ion of *m/z* 465.1024 by loss of galactoside. Therefore, the quasimolecular ion [M+H]^+^ of compound **65** was *m/z* 465.1028 (C_21_H_20_O_12_). Based on MATLAB automated MS data analysis platform and comparing with reference standards, it was identified as a flavonol, hyperoside. In addition to flavonols, part of the phenolic acids also exhibited in-source cleavage in positive ion mode. The MS1 spectrum of a retention time of 3.45 min showed two highly responsive ions at *m/z* 465.1028 and 303.0534 in positive ion mode (**Fig. S7**). The *m/z* 163.0410 resulting from in-source cleavage was determined to be a fragment ion of *m/z* 355.1031 by loss of quinic acid. Therefore, the quasimolecular ion [M+H]^+^ of compound **25** was *m/z* 355.1031 (C_16_H_18_O_19_). Based on MATLAB automated MS data analysis platform and comparing with reference standards, it was identified as a phenolic acid, Chlorogenic acid. The results indicated that MS1 data showed significant in-source fragmentation, while the MATLAB system platform could effectively reduce false positives and improve the accuracy of identification of features. Therefore, the accuracy of the prediction of compounds by UNIFI, SIRIUS, and our system was analyzed using the MS data in positive and negative ion mode. As shown in **Table S4**, compared with SIRIUS (positive: 34.37%; negative: 50.97%) and UNIFI (positive: 24.86%; negative: 33.33%), the prediction accuracy of MATLAB system improved to 80.07% and 91.63%, respectively.

In addition, UNIFI software is highly dependent on the built-in compound libraries for structural characterization, which makes it difficult to perform preliminary structural characterization of unknown compounds. The MATLAB analysis platform, however, has a built-in library of combinatorial compositions, which, in combination with a library of neutral loss and diagnostic ions, leads to the predictive ability of this system for unknown compounds. Taking compound **98** as an example, its quasimolecular ion [M−H]^−^ in negative ion mode was *m/z* 313.0831 (C_15_H_16_N_2_O_4_) with a retention time of 7.81 min (**Fig. S8**). In the MS1 spectra, the UNIFI and SIRIUS software could not identify the ion at *m/z* 313.0831 because it was an unreported compound. They also misidentified the in-source cleavage ion at *m/z* 269.0938 as a quasimolecular ion, resulting in a misidentification of this compound. Based on MATLAB automated MS data analysis platform, the diagnostic ions at *m/z* 269.0938 [M-H-CO_2_], 225.1040 [M-H-2CO_2_]^−^, 183.0932 [M-H-2CO_2_-CO-CH_2_]^−^, and 156.0822 [C_11_H_10_N]^−^ were produced in MS2 spectra. Therefore, compound **98** was identified as an unreported alkaloid, carboxyl-cuscutamine. As shown in **Table S3**, 47 unreported compounds were discovered by using the analytical platform of this study. The MATLAB system will serve as a powerful tracking tool for the targeted isolation of the above unreported compounds in the future. In comparison with the existing literature (Wang et al., 2022; Ye et al., 2005), the use of MATLAB automated MS data analysis platform did not stop at the discovery of new compounds, but was also able to output the types of unreported compounds, as well as the basic compositions of the parent molecules + group fragments (Especially phenolic acids, resin glycosides and alkaloids). Therefore, the results of above analysis platform are useful for subsequent application in the identification of prototypes and metabolites *in vivo*. Despite the advantages of post MS data processing in this study, there are limitations in the LC-MS conditions for comprehensive characterization of components of CS, such as the chromatographic separation and isomerism analysis.

MATLAB automated MS data analysis platform calculated the similarity of compounds based on the dual dimensions of feature fragmentation and neutral loss, and applies to match different types of compounds by assigning different weights. This similarity algorithm could improve the similarity of compounds of the same structural type, especially isomer compounds with low intensity of fragment ions. Taking compounds **9** and **22** as an example, with the GNPS algorithm, their similarity was 0.80. And by applying the algorithm of our system, their similarity was improved to 0.85. Therefore, as shown in Fig. 6 and Fig. 7, the same structural type of compounds was more aggregated in the molecular network visualization. As shown in **Table S5** and **Table S6**, Compared with different α and β values, the results showed the selection of weight coefficients for the dual-dimension similarity score (α = 0.9, β = 0.1) enabled the molecular network to incorporate more nodes and edges.

### 3.4 *In vivo* chemometric analysis for screening prototypes and metabolites

**Fig. 8:**
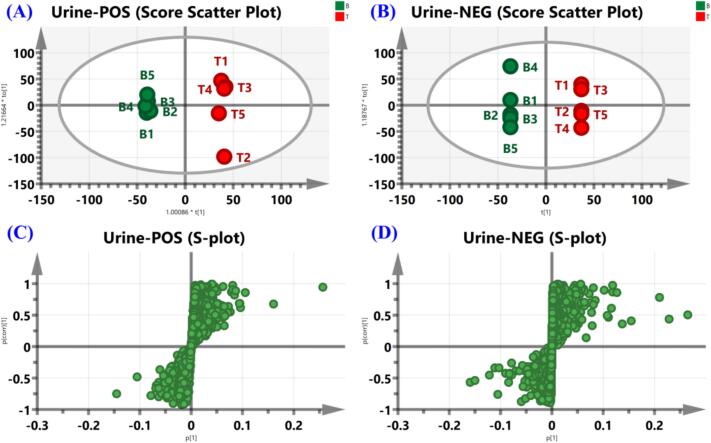
Score Scatter plots and S-plots from orthogonal partial least-squares discriminant analysis (OPLS-DA) models for classifying oral administration of CS group and blank group samples. Score Scatter plots of CS group *vs.* blank group in positive mode (A) and negative mode (B); S-plots in positive mode (C) and negative mode (D). T: CS group, B: blank group.

**Fig. 9:**
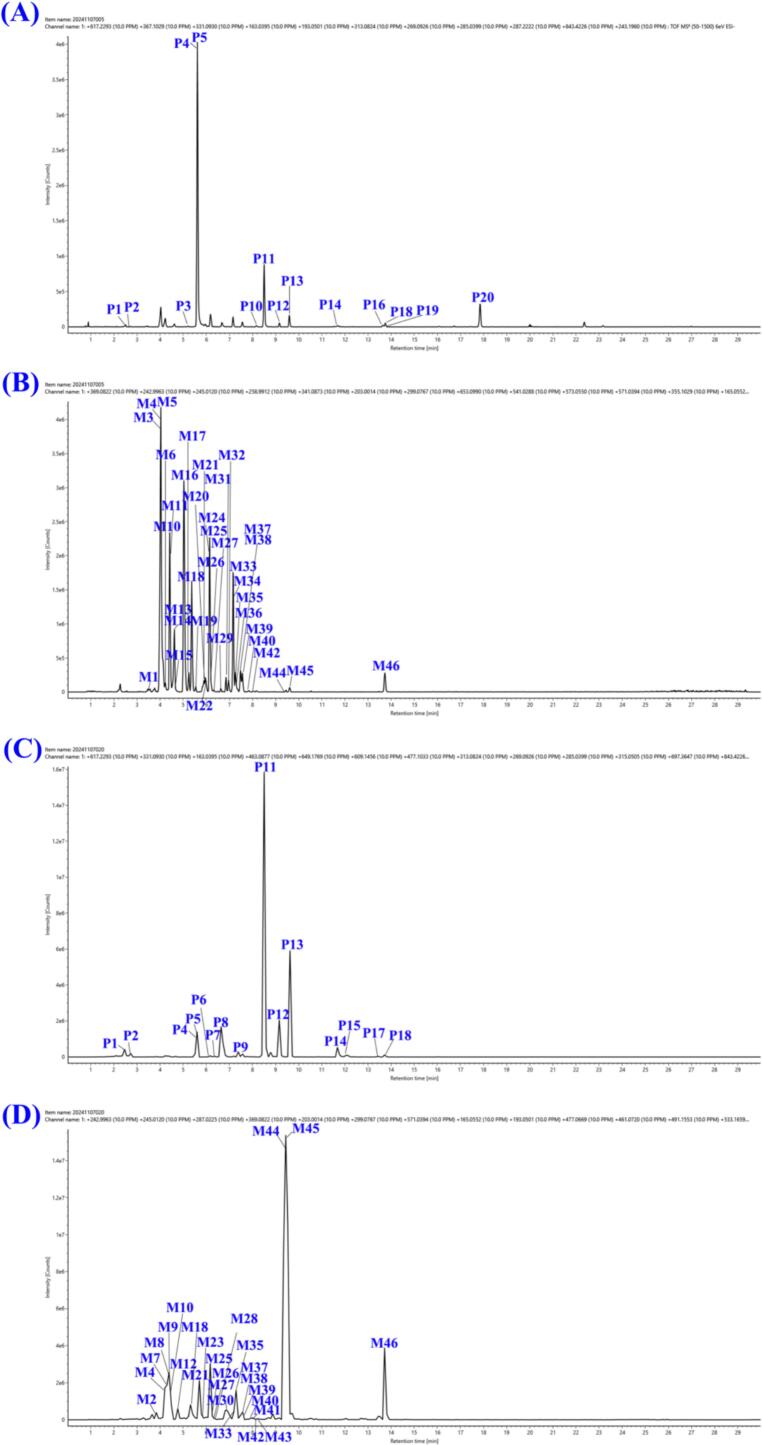
Extracted ion chromatograms (EICs) of prototypes and metabolites in rat plasma and urine (negative ion mode). (A) Prototypes in plasma, (B) Metabolites in plasma, (C) Prototypes in urine, (D) Metabolites in urine.

**Fig. 10:**
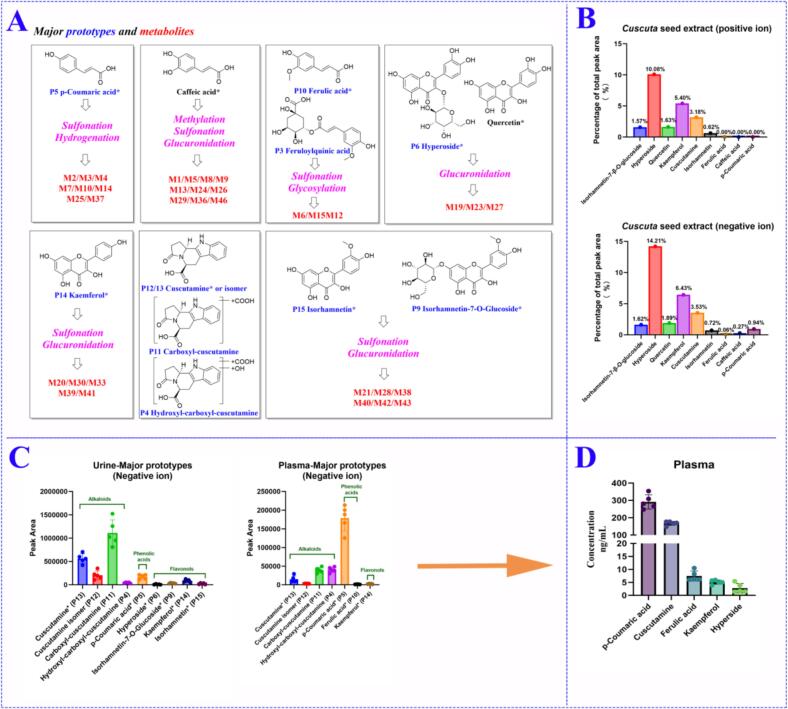
(A) Major prototypes and metabolites in urine and plasma (*Comparison with reference standard). (B) The percentage of major prototypes in *Cuscuta seed* extract. (C) The peak area of major prototypes in urine and plasma samples. (D) The concentration of major prototypes in mixed four points time plasma (*n* = 5).

In this study, a comparison between the data files of CS group and blank group was performed by OPLS-DA. Taking the advantage of data-screening and *in vivo* chemometric analysis of SIMCA 14.0, some ions of prototypes and metabolites embedded in background ions of endogenous interference can be screened. In OPLS-DA scores, CS group and blank group generated different clusters and were clearly separated in the components (Fig. 8A and B). Then, as shown in Score Scatter plots (Fig. 8C and D), components with VIP ≥ 1.5 and *p* < 0.05 were screened as potential exogenous markers. MS1 and MS2 data of potential exogenous markers imported into MATLAB automated MS data analysis. The combinatorial library of representative compounds (*p*-coumaric acid，hyperoside，isorhamnetin-7-O-glucoside，ferulic acid，cuscutamine，kaempferol，isorhamnetin, quercetin, caffeic acid, sesaminol, sesamin, pinoresinol, piperitol, etc) and metabolic reactions (+2H, +O, +CH_2_, +SO_3_, +GluA, etc) was established. The precursor ions of each combination ([M+H]^+^, [M + Na]^+^, [M−H]^−^, and [M + HCOO]^−^) were automatically calculated in the MATLAB analysis system. 20 prototypes and 46 metabolites were identified using MATLAB automated MS data analysis platform. The extracted ion chromatograms (EICs) of prototypes and metabolites in rat plasma and urine are shown in Fig. 9. Major prototypes and metabolites in urine and plasma are displayed in Fig. 10 (negative ion mode). Detailed UPLC-Q-TOF/MS data information for prototypes and metabolites is listed in Table 1 and Table 2, respectively.

**Table 1:** Characterization of prototypes in rat plasma and urine.

NO.	tR (min)	Formula	Selected ion	Measured mass	Calculated mass	Mass error /mDa	MS fragmentation	Identification	Structure	Source
P1	2.50	C_24_H_42_O_18_	[M−H]^−^	617.2299	617.2293	0.6	451.1797, 433.1717, 407.1574, 351.1294, 281.1237, 263.1136, 205.0714, 163.0608, 145.0498, 117.0551	3MePenHex	Oligosaccharides	P/U
P2	2.72	C_24_H_42_O_18_	[M−H]^−^	617.2296	617.2293	0.3	407.1588	3MePenHex	Oligosaccharides	P/U
P3	5.2	C_17_H_20_O_9_	[M−H]^−^	367.1006	367.1029	−2.3	N/A	Feruloylquinic acid	Phenolic acids	P
P4	5.6	C_16_H_16_N_2_O_6_	[M+H]^+^	333.1091	333.1087	0.4	217.0975, 196.0779, 168.0810, 154.0663	hydroxyl-carboxyl-cuscutamine	Alkaloids	P/U
			[M−H]^−^	331.0934	331.093	0.4	287.1013, 269.0903, 225.1033			
P5	5.61	C_9_H_8_O_3_	[M−H]^−^	163.0402	163.0395	0.7	119.0501	p-Coumaric acid⁎	Phenolic acids	P/U
P6	6.17	C_21_H_20_O_12_	[M−H]^−^	463.0869	463.0877	−0.8	301.0343, 300.0277	Hyperoside⁎	Flavonols	U
P7	6.49	C_30_H_34_O_16_	[M−H]^−^	649.1776	649.1769	0.7	487.1255, 179.0357	O-caffeoyl-O-p-coumaroyl-sucrose	Phenolic acids	U
P8	6.82	C_27_H_30_O_16_	[M−H]^−^	609.1464	609.1456	0.8	315.0504	Nelumboroside A	Flavonols	U
P9	7.39	C_22_H_22_O_12_	[M−H]^−^	477.1046	477.1033	1.3	315.0503	Isorhamnetin-7-O-Glucoside⁎	Flavonols	U
P10	8.09	C_10_H_10_O_4_	[M−H]^−^	193.0506	193.0501	0.5	133.0296	Ferulic acid	Phenolic acids	P
P11	8.56	C_16_H_14_N_2_O_5_	[M+H]^+^	315.0974	315.0981	−0.7	269.0932, 225.1033, 223.0878, 167.0708	carboxyl-cuscutamine	Alkaloids	P/U
			[M−H]^−^	313.0838	313.0824	1.4	269.0934, 225.1035, 183.0931, 156.0822			
P12	9.16	C_15_H_14_N_2_O_3_	[M+H]^+^	271.1089	271.1083	0.6	225.1020	Cuscutamine isomer	Alkaloids	P/U
			[M−H]^−^	269.0934	269.0926	0.8	225.1024			
P13	9.59	C_15_H_14_N_2_O_3_	[M+H]^+^	271.109	271.1083	0.7	225.1033	Cuscutamine⁎	Alkaloids	P/U
			[M−H]^−^	269.0932	269.0926	0.6	225.1029, 183.0882, 156.0818			
P14	11.69	C_15_H_10_O_6_	[M−H]^−^	285.0402	285.0399	0.3	N/A	Kaempferol⁎	Flavonols	P/U
P15	12.06	C_16_H_12_O_7_	[M−H]^−^	315.0512	315.0505	0.7	300.0272	Isorhamnetin⁎	Flavonols	U
P16	13.63	C_16_H_32_O_4_	[M−H]^−^	287.2231	287.2222	0.9	243.1964	Dihydroxyhexadecanoic acid	Fatty acids	P
P17	13.45	C_32_H_58_O_16_	[M−H]^−^	697.3671	697.3647	2.4	551.3070, 389.2547, 243.1982	Cuscutic acid A2	Phenolic acids	U
P18	13.70	C_37_H_66_O_18_	[M+HCOO]^−^	843.4183	843.4226	−4.3	N/A	Cus 3	Resin glycosides	P/U
P19	13.75	C_16_H_32_O_4_	[M−H]^−^	287.2231	287.2222	0.9	243.1966	Dihydroxyhexadecanoic acid	Fatty acids	P
P20	17.84	C_14_H_28_O_3_	[M−H]^−^	243.1970	243.1960	1	225.1881	Hydroxytetradecanoic acid	Fatty acids	P

P: Plasma; U: Urine.

⁎ : Confirmed with reference standards.

**Table 2:** Characterization of metabolites in rat plasma and urine.

NO.	tR (min)	Formula	Selected ion	Measured mass	Calculated mass	Mass error /mDa	MS fragmentation	Identification	Structure	Source
M1	3.57	C_16_H_18_O_10_	[M−H]^−^	369.0826	369.0822	0.4	193.0498	Caffeic acid+CH_2_ + GluA	Phenolic acids	P
M2	3.84	C_9_H_8_O_6_S	[M−H]^−^	242.9973	242.9963	1	163.0401	p-Coumaric acid or isomers+SO_3_	Phenolic acids	U
M3	4.03	C_9_H_8_O_6_S	[M−H]^−^	242.9970	242.9963	0.7	163.0400, 119.0499	p-Coumaric acid or isomers+SO_3_	Phenolic acids	P
M4	4.07	C_9_H_10_O_6_S	[M−H]^−^	245.0121	245.0120	0.1	165.0555	p-Coumaric acid+2H + SO_3_	Phenolic acids	P/U
M5	4.12	C_9_H_8_O_7_S	[M−H]^−^	258.9918	258.9912	0.6	179.0344, 135.0448	Caffeic acid+SO_3_	Phenolic acids	P
M6	4.22	C_10_H_10_O_7_S	[M−H]^−^	273.0076	273.0069	0.7	193.0506, 134.0370	Ferulic acid+SO_3_	Phenolic acids	P
M7	4.30	C_9_H_8_O_6_S	[M−H]^−^	242.9968	242.9963	0.5	163.0402	p-Coumaric acid or isomers+SO_3_	Phenolic acids	U
M8	4.39	C_11_H_12_O_7_S	[M−H]^−^	287.0228	287.0225	0.3	207.0661, 163.0764, 122.0371	Caffeic acid+2CH_2_ + SO_3_	Phenolic acids	U
M9	4.39	C_16_H_18_O_10_	[M−H]^−^	369.0828	369.0822	0.6	193.0501	Caffeic acid+CH_2_ + GluA	Phenolic acids	U
M10	4.41	C_9_H_10_O_6_S	[M−H]^−^	245.0127	245.0120	0.7	165.0561, 121.0659	p-Coumaric acid+2H + SO_3_	Phenolic acids	P/U
M11	4.44	C_15_H_18_O_9_	[M−H]^−^	341.0879	341.0873	0.6	N/A	O-caffeoyl-β-D-glucopyranoside	Phenolic acids	P
M12	4.59	C_7_H_8_O_5_S	[M−H]^−^	203.0045	203.0014	3.1	123.0472	Methoxyphenol+SO_3_	Phenolic acids	U
M13	4.62	C_16_H_18_O_10_	[M−H]^−^	369.083	369.0822	0.8	193.0464	Caffeic acid+CH_2_ + GluA	Phenolic acids	P
M14	4.62	C_9_H_8_O_6_S	[M−H]^−^	242.9970	242.9963	0.7	163.0400, 119.0499	p-Coumaric acid or isomers+SO_3_	Phenolic acids	P
M15	4.71	C_10_H_10_O_7_S	[M−H]^−^	273.0075	273.0069	0.6	193.0545, 134.0366	Ferulic acid+SO_3_	Phenolic acids	P
M16	5.03	C_7_H_8_O_5_S	[M−H]^−^	203.002	203.0014	0.6	123.0448	Methoxyphenol+SO_3_	Phenolic acids	P
M17	5.24	C_13_H_16_O_8_	[M−H]^−^	299.0774	299.0767	0.7	123.0449	Methoxyphenol+GluA	Phenolic acids	P
M18	5.37	C_13_H_16_O_8_	[M−H]^−^	299.0772	299.0767	0.5	123.0449	Methoxyphenol+GluA	Phenolic acids	P/U
M19	5.41	C_27_H_26_O_19_	[M−H]^−^	653.1020	653.0990	3	477.0706, 301.0359	Quercetin+2GluA	Flavonols	P
M20	5.83	C_21_H_18_O_15_S	[M−H]^−^	541.0287	541.0288	−0.1	364.9934, 285.0406	Kaempferol+SO_3_ + GluA	Flavonols	P
M21	5.90	C_22_H_20_O_16_S	[M+H]^+^	573.0547	573.055	−0.3	317.0667	Isorhamnetin+SO_3_ + GluA	Flavonols	P/U
			[M−H]^−^	571.0393	571.0394	−0.1	491.0817, 315.0508			
M22	5.95	C_16_H_20_O_9_	[M−H]^−^	355.1035	355.1029	0.6	193.0505, 161.0232, 134.0366	Ferulic acid+Glc	Phenolic acids	P
M23	5.99	C_27_H_26_O_19_	[M−H]^−^	653.1002	653.0990	1.2	301.0355	Quercetin+2GluA	Flavonols	U
M24	6.02	C_16_H_18_O_10_	[M−H]^−^	369.0821	369.0822	−0.1	193.0504	Caffeic acid+CH_2_ + GluA	Phenolic acids	P
M25	6.14	C_9_H_10_O_3_	[M−H]^−^	165.0557	165.0552	0.5	121.0656	p-Coumaric acid+2H	Phenolic acids	P/U
M26	6.21	C_10_H_10_O_4_	[M−H]^−^	193.0504	193.0501	0.3	134.0360	Caffeic acid+CH_2_	Phenolic acids	P/U
M27	6.28	C_21_H_18_O_13_	[M−H]^−^	477.065	477.0669	−1.9	301.0422	Quercetin+GluA	Flavonols	P/U
M28	6.34	C_22_H_20_O_16_S	[M−H]^−^	571.0413	571.0394	1.9	315.0505	Isorhamnetin+SO_3_ + GluA	Flavonols	U
M29	6.62	C_16_H_18_O_10_	[M−H]^−^	369.0826	369.0822	0.4	193.0502	Caffeic acid+CH_2_ + GluA	Phenolic acids	P
M30	6.82	C_21_H_18_O_12_	[M+H]^+^	463.0881	463.0877	0.4	287.0555	Kaempferol+GluA	Flavonols	U
			[M−H]^−^	461.0729	461.0720	0.9	285.0402			
M31	6.85	C_24_H_28_O_11_	[M−H]^−^	491.1562	491.1553	0.9	315.1244, 271.1339	C_18_H_20_O_5_ + GluA	Others	P
M32	6.96	C_24_H_28_O_11_	[M−H]^−^	491.1557	491.1553	0.4	315.1242, 271.1335	C_18_H_20_O_5_ + GluA	Others	P/U
M33	7.15	C_21_H_18_O_12_	[M+H]^+^	463.0879	463.0877	0.2	287.0561, 153.0193	Kaempferol+GluA	Flavonols	P/U
			[M−H]^−^	461.0722	461.0720	0.2	285.0406			
M34	7.17	C_24_H_30_O_10_	[M−H]^−^	477.1763	477.1761	0.2	301.1440	C_18_H_22_O_4_ + GluA	Others	P
M35	7.26	C_26_H_30_O_12_	[M−H]^−^	533.1651	533.1659	−0.8	357.1342	C_20_H_22_O_6_ + GluA	Others	P/U
M36	7.37	C_11_H_12_O_7_S	[M−H]^−^	287.0237	287.0225	1.2	207.0663	Caffeic acid+2CH_2_ + SO_3_	Phenolic acids	P
M37	7.48	C_9_H_10_O_6_S	[M−H]^−^	245.0123	245.0120	0.3	165.0556	p-Coumaric acid+2H + SO_3_	Phenolic acids	P/U
M38	7.49	C_22_H_20_O_13_	[M−H]^−^	491.0826	491.0826	0	315.0507, 300.0250	Isorhamnetin+GluA	Flavonols	P/U
M39	7.56	C_21_H_18_O_12_	[M+H]^+^	463.0876	463.0877	−0.1	287.0558, 165.0197, 153.0193, 121.0298	Kaempferol+GluA	Flavonols	P/U
			[M−H]^−^	461.0718	461.0720	−0.2	285.0402			
M40	7.79	C_22_H_20_O_13_	[M−H]^−^	491.0811	491.0826	−1.5	315.0460	Isorhamnetin+GluA	Flavonols	P/U
M41	7.94	C_21_H_18_O_12_	[M+H]^+^	463.0879	463.0877	0.2	287.0554	Kaempferol+GluA	Flavonols	U
			[M−H]^−^	461.0724	461.0720	0.4	285.0400			
M42	8.03	C_22_H_20_O_13_	[M−H]^−^	491.083	491.0826	0.4	315.0490, 300.0269	Isorhamnetin+GluA	Flavonols	P/U
M43	8.22	C_22_H_20_O_13_	[M−H]^−^	491.0835	491.0826	0.9	315.0488	Isorhamnetin+GluA	Flavonols	U
M44	9.26	C_18_H_18_O_7_S	[M−H]^−^	377.0734	377.0695	3.9	297.1138, 253.1235, 189.0556, 165.0555, 145.0657, 133.0656, 121.0655, 107.0497	C_18_H_18_O_4_ + SO_3_	Others	P/U
M45	9.44	C_18_H_18_O_7_S	[M−H]^−^	377.0731	377.0695	3.6	297.1135, 253.1232, 189.0555, 165.0554, 145.0656, 121.0654, 107.0496	C_18_H_18_O_4_ + SO_3_	Others	P/U
M46	13.72	C_12_H_14_O_4_	[M−H]^−^	221.0821	221.0814	0.7	N/A	Caffeic acid+3CH_2_	Phenolic acids	P/U

P: Plasma; U: Urine.

For example, **M5** exhibited a precursor ion [M−H]^−^ at *m/z* 258.9918 (C_9_H_8_O_7_S, t_R_ = 4.12 min), which was 80 Da (SO_3_) more than caffeic acid (**Fig. S9**). The further fragmentation yielded a prominent ion at *m/z* 179.0344, which was consistent with the removal of an SO_3_ group. Another fragment ion at *m/z* 135.0448 was generated by loss of CO_2_. Based on the combinatorial library and the diagnostic ion library, **M5** was identified as a sulfonated product of caffeic acid. As shown in **Fig. S10**, **M6** showed [M−H]^−^ at *m/z* 273.0076 (C_10_H_10_O_7_S, t_R_ = 4.22 min), 80 Da (SO_3_) more than the quasi-molecular ion of ferulic acid, and produced diagnostic ions at *m/z* 193.0509 [M-H-SO_3_]^−^, 178.0275 [M-H-SO_3_-CH_3_]^−^, 149.0609 [M-H-SO_3_-CO_2_]^−^ and 134.0371 [M-H-SO_3_-CO_2_-CH_2_]^−^. Therefore, **M6** was identified as a sulfonated product of ferulic acid. In the positive ion mode, **M33** (*m/z* 463.0879 [M+H]^+^, C_21_H_18_O_12_) were 176 Da (C_6_H_8_O_6_) higher than the precursor ion of kaempferol. It suggested that M33 was metabolite of kaempferol after glucuronide (**Fig. S11**). In MS1 spectra, the *m/z* 287.0562 resulting from in-source cleavage was determined to be a fragment ion of *m/z* 463.0879 by loss of GluA. The characteristic ions at *m/z* 165.0197, 153.0193 and 121.0298 were observed by RDA cleavage. Therefore, **M33** was identified as kaempferol+GluA in MATLAB.

As shown in Fig. 10, Table 1 and Table 2, based on the core structure and *in vivo* metabolic analysis, *p*-coumaric acid, caffeic acid, ferulic acid, isorhamnetin-7-O-glucoside, kaempferol, quercetin, isorhamnetin, hyperoside and cuscutamine were major absorbed constituents. As depicted in **Fig. S12**, the peak areas of the nine core structures and their derivatives represented 71.22% in the positive ion mode and 84.91% in the negative ion mode of the CS extract. Flavonols (hyperoside, isorhamnetin-7-O-glucoside, isorhamnetin, kaempferol and quercetin) and phenolic acids (*p*-coumaric acid, caffeic acid and ferulic acid) primarily underwent phase II reactions, which included sulfation and glucuronidation. Notably, although caffeic acid and quercetin were not detected as parent compounds *in vivo*, several phase II metabolites of caffeic acid (**M1**, **M5**, **M8**, **M9**, **M13**, **M24**, **M26**, **M29**, **M36**, **M46**) and quercetin (**M19**, **M23** and **M27**) were found. In CS, in contrast to other literature (Liu et al., 2019), the present study identified for the first time a series of alkaloids (**P4**, **P11**, **P12** and **P13**) with high MS response *in vivo* (Fig. 10C). In addition to phenolic acids, the above absorbed 6 components exhibit high MS response in the CS extract (Fig. 10B). However, *p*-coumaric acid (phenolic) demonstrated excellent response in plasma and urine. The above results indicated that *p*-coumaric acid could be produced through the hydrolysis of phenolic acid derivatives.

To reveal the concentrations of the above 9 components *in vivo*, a quantitative method for analyzing them in rat plasma was developed and validated. The method demonstrated satisfactory selectivity (**Fig. S13**), linearity (**Table S7**), recovery, matrix effect (**Table S9**), precision, accuracy (**Table S8** and **Table S10**), and stability (**Table S11**) for all analytes. The results indicated that levels of isorhamnetin-7-O-glucoside, isorhamnetin, caffeic acid, and quercetin were below the lower limit of quantification (LLOQ) in plasma using UPLC-QQQ-MS. The concentrations of the remaining 5 analytes in mixed four points time plasma after oral CS administration were as follows: 291.35 ± 41.41 ng/mL for *p*-coumaric acid, 167.77 ± 11.96 ng/mL for cuscutamine, 7.46 ± 1.93 ng/mL for ferulic acid, 5.10 ± 1.80 ng/mL for kaempferol, and 2.76 ± 1.79 ng/mL for hyperside. The above results indicated that exposure levels of *p*-coumaric acid (phenolic acid) and cuscutamine (alkaloid) in plasma after oral administration of CS were quite substantial (Fig. 10D).

### 3.5 Effects of H_2_O_2_-induced R2C cell injury model on progesterone levels

**Fig. 11:**
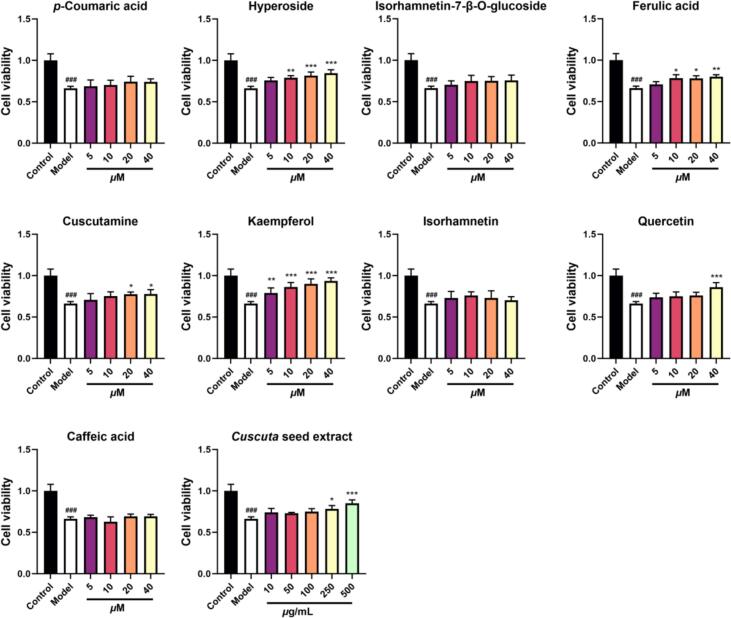
Protection of 9 major absorbed components and Cuscuta seed extract on H_2_O_2_-induced R2C leydig cell. ### *p* < 0.001 *vs*. Control group; * *p* < 0.05, ** *p* < 0.01 *vs*. Model group. Data are represented as MEAN ± SD.

Testicular Leydig cells perform the essential function of synthesizing and secreting steroid hormones, including progesterone and testosterone, which are critical for spermatogenesis. The rat Leydig cell (R2C) represents a valuable *in vitro* model for reproductive research due to its constitutive, high-level progesterone secretion, which occurs independent of hormonal stimulation (Rao et al., 2003). According to metabolic profiles in rat plasma and urine (Fig. 10), the major prototypes were evaluated for improvement of steroid hormone synthesis function on progesterone level and protection in H_2_O_2_-induced R2C leydig cell. The experimental results from MTT showed that CS and 9 compounds were not cytotoxic to R2C cells within 500 μg/mL and 40 μM, respectively (**Fig. S14**). The results showed that when the H_2_O_2_ concentration was 125 μM and the incubation time was 6 h, the survival rate of R2C cells in the model group was almost 60% of the control group. It was showed that CS exhibited the anti-oxidant activity in R2C cells at the concentration of 250–500 *μ*g/mL. The componenets of hyperoside (10–40 μM), ferulic acid (10–40 *μ*M), cuscutamine (20–40 *μ*M), kaempferol (5–40 *μ*M) and quercetin (40 *μ*M) exhibited strong protective effects on H_2_O_2_-induced R2C leydig cell (*p* < 0.05) (Fig. 11).

A quantitative method for the analysis of the progesterone in R2C leydig cell culture medium was developed and validated. The method demonstrated satisfactory selectivity (**Fig. S15**), linearity (**Table S12**), recovery, matrix effect (**Table S13**), precision, accuracy (**Table S14**), and stability (**Table S15**) for progesterone.

**Fig. 12:**
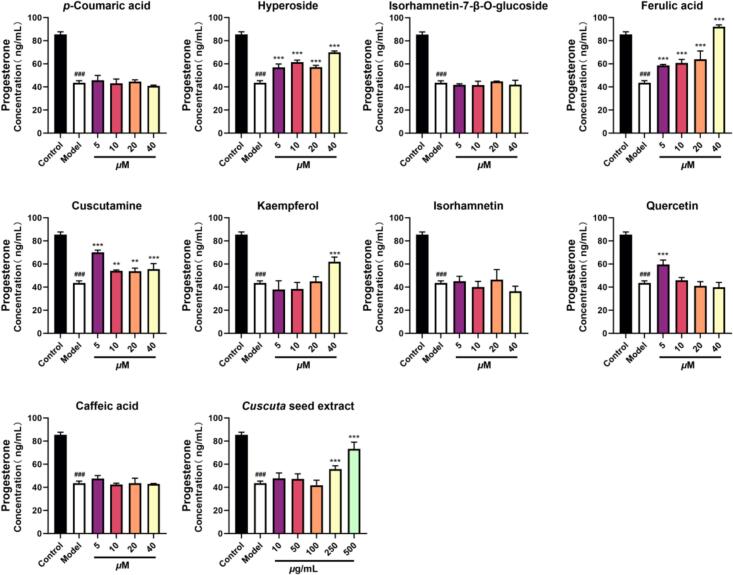
Effects of 9 major absorbed components and *Cuscuta seed* extract on progesterone production in H_2_O_2_-induced R2C leydig cell (n = 5). ### *p* < 0.001 *vs*. Control group; ** *p* < 0.01, ** *p* < 0.001 *vs*. Model group. Data are represented as MEAN ± SD.

As shown in Fig. 12, the level of progesterone was increased when cells were treated with CS in concentration range of 250 μg/mL to 500 *μ*g/mL. At the same time, the results indicated that hyperoside (5–40 *μ*M), ferulic acid (5–40 *μ*M), cuscutamine (5–40 *μ*M), kaempferol (40 *μ*M) and quercetin (5 *μ*M) could increase progesterone level on H_2_O_2_-induced R2C leydig cell (*p* < 0.05). Interestingly, hyperoside, ferulic acid, cuscutamine and quercetin restored progesterone levels function already in the oxidatively damaged state of R2C cells.

## 4 Discussion

Steroid-producing cells are particularly susceptible to oxidative stress because the mitochondrial P450 system is essential for steroid biosynthesis, and these enzymes readily generate reactive oxygen species (ROS) during electron transport (Hanukoglu, 2006; Vitale et al., 2013). Therefore, oxidative damage is a key factor in impaired steroidogenesis, closely linked to the excessive production of ROS and an imbalance in the antioxidant defense system (Vitale et al., 2013). Previous studies (Basque et al., 2021; M. Li et al., 2020; T. Li et al., 2023; Nie et al., 2019; Ruan et al., 2023) and network pharmacology analysis (**Fig. S16**) indicated that hyperoside, ferulic acid, and quercetin could mitigate H₂O₂-induced progesterone decline by simultaneously modulating steroidogenic pathways and oxidative stress. These compounds directly scavenge ROS, mitigating oxidative damage to cellular components critical for steroidogenesis, as evidenced by reduced apoptosis in quercetin-treated granulosa cells (M. Li et al., 2020). Concurrently, they enhance cholesterol availability for progesterone synthesis: Ferulic acid upregulate genes involved in *de novo* cholesterol biosynthesis (LSS, MVK, MVD)(Basque et al., 2021), while quercetin and hyperoside elevate StAR protein expression, facilitating mitochondrial cholesterol transport (M. Li et al., 2020; T. Li et al., 2023; Nie et al., 2019). Further, hyperoside and quercetin boost the enzymatic conversion of cholesterol to progesterone by upregulating CYP11A1 and HSD3B, key enzyme in pregnenolone formation and its metabolism to progesterone (T. Li et al., 2023; Nie et al., 2019). Due to their hydroxy-methoxyphenyl moieties, these phenolic compounds (such as ferulic acid) exert antioxidant effects that preserve cAMP/PKA signaling, thereby maintaining transcriptional activation of steroidogenic genes under oxidative stress (Basque et al., 2021; Huang, Hong, Tan, Xiao, & Gao, 2016). These actions collectively indicated that dietary phenolics could be potent regulators of Leydig cell function through integrated redox protection and hormonal pathway activation. In addition, cuscutamine significantly restored progesterone levels in oxidatively damaged R2C cells in this study. And its electron-rich indole scaffold may contribute to redox protection. Due to the high systemic exposure of cuscutamine observed *in vivo* after the oral administration of CS, this alkaloid appears to be an unrecognized contributor to the bioactivity of CS. This suggests that the reproductive benefits of this functional food may result from the combined effects of phenols and alkaloids. This study confirms the effects of five absorbed compounds in the current cell model and suggests their potential mechanisms based on existing literature and network pharmacology analysis. However, the precise molecular mechanisms of these compounds still need further in-depth investigation and experimental validation in the future.

## 5 Conclusion

This study established an integrated strategy combining a MATLAB-automated MS annotation, an OPLS-DA model, and *in vitro* bioactivity evaluation to comprehensively elucidate the absorbed and bioactive constituents of *Cuscuta seeds* (CS) as a functional food. The MATLAB platform demonstrated superior accuracy over commercial software (UNIFI and SIRIUS) in characterizing 203 compounds in CS. Twenty prototypes and 46 metabolites were identified *in vivo*, revealing 9 major absorbed constituents (*p*-coumaric acid, caffeic acid, ferulic acid, isorhamnetin-7-O-glucoside, kaempferol, quercetin, isorhamnetin, hyperoside, and cuscutamine). Notably, cuscutamine and *p*-coumaric acid showed unexpectedly high systemic exposure. Importantly, hyperoside, ferulic acid, cuscutamine, kaempferol, and quercetin significantly attenuated H₂O₂-induced oxidative damage in R2C Leydig cells and restored progesterone levels. This integrated strategy provides a robust platform for foodomics research and promotes the understanding of CS as a functional food.

## CRediT authorship contribution statement

**Xi-yang Tang:** Writing – review & editing, Writing – original draft, Supervision, Project administration, Methodology, Conceptualization. **Ming-jia Ma:** Visualization, Validation, Software, Investigation. **Meng-le Du:** Software, Investigation. **Lv-qi Xie:** Visualization, Validation. **Jia-jia Chen:** Investigation. **Ze-xi Tan:** Visualization. **Zhi-jian Su:** Investigation. **Zi-qin Dai:** Resources. **Lei Huang:** Supervision, Methodology, Data curation. **Yi Dai:** Supervision, Resources.

## Declaration of competing interest

The authors declare that they have no known competing financial interests or personal relationships that could have appeared to influence the work reported in this paper.

## Data Availability

No data was used for the research described in the article.

## Appendix A Supplementary data

Supplementary material. Image 1
