# Supplementary material for: Unveiling the absorbed bioactive constituents of Cuscuta seeds: A systematic strategy integrating automated MS annotation, in vivo chemometric screening and bioactivity evaluation
Source: Food Chem X. 2026 Jan 20;34:103564. doi: 10.1016/j.fochx.2026.103564 (PMC12860374; doi:10.1016/j.fochx.2026.103564)
Supplement: Supplementary file 1 — Supplementary material. [file mmc1.docx]

**Unveiling the absorbed bioactive constituents of *Cuscuta* seeds: A systematic strategy integrating automated MS annotation, *in vivo* chemometric screening and bioactivity evaluation**

Xi-yang Tang^a^, Ming-jia Ma^a^, Meng-le Du^a^, Lv-qi Xie^a^, Jia-jia Chen^a^, Ze-xi Tan^a^, Zhi-jian Su^c^, Zi-qin Dai^d^, Lei Huang^b,*^, Yi Dai^a,*^

^a^ *State Key Laboratory of Bioactive Molecules and Druggability Assessment, Guangdong Basic Research Center of Excellence for Natural Bioactive Molecules and Discovery of Innovative Drugs, Institute of Traditional Chinese Medicine & Natural Products, College of Pharmacy, Guangdong Province Key Laboratory of Pharmacodynamic Constituents of TCM and New Drugs Research, and International Cooperative Laboratory of Traditional Chinese Medicine Modernization and Innovative Drug Development of Ministry of Education (MOE) of China, Jinan University, Guangzhou 510632, PR China.*

*^b^ Department of Pharmacy, First People’s Hospital of Yancheng, Yancheng 224006, PR China*

*^c^ Department of Food Science and Engineering, Department of Developmental and Regenerative Biology, Biopharmaceutical R&D Center, Jinan University, Guangzhou 510632, PR China*

*^d^ Guangzhou huibiao testing technology center, Guangzhou 510632, PR China.*

* *Correspondence to: Lei Huang, Department of Pharmacy, First People’s Hospital of Yancheng, China. E-mail: 18771073067@163.com.*

* *Correspondence to: Prof. Dr. Yi Dai, Institute of Traditional Chinese Medicine and Natural Products, College of Pharmacy, Jinan University, China. Tel: +86 20 85220785; fax: +86 20 85221559 E-mail: daiyi1004@163.com.*

## **Legend to figures and tables**

**Fig. S1.** Reported chemical structure of *Cuscuta* seeds (CS)

**Fig. S2.** The parent molecules and group fragments of (A) flavonols, (B) lignans, (C) phenolic acids, (D) alkaloids, (E) resin glycosides and (F) phospholipids

**Fig. S3.** The characteristic fragments and neutral losses of compound **46** in MS2 spectra.

**Fig. S4.** The characteristic fragments and neutral losses of compound **81** in MS2 spectra.

**Fig. S5.** The characteristic fragments and neutral losses of compound **112** in MS2 spectra.

**Fig. S6.** The MS1 and MS2 spectra of compound **65** (hyperoside) in the positive ion mode

**Fig. S7.** The MS1 and MS2 spectra of compound **25** (Chlorogenic acid) in the positive ion mode

**Fig. S8.** The MS1 and MS2 spectra of compound **98** (carboxyl-cuscutamine) in the negative ion mode

**Fig. S9.** The MS1 and MS2 spectra of **M5** (Caffeic acid+SO_3_) in the negative ion mode

**Fig. S10.** The MS1 and MS2 spectra of **M6** (Ferulic acid+SO_3_) in the negative ion mode

**Fig. S11.** The MS1 and MS2 spectra of **M33** (Kaempferol+GluA) in the positive ion mode

**Fig, S12.** The percentage of the nine compounds and their derivatives in *Cuscuta seed* extract

**Fig. S13.** Typical MRM chromatogram of 9 analytes: (A) Blank plasma. (B) Blank plasma spiked with mixed standard solution at LLOQ. (C) Mixed four points time plasma after oral administration of CS. (1) Isorhamnetin-7-β-O-glucoside; (2) Hyperoside; (3) Quercetin; (4) Kaempferol; (5) Cuscutamine; (6) Isorhamnetin; (7) Ferulic acid; (8) Caffeic acid; (9) p-Coumaric acid.

**Fig. S14.** The effect of 9 major absorbed components and Cuscuta seed extract on cell viability in R2C leydig cell.

**Fig. S15.** Typical MRM chromatogram of progesterone: (A) Blank culture medium. (B) Blank culture medium spiked with progesterone at LLOQ. (C) The R2C leydig cell culture medium

**Fig. S16.** (A) Venn diagram; (B) PPIs of overlapped targets between 5 absorbed bioactive compounds and oxidative damage induced steroidogenic disorder; (C) KEGG pathway enrichment analysis of overlapped targets between 5 absorbed bioactive compounds and oxidative damage induced steroidogenic disorder; (D) Component–target network of 5 absorbed bioactive compounds.

**Fig. S17**. The MS1 and MS2 spectra of compound **200** (LPC (16:0)) in the negative ion mode

**Table S1.** Optimized MRM conditions of 9 analytes

**Table S2.** Detailed information for the in-house reported library of *Cuscuta* seeds

**Table S3.** Characterization of chemical constituents of *Cuscuta* seeds

**Table S4.** Prediction accuracy of compounds by different software

**Table S5.** The nodes and edges of various structural types with different α and β values in positive ion

**Table S6.** The nodes and edges of various structural types with different α and β values in negative ion.

**Table S7.** The regression equations and linear ranges of 9 analytes (n = 3) in rat plasma

**Table S8.** Precision and accuracy of 9 analytes at LLOQ in rat plasma (n=5)

**Table S9.** Recoveries and matrix effects of 9 analytes (n=5) in rat plasma

**Table S10.** Intra-day and inter-day precision and accuracy of 9 analytes at three concentration (LQC, MQC, HQC) in rat plasma

**Table S11. S**hort-term stability, freeze-thaw stability and auto-sampler stability of 9 analytes in rat plasma (n=5)

**Table S12.** The regression equation (n = 3), linear range and LLOQ (n = 5) of progesterone in cell culture medium

**Table S13.** Recoveries and matrix effects of progesterone (n = 5) in cell culture medium

**Table S14.** Intra-day and inter-day precision and accuracy of progesterone at three concentration (LQC, MQC, HQC) in cell culture medium

**Table S15. S**hort-term stability, long-term stability, freeze-thaw stability and auto-sampler stability of progesterone in cell culture medium (n=5)

### S1 The standard solution preparation and UPLC-QQQ-MS conditions for quantitative progesterone levels

**S1.1. The standard solution preparation**

Progesterone and progesterone-d_9_ (internal standards, IS) were separately dissolved in methanol (10 mL). And the concentrations of all analyte stock solutions were 1 mg/mL. The stock solutions were stored at -80 °C and were brought to room temperature before use.

Calibration curve samples were prepared by adding 50 *μ*L of IS solution (1.25 ng/mL) and 50 *μ*L of progesterone solution into 100 *μ*L culture medium. Eight different concentrations were used to build calibration curves for progesterone. The prepared calibration standards of progesterone were set in the range of 1-128 ng/mL. Quality control (QC) samples were prepared at the concentration of 2, 16 and 64 ng/mL.

**S1.2. Sample preparation**

One hundred microliter of each R2C leydig cell culture medium was taken in a 2 mL centrifuge tube. Firstly, cell culture medium samples were added with 50 *μ*L methanol and 50 *μ*L IS, and then mixed for 30 s on a vortex-mixer. After that, 0.6 mL methyl-tert-butyl ether (MTBE) was added, and the samples were vortexed for 2 min. The upper organic phase was separated and then evaporated under nitrogen. Secondly, the dried residue was redissolved in solution of hydroxylamine hydrochloride (100 *μ*L, 100 mM), and then incubated at 60 °C for 30 min. After that, 0.6 mL MTBE was added, and the samples were vortexed for 2 min. The upper organic phase was separated and then evaporated under nitrogen. Finally, the dried residue was redissolved in methanol and then submitted for UPLC-QQQ-MS analysis.

**S1.3. UPLC-QQQ-MS conditions**

Separation was performed using the ACQUITY™ UPLC I-Class system on an Acquity UPLC BEH C18 column (2.1 mm × 100 mm, 1.7 *μ*m). The mobile phases consisted of 0.1 % (v/v) formic acid (phase A) and 0.1 % (v/v) formic acid in acetonitrile (phase B). The gradient conditions were as follows: 0-0.5 min, 2 %B; 0.5-2.5 min, 2 %-35 %B; 2.5-4.0 min, 35 %-80 %B; 4.0−7.0 min, 80 %-90 %B; 7.0-8.0 min, 90%-100%B; 8.0-9.0 min, isocratic elution with 100 % B for 1.0 min, and then returned to the initial condition. The total chromatographic run time was 10 min. The chromatographic separation was performed at the flow rate of 0.3 mL/min and the column temperature was set at 35°C. The UPLC system injection was set to 2 *μ*L volume. The autosampler was conditioned at 15°C.

A Xevo TQ-S micro triple quadrupole mass spectrometer (Waters Corp., Milford, MA, USA) equipped with a positive ESI mode was used for the detection of all analytes. In the Xevo TQ-XS mass spectrometer, MRM mode was operated for quantification of progesterone. The MS conditions were set as follows: capillary voltage 3.0 kV (ESI^+^), source temperature 150°C, and cone gas 150 L/h. The desolvation gas was heated to temperature 550°C and delivered at a flow rate of 1000 L/h. Quantification was performed using multiple reaction monitoring (MRM) mode of two suitable transition pairs of progesterone (345.25/124.08, Cone voltage: 30V, Collision energy: 30eV) and IS/progesterone-d_9_ (354.31/128.10, Cone voltage: 30V, Collision energy: 30eV).

### S2 Network Pharmacology

Firstly, 5 absorbed bioactive compounds (hyperoside, ferulic acid, cuscutamine, kaempferol, and quercetin) were transformed into canonical SMILES through the PubChem (<https://pubchem.ncbi.nlm.nih.gov/>). Then, the canonical SMILES information was uploaded into the Swiss Target Prediction database (<http://www.swisstargetprediction.ch/>) in ‘‘homo sapiens” species to predict all potential targets of compounds. Secondly, the keyword about “oxidative damage induced steroidogenic disorder” was searched to obtain oxidative damage induced steroidogenic disorder related gene targets from the GeneCards database (<https://www.genecards.org/>) and Man database (OMIM <http://omim.org/>). Thirdly, 5 compounds targets and the disease targets were imported in bioinformatics website (<https://bioinfogp.cnb.csic.es/tools/venny/>) to draw a Venn diagram and obtain their common gene targets. Afterwards, the overlapping targets between the compounds targets and the oxidative damage induced steroidogenic disorder targets were inputted into STRING database (<http://string-db.org/>) to construct the protein-protein interaction (PPI) network. The Cytoscape 3.9.1 was used to visualize a PPI network diagram and screen key targets according to the network node topological parameter ‘‘degree, closeness, and betweenness”. Then, 5 absorbed bioactive compounds of CS and its therapeutic key targets in oxidative damage induced steroidogenic disorder were introduced into Cytoscape 3.9.1 to construct the compound-target network. Finally, the DAVID database (https://davidbioinformatics.nih.gov/) was used for KEGG pathway analysis.

As shown in **Fig. S16A** and **S16B**, 75 overlapped targets between 5 compounds and oxidative damage induced steroidogenic disorder were found, and among them, CYP11A1, HSD3B1, ESR1, STAT3, AKT1, CYP19A1, ESR2, CTNNB1, CYP1A1, AKR1C3, NFE2L2, PRKACA, PTGS2, RELA, REN, SRC, ACE, TNF, and EGFR were considered to be the key targets of 5 compounds against oxidative damage induced steroidogenic disorder (Degree ≥ 17, Closeness ≥ 0.006995, betweenness ≥ 73.5467). The composition–target network diagram of 5 absorbed bioactive compounds is shown in **Fig. S16D**, and a total of 75 targets were hit. A total of 75 overlapped targets were imported into the DAVID database for pathway enrichment analysis, the top 20 pathways were selected in **Fig. 16C** according to P value for plotting. The results showed that pathways including steroid hormone biosynthesis, chemical carcinogenesis - reactive oxygen species (ROS), estrogen signaling pathway, relaxin signaling pathway were the potential pathways of 5 compounds against oxidative damage induced steroidogenic disorder.

### S3 Analysis of compound 200 in negative ion mode

As shown in **Fig. S17**, the *m/z* 480.3102 resulting from in-source cleavage was determined to be a fragment ion of *m/z* 540.3311 by loss of formic acid and methyl. Therefore, the quasimolecular ion [M+HCOO]^-^ of compound **200** was *m/z* 540.3311 (C_24_H_50_NO_7_P). Based on MATLAB automated MS data analysis platform and comparing with reference standards, it was identified as a phospholipid, LPC (16:0).


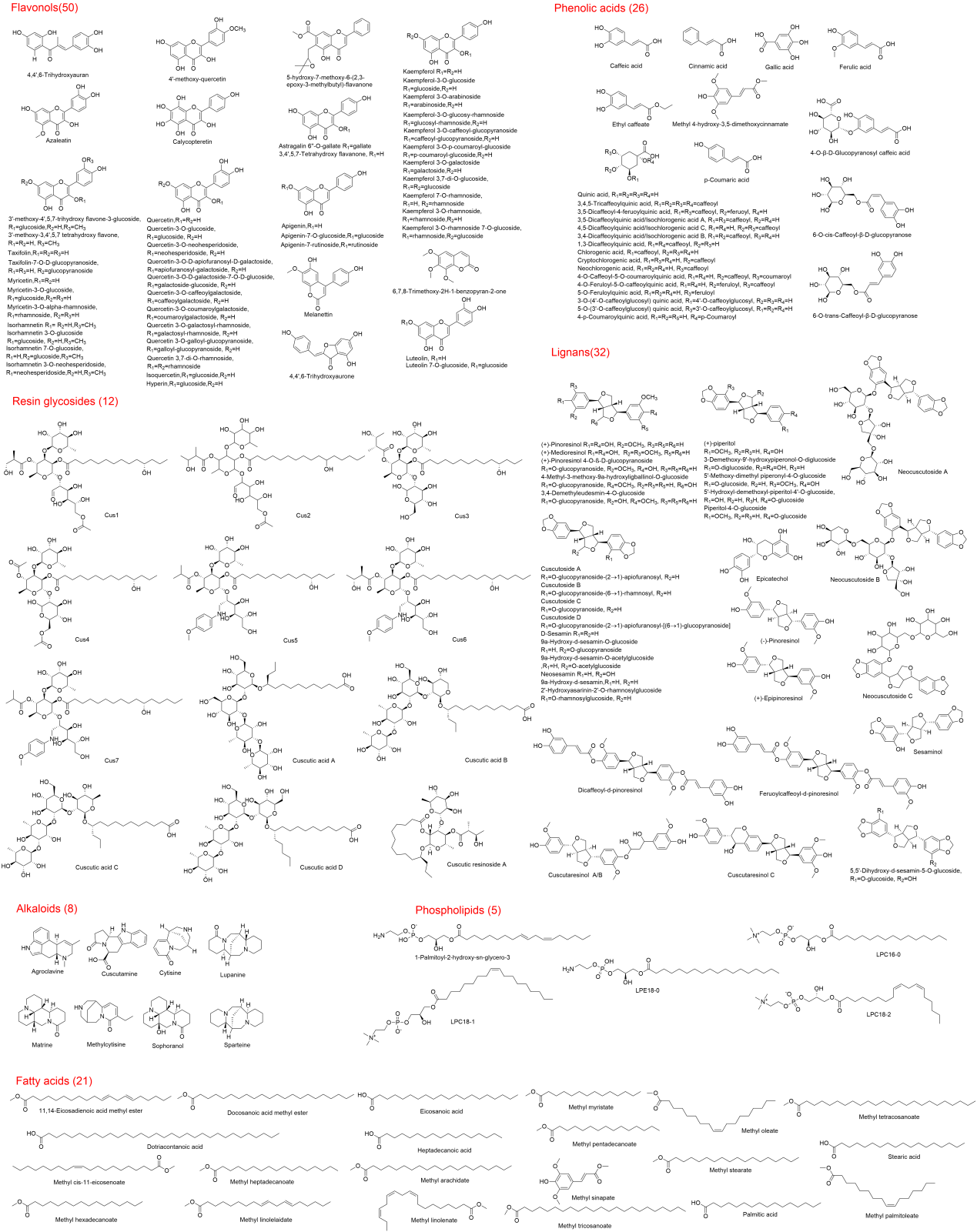


**Fig. S1.** Reported chemical structure of *Cuscuta* seeds (CS)


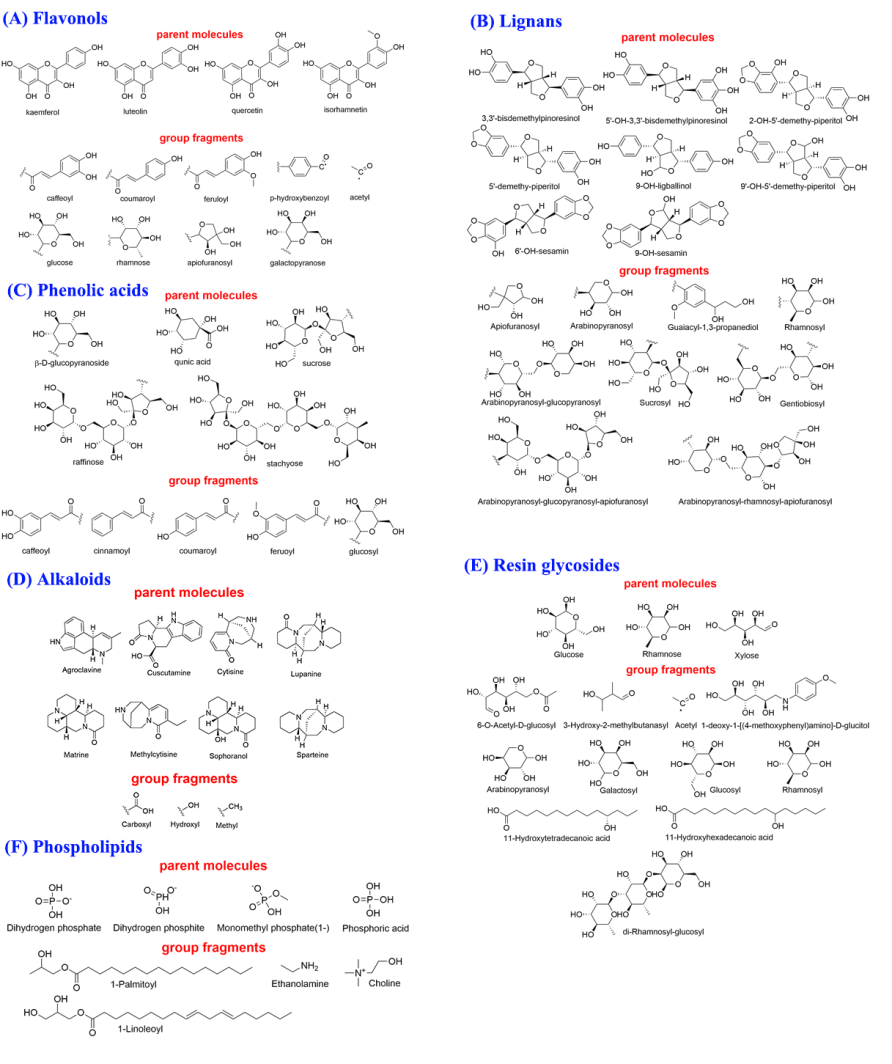


**Fig. S2.** The parent molecules and group fragments of (A) flavonols, (B) lignans, (C) phenolic acids, (D) alkaloids, (E) resin glycosides and (F) phospholipids


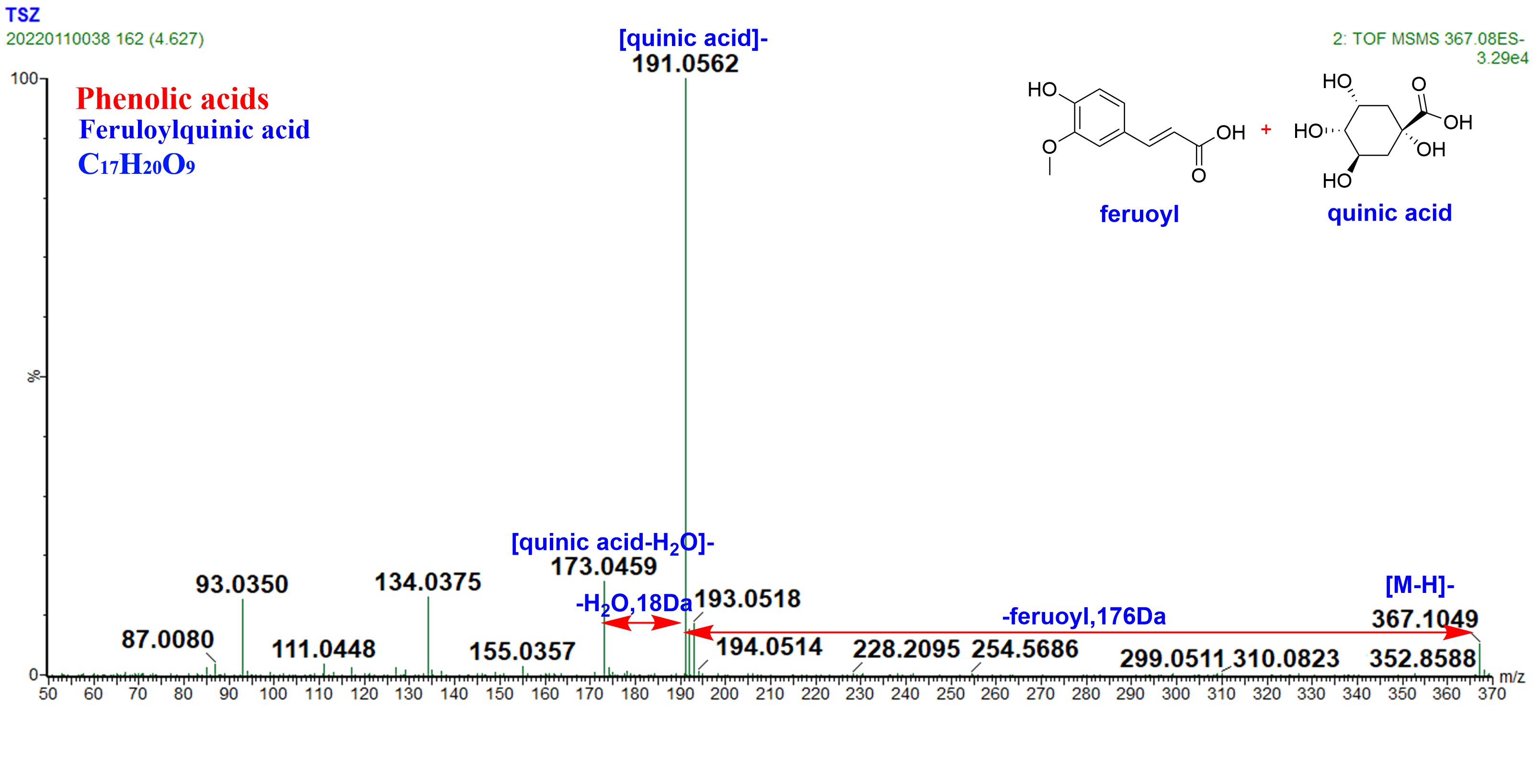


**Fig. S3.** The characteristic fragments and neutral losses of compound **46** in MS2 spectra.

**
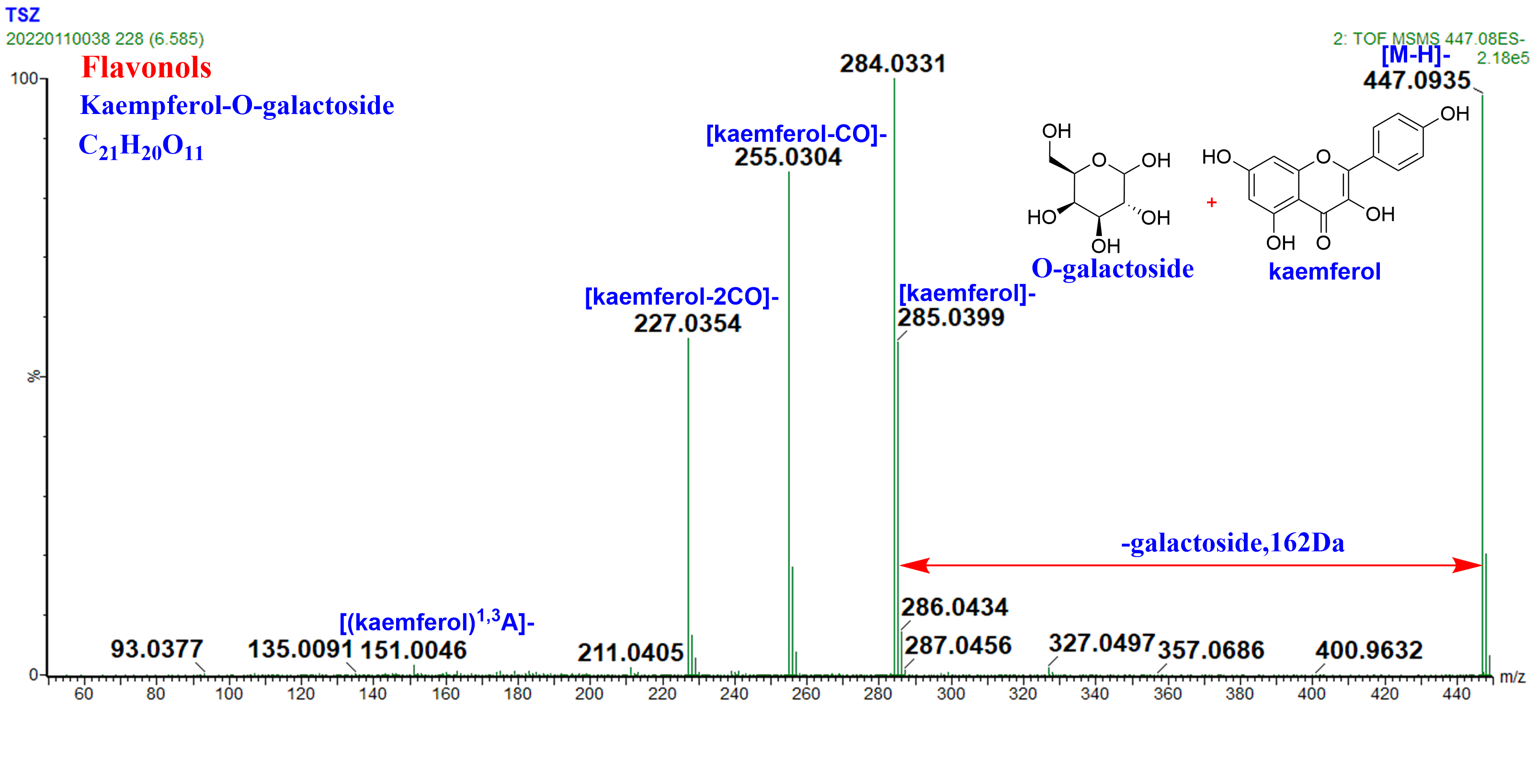
**

**Fig. S4.** The characteristic fragments and neutral losses of compound **81** in MS2 spectra.


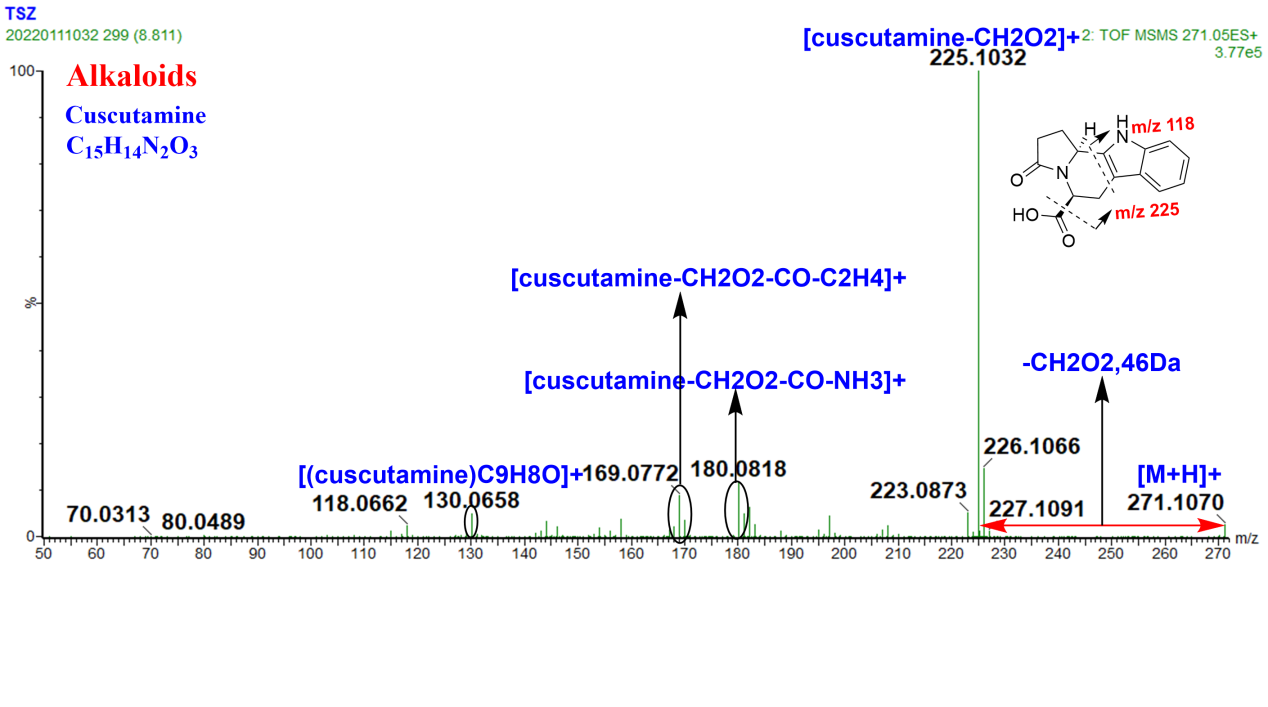


**Fig. S5.** The characteristic fragments and neutral losses of compound **112** in MS2 spectra.

**
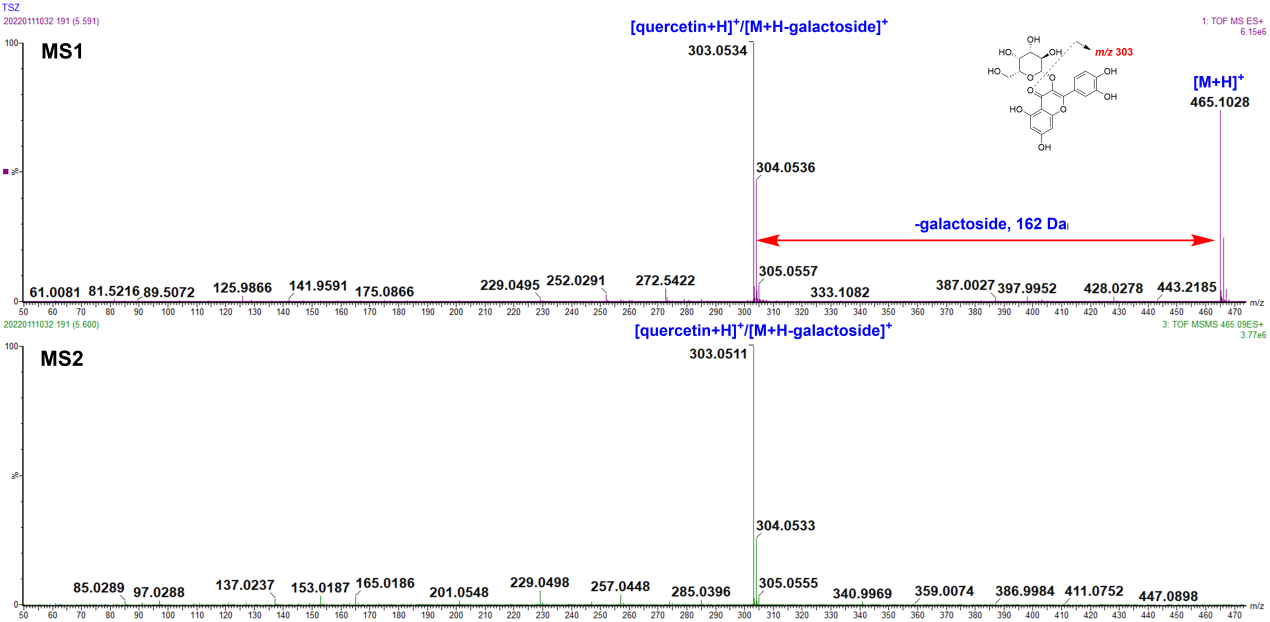
**

**Fig. S6.** The MS1 and MS2 spectra of compound **65** (hyperoside) in the positive ion mode


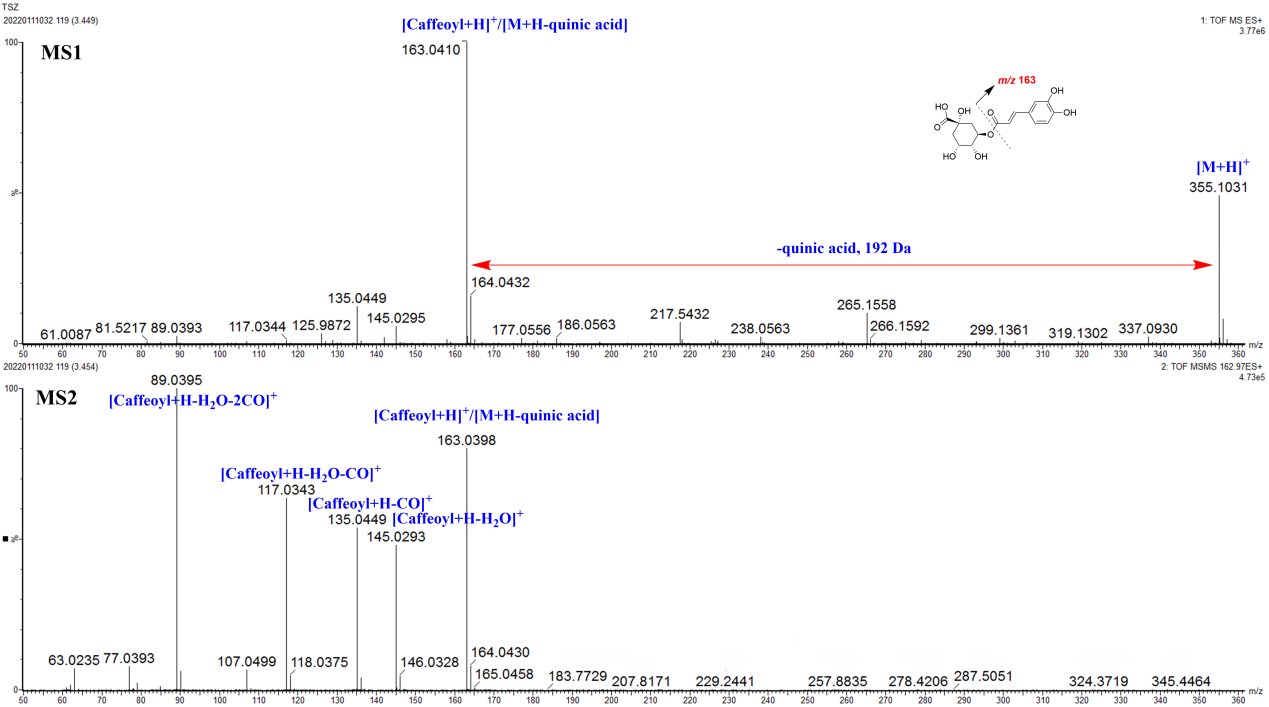
 **Fig. S7.** The MS1 and MS2 spectra of compound **25** (Chlorogenic acid) in the positive ion mode

**
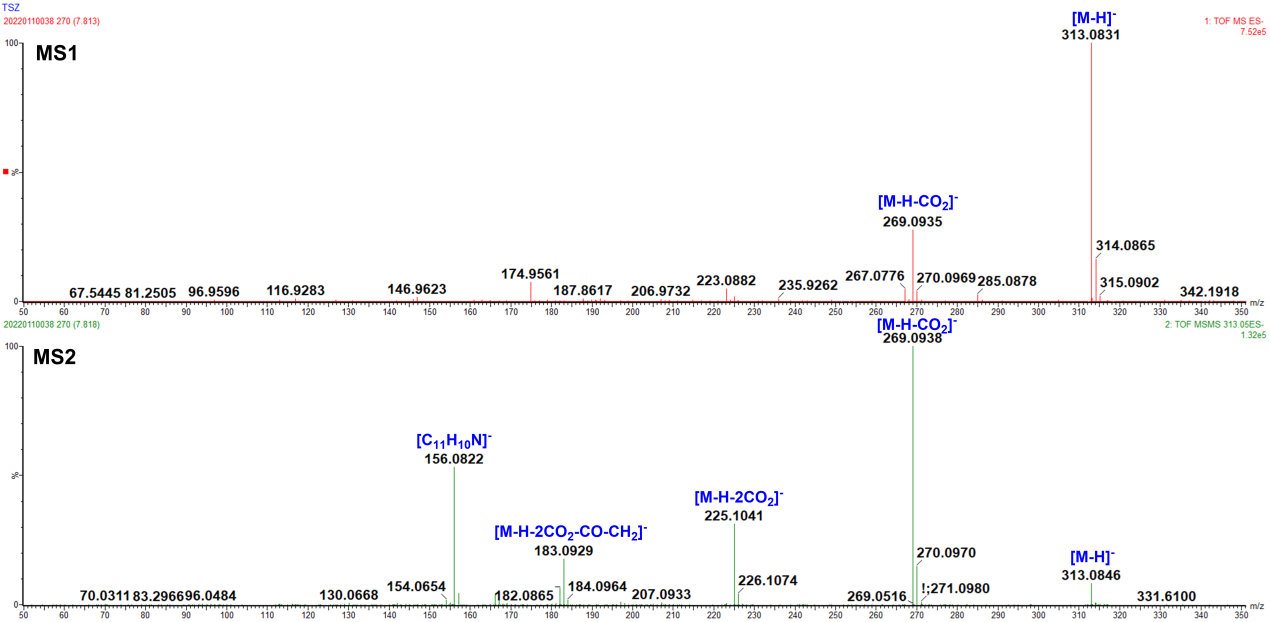
**

**Fig. S8.** The MS1 and MS2 spectra of compound **98** (carboxyl-cuscutamine) in the negative ion mode

**
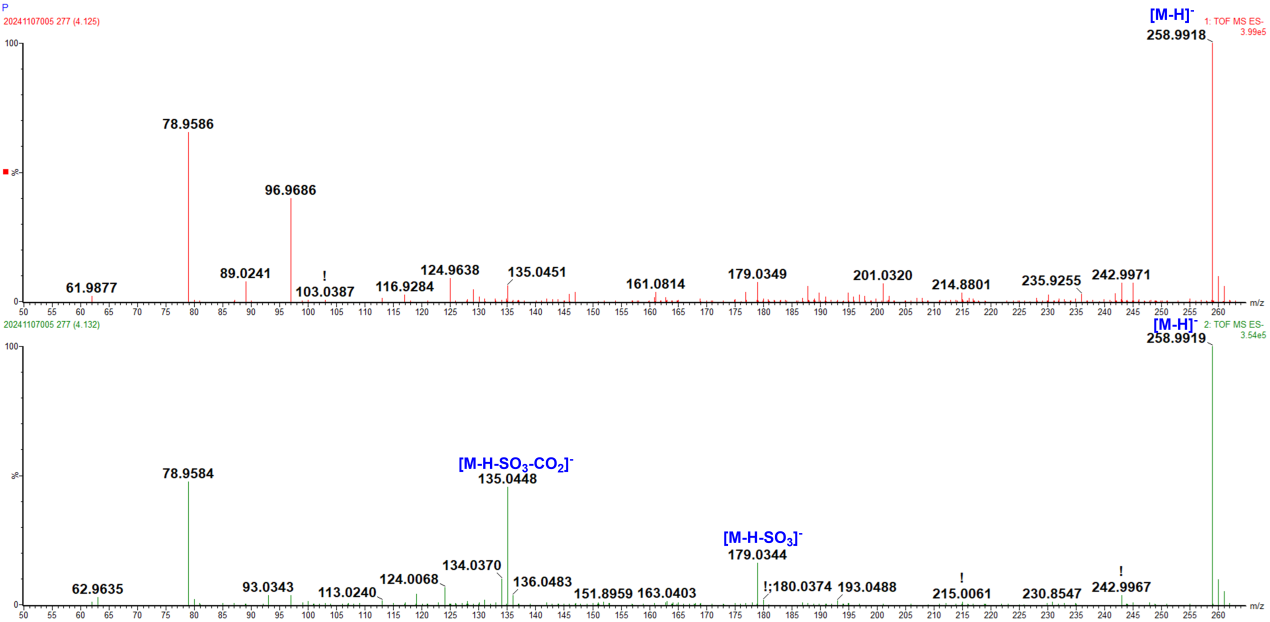
**

**Fig. S9.** The MS1 and MS2 spectra of **M5** (Caffeic acid+SO_3_) in the negative ion mode

**
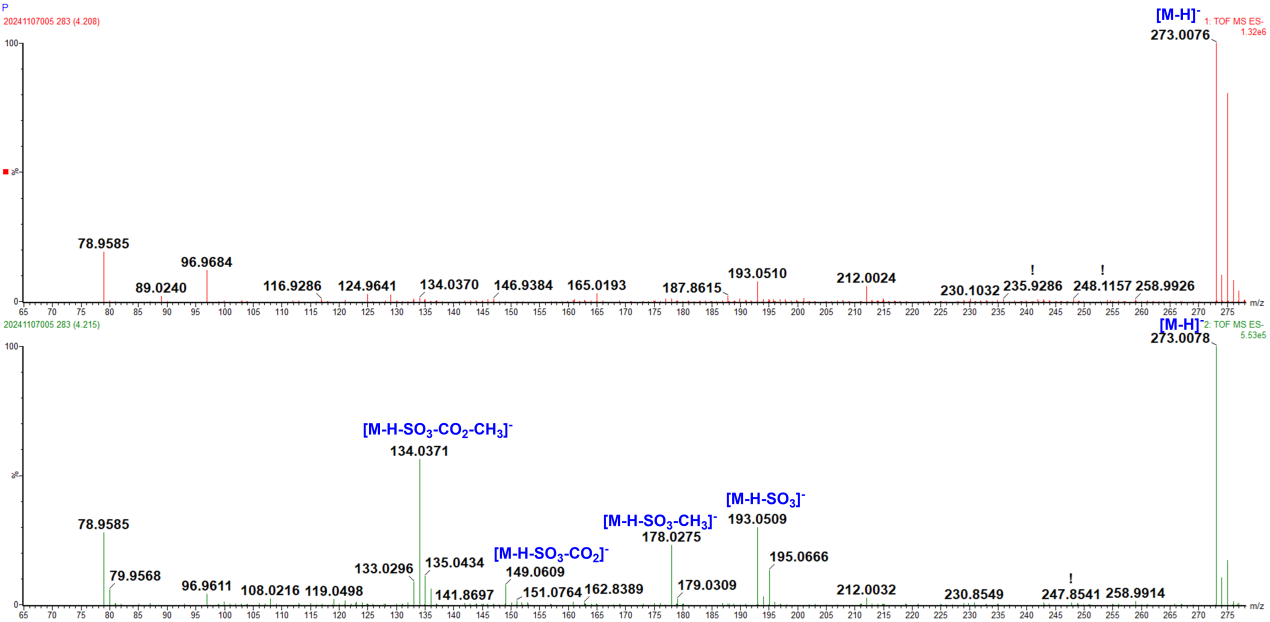
**

**Fig. S10.** The MS1 and MS2 spectra of **M6** (Ferulic acid+SO_3_) in the negative ion mode

**
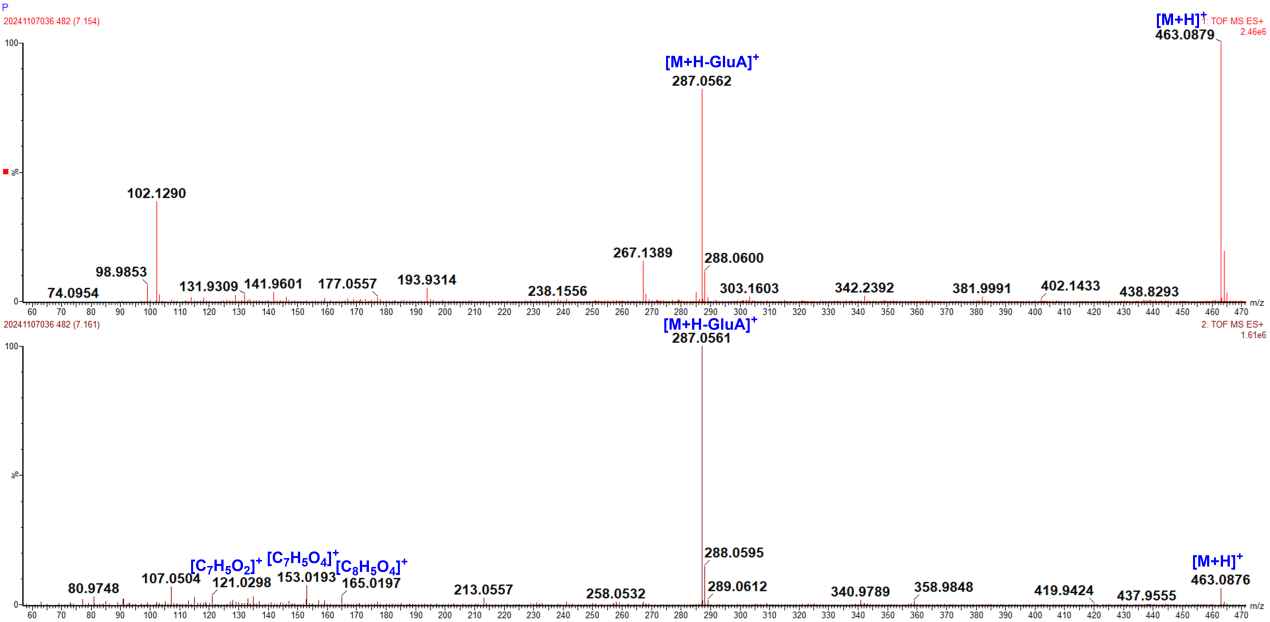
** **Fig. S11.** The MS1 and MS2 spectra of **M33** (Kaempferol+GluA) in the positive ion mode


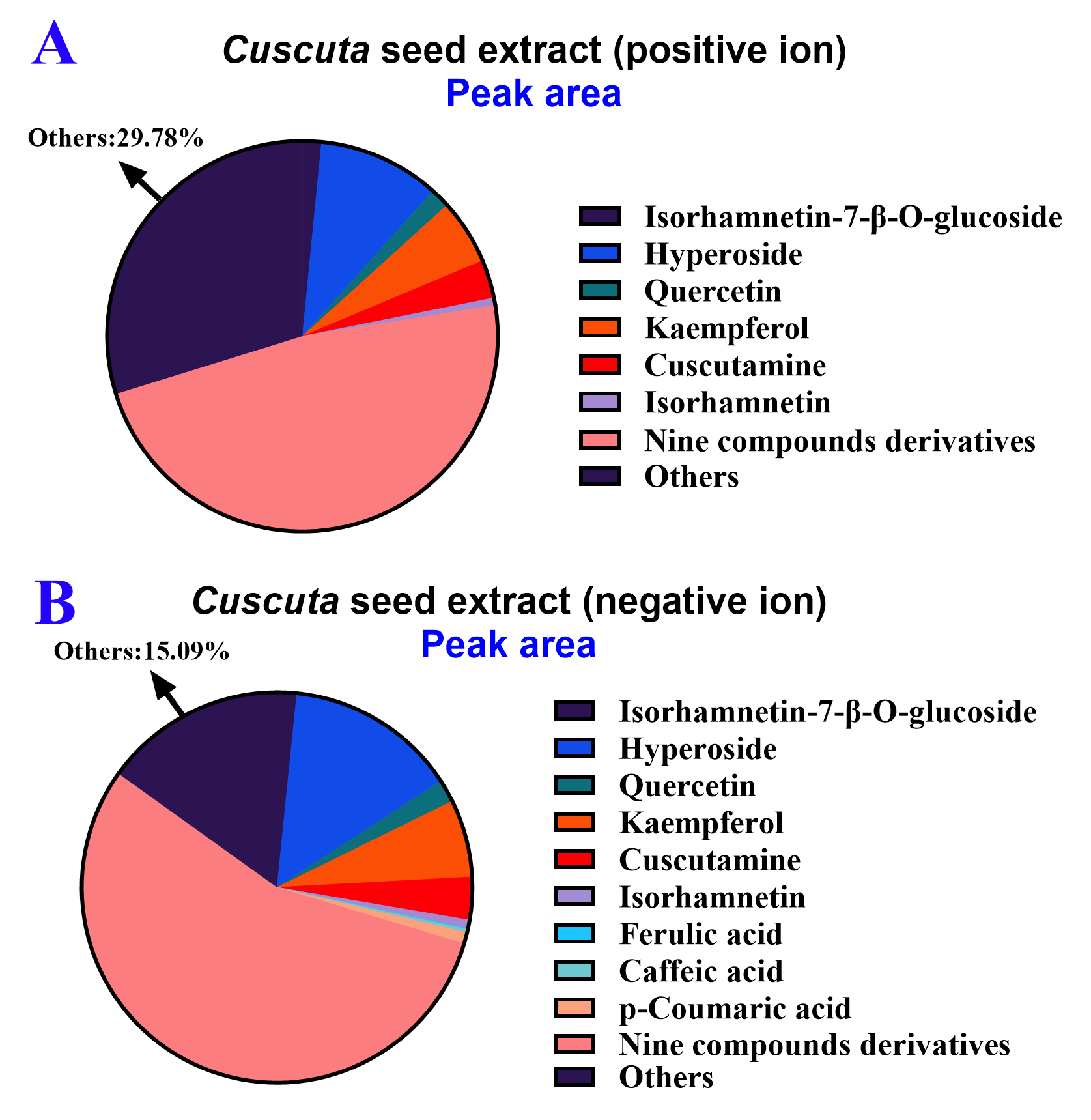


**Fig. S12.** The percentage of the nine compounds and their derivatives in *Cuscuta* seed extract

**
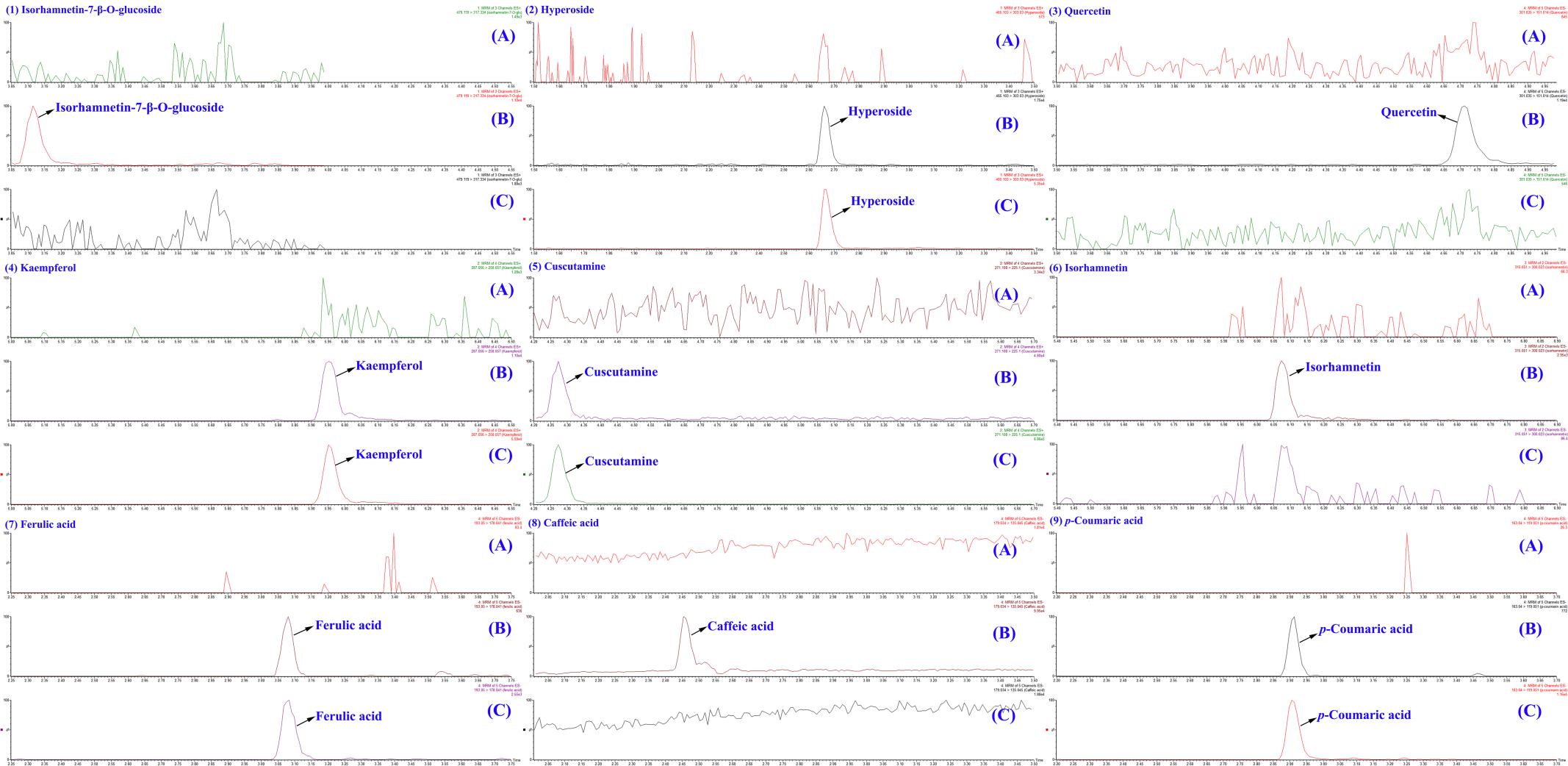
**

**Fig. S13.** Typical MRM chromatogram of 9 analytes: (A) Blank plasma. (B) Blank plasma spiked with mixed standard solution at LLOQ. (C) Mixed four points time plasma after oral administration of CS. (1) Isorhamnetin-7-β-O-glucoside; (2) Hyperoside; (3) Quercetin; (4) Kaempferol; (5) Cuscutamine; (6) Isorhamnetin; (7) Ferulic acid; (8) Caffeic acid; (9) p-Coumaric acid.


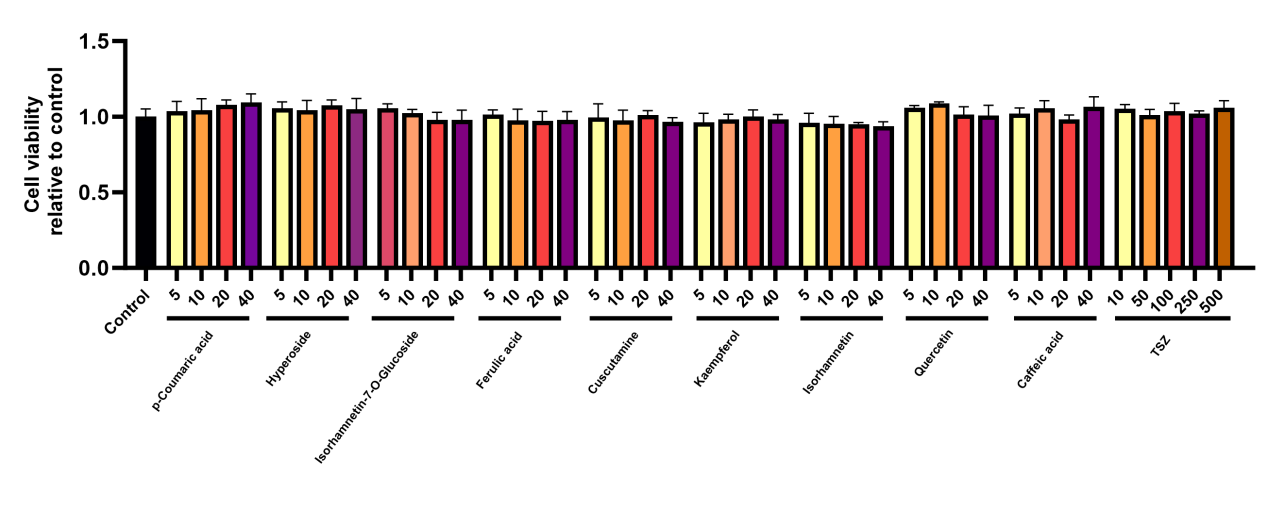


**Fig. S14.** The effect of 9 major absorbed components and *Cuscuta* seed extract on cell viability in R2C leydig cell.**
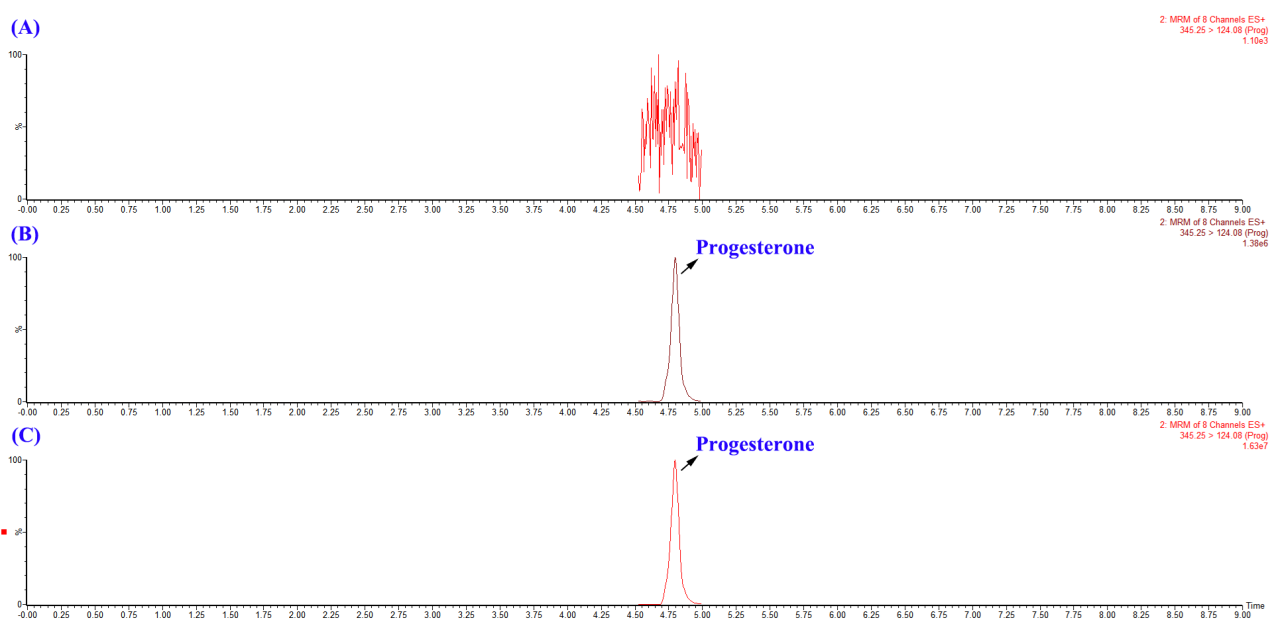
**

**Fig. S15.** Typical MRM chromatogram of progesterone: (A) Blank culture medium. (B) Blank culture medium spiked with progesterone at LLOQ. (C) The R2C leydig cell culture medium

**
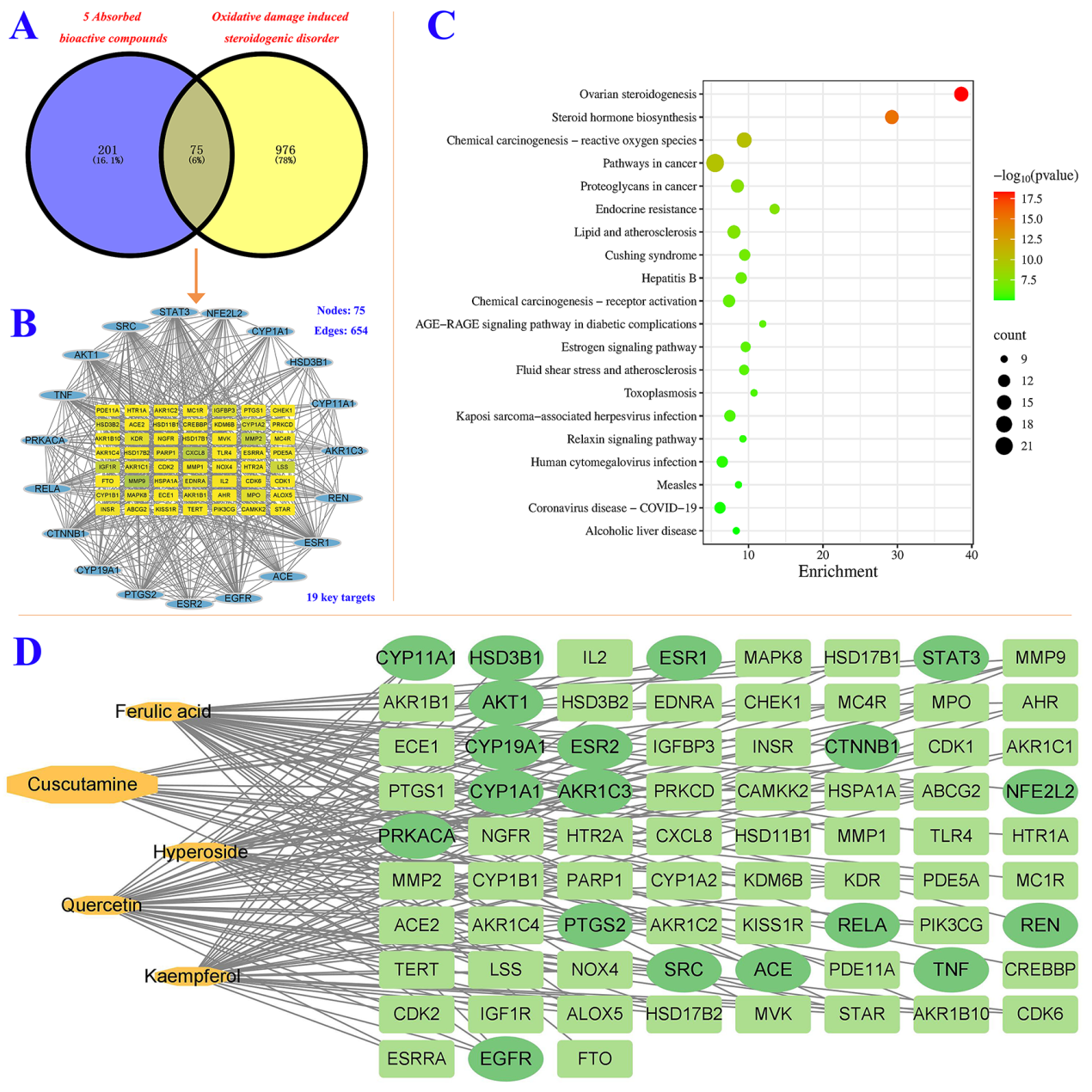
Fig. S16.** (A) Venn diagram; (B) PPIs of overlapped targets between 5 absorbed bioactive compounds and oxidative damage induced steroidogenic disorder; (C) KEGG pathway enrichment analysis of overlapped targets between 5 absorbed bioactive compounds and oxidative damage induced steroidogenic disorder; (D) Component–target network of 5 absorbed bioactive compounds.


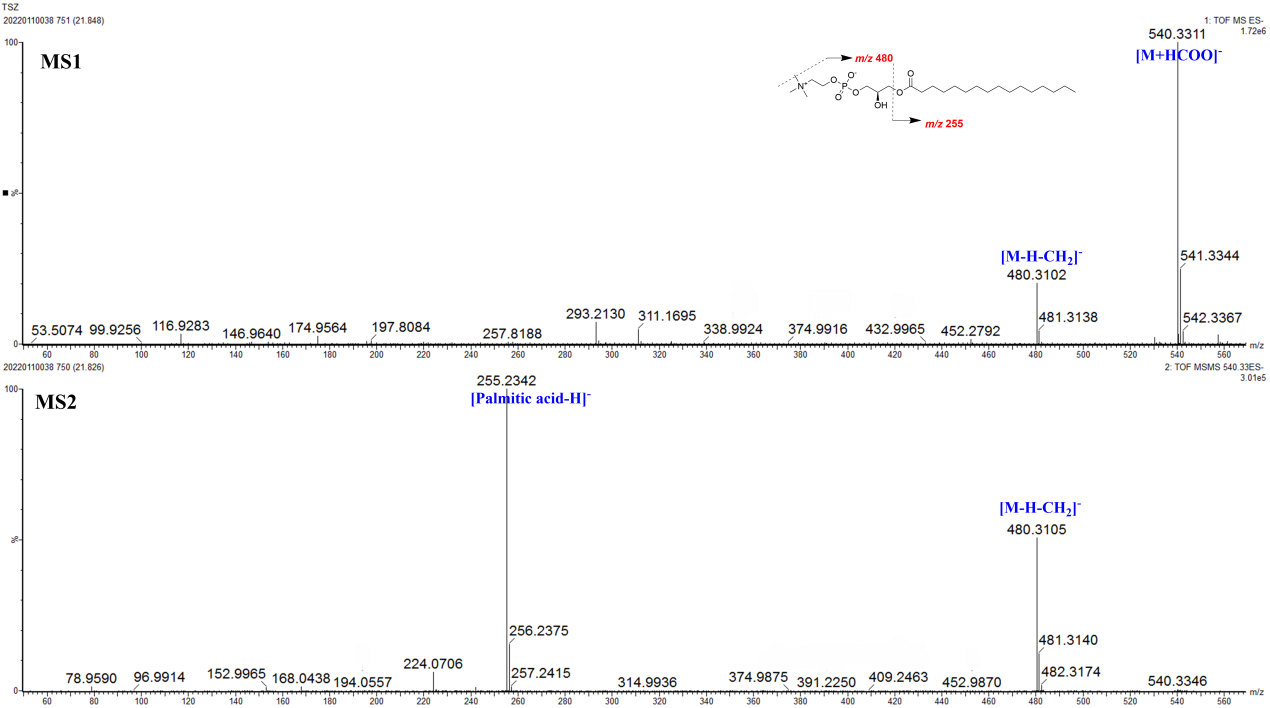


**Fig. S17**. The MS1 and MS2 spectra of compound **200** (LPC (16:0)) in the negative ion mode

**Table S1.** Optimized MRM conditions of 9 analytes

| Compounds | Selected ion | Parent ions (m/z) | Daughter ions (m/z) | Cone voltage (V) | Collision energy  (eV) |
| --- | --- | --- | --- | --- | --- |
| Isorhamnetin-7-β-O-glucoside | [M+H]^+^ | 479.12 | 317.33 | 20 | 20 |
| Hyperoside | [M+H]^+^ | 465.10 | 303.03 | 21 | 15 |
| Quercetin | [M+H]^+^ | 303.05 | 257.05 | 15 | 25 |
| Kaempferol | [M+H]^+^ | 287.06 | 258.06 | 15 | 25 |
| Cuscutamine | [M+H]^+^ | 271.11 | 225.10 | 25 | 15 |
| Isorhamnetin | [M-H]^-^ | 315.05 | 300.02 | 15 | 20 |
| Ferulic acid | [M-H]^-^ | 193.05 | 178.04 | 30 | 20 |
| Caffeic acid | [M-H]^-^ | 179.03 | 135.04 | 30 | 15 |
| *p*-Coumaric acid | [M-H]^-^ | 163.04 | 119.05 | 30 | 40 |
| Icariin (IS) | [M+H]^+^ | 677.24 | 369.13 | 20 | 25 |
| Icariin (IS) | [M+HCOO]^-^ | 721.23 | 513.18 | 15 | 15 |

**Table S2.** Detailed information for the in-house reported library of *Cuscuta* seeds

| No. | Compound name | Formula | Molecular weight | Structure | Reference |
| --- | --- | --- | --- | --- | --- |
| 1 | Quercetin | C_15_H_10_O_7_ | 302.0427 | Flavonols | [1] |
| 2 | Hyperoside | C_21_H_20_O_12_ | 464.0955 | Flavonols | [1] |
| 3 | Kaempferol | C_21_H_20_O_12_ | 286.0477 | Flavonols | [1] |
| 4 | Myricetin | C_15_H_10_O_8_ | 318.0376 | Flavonols | [1] |
| 5 | Quercetin-3-O-neohesperidoside | C_21_H_30_O_16_ | 610.1534 | Flavonols | [2] |
| 6 | Kaempferol-3-O-glucoside | C_21_H_18_O_12_ | 462.0798 | Flavonols | [3] |
| 7 | Leuteolin | C_15_H_10_O_6_ | 286.0477 | Flavonols | [2] |
| 8 | Quercetin-3-O-glucoside | C_21_H_20_O_12_ | 464.0955 | Flavonols | [2] |
| 9 | Apigenin-7-rutinoside | C_27_H_30_O_14_ | 578.1636 | Flavonols | [4] |
| 10 | 3'-Methoxy-3,4',5,7 tetrahydroxy flavone | C_16_H_14_O_7_ | 302.079 | Flavonols | [5] |
| 11 | 3'-Methoxy-4',5,7-trihydroxy flavone-3-glucoside | C_15_H_10_O_6_ | 288.0634 | Flavonols | [5] |
| 12 | Myricetin-3-glucoside | C_21_H_20_O_13_ | 480.0904 | Flavonols | [6] |
| 13 | 6,7,8-Trimethoxy-2H-1-benzopyran-2-one | C_12_H_12_O_5_ | 236.0685 | Flavonols | [6] |
| 14 | 4,4',6-Trihydroxyauran | C_16_H_14_O_5_ | 286.0841 | Flavonols | [6] |
| 15 | Isorhamnetin | C_16_H_12_O_7_ | 316.0583 | Flavonols | [7] |
| 16 | Hyperin | C_21_H_20_O_12_ | 464.0955 | Flavonols | [7] |
| 17 | Quercetin-3-O-D-galactoside-7-O-D-glucoside | C_27_H_30_O_17_ | 626.1483 | Flavonols | [8] |
| 18 | Quercetin-3-O-D-apiofuranosyl-D-galactoside | C_26_H_28_O_16_ | 596.1377 | Flavonols | [9] |
| 19 | 4’-Methoxy-quercetin | C_16_H_12_O_7_ | 316.0583 | Flavonols | [10] |
| 20 | Isoquercetin | C_15_H_10_O_27_ | 464.0955 | Flavonols | [11] |
| 21 | Myricetin-3-O-alpha-rhamnoside | C_21_H_20_O_12_ | 464.0955 | Flavonols | [3] |
| 22 | Azaleatin | C_16_H_12_O_7_ | 316.0583 | Flavonols | [6] |
| 23 | Melanettin | C_16_H_12_O_5_ | 284.0685 | Flavonols | [6] |
| 24 | Taxifolin | C_15_H_12_O_7_ | 304.0583 | Flavonols | [2] |
| 25 | 3,4',5,7-Tetrahydroxy flavanone | C_15_H_12_O_6_ | 288.0634 | Flavonols | [12] |
| 26 | Taxifolin-7-O-D-glucopyranoside | C_21_H_22_O_12_ | 466.1111 | Flavonols | [12] |
| 27 | 5-Hydroxy-7-methoxy-6-(2,3-epoxy-3-methylbutyl)-flavanone | C_22_H_20_O_6_ | 380.126 | Flavonols | [13] |
| 28 | Calycopteretin | C_15_H_10_O_5_ | 318.0376 | Flavonols | [14] |
| 29 | Apigenin | C_15_H_10_O_5_ | 270.0528 | Flavonols | [15] |
| 30 | Astragalin 6′′-O-gallate | C_28_H_24_O_15_ | 600.1115 | Flavonols | [15] |
| 31 | Kaempferol 3-O-arabinoside | C_20_H_18_O_10_ | 418.09 | Flavonols | [15] |
| 32 | Kaempferol-3-O-glucosyl-rhamnoside | C_27_H_30_O_15_ | 594.1585 | Flavonols | [15] |
| 33 | Kaempferol 3-O-caffeoyl-glucopyranoside | C_30_H_26_O_14_ | 610.1323 | Flavonols | [15] |
| 34 | Kaempferol 3-O-glucoside | C_30_H_26_O_13_ | 594.1585 | Flavonols | [15] |
| 35 | Kaempferol 3-O-galactoside | C_21_H_20_O_11_ | 448.1006 | Flavonols | [15] |
| 36 | Kaempferol 3,7-di-O-glucoside | C_27_H_30_O_16_ | 610.1534 | Flavonols | [15] |
| 37 | Kaempferol 7-O-rhamnoside | C_21_H_20_O_10_ | 432.1057 | Flavonols | [15] |
| 38 | Kaempferol 3-O-rhamnoside | C_21_H_20_O_10_ | 432.1057 | Flavonols | [15] |
| 39 | Kaempferol 3-O-rhamnoside 7-O-glucoside | C_27_H_30_O_15_ | 594.1585 | Flavonols | [15] |
| 40 | Quercetin-3-O-caffeoylgalactoside | C_30_H_26_O_15_ | 626.1272 | Flavonols | [15] |
| 41 | Quercetin-3-O-coumaroylgalactoside | C_30_H_26_O_14_ | 610.1323 | Flavonols | [15] |
| 42 | Quercetin 3-galactosyl-rhamnoside | C_27_H_30_O_16_ | 610.1534 | Flavonols | [15] |
| 43 | Quercetin3,7-di-O-rhamnoside | C_27_H_30_O_15_ | 594.1585 | Flavonols | [15] |
| 44 | Quercetin 3-O-galloyl-glucopyranoside | C_28_H_24_O_16_ | 616.1064 | Flavonols | [15] |
| 45 | Luteolin 7-O-glucoside | C_21_H_20_O_11_ | 448.1006 | Flavonols | [15] |
| 46 | Isorhamnetin 3-O-glucoside | C_22_H_22_O_12_ | 478.1111 | Flavonols | [15] |
| 47 | Isorhamnetin 7-O-glucoside | C_22_H_22_O_12_ | 478.1111 | Flavonols | [15] |
| 48 | Isorhamnetin 3-O-neohesperidoside | C_28_H_32_O_16_ | 624.169 | Flavonols | [15] |
| 49 | 4,4′,6-Trihydroxyaurone | C_15_H_10_O_5_ | 270.0528 | Flavonols | [15] |
| 50 | Apigenin-7-O-glucoside | C_21_H_20_O_10_ | 432.1057 | Flavonols | [16] |
| 51 | Quinic acid | C_7_H_12_O_6_ | 192.0634 | Phenolic acids | [17] |
| 52 | Chlorogenic acid | C_16_H_18_O_9_ | 354.0951 | Phenolic acids | [17] |
| 53 | 3,4,5-Tricaffeoylquinic acid | C_34_H_30_O_15_ | 678.1585 | Phenolic acids | [17] |
| 54 | 3,5-Dicaffeoylquinic acid/Isochlorogenic acid A | C_25_H_24_O_12_ | 516.1268 | Phenolic acids | [17] |
| 55 | 4,5-Dicaffeoylquinic acid/Isochlorogenic acid C | C_25_H_24_O_12_ | 516.1268 | Phenolic acids | [17] |
| 56 | 4-Caffeoyl-5-coumaroylquinic acid | C_25_H_24_O_11_ | 500.1319 | Phenolic acids | [17] |
| 57 | 4-Feruoyl-5-caffeoylquinic acid | C_26_H_26_O_12_ | 530.1424 | Phenolic acids | [17] |
| 58 | 1,3-Dicaffeoylquinic acid | C_25_H_24_O_12_ | 516.1268 | Phenolic acids | [18] |
| 59 | Neochlorogenic acid | C_17_H_18_O_9_ | 354.0951 | Phenolic acids | [19] |
| 60 | Cryptochlorogenic acid | C_17_H_18_O_9_ | 354.0951 | Phenolic acids | [19] |
| 61 | Methyl 4-hydroxy-3,5-dimethoxycinnamate | C_12_H_14_O_5_ | 238.0841 | Phenolic acids | [19] |
| 62 | 3,5-Dicaffeoyl-4-feruoylquinic acid | C_35_H_32_O_15_ | 692.1741 | Phenolic acids | [19] |
| 63 | Caffeic acid | C_9_H_8_O_4_ | 180.0423 | Phenolic acids | [19] |
| 64 | Ethyl caffeate | C_11_H_12_O_4_ | 208.0736 | Phenolic acids | [19] |
| 65 | p-Coumaric acid | C_9_H_8_O_3_ | 164.0473 | Phenolic acids | [19] |
| 66 | 3,4-Dicaffeoylquinic acid/Isochlorogenic acid B | C_25_H_20_O_12_ | 516.1268 | Phenolic acids | [15] |
| 67 | 5-O-Feruloylquinic acid | C_17_H_20_O_9_ | 368.1107 | Phenolic acids | [15] |
| 68 | 3-O-(4′-O-caffeoylglucosyl) Quinic acid | C_22_H_28_O_14_ | 516.1479 | Phenolic acids | [15] |
| 69 | 5-O-(3′-O-caffeoylglucosyl) Quinic acid | C_22_H_28_O_14_ | 516.1479 | Phenolic acids | [15] |
| 70 | Cinnamic acid | C_9_H_8_O_2_ | 148.0524 | Phenolic acids | [15] |
| 71 | Ferulic acid | C_10_H_10_O_4_ | 194.0579 | Phenolic acids | [15] |
| 72 | 6-O-cis-Caffeoyl-β-D-glucopyranose | C_15_H_18_O_9_ | 342.0951 | Phenolic acids | [15] |
| 73 | 6-O-trans-Caffeoyl-β-D-glucopyranose | C_15_H_18_O_9_ | 342.0951 | Phenolic acids | [15] |
| 74 | Gallic acid | C_7_H_6_O_5_ | 170.0215 | Phenolic acids | [15] |
| 75 | 4-O-β-D-Glucopyranosyl caffeic acid | C_15_H_16_O_10_ | 356.0744 | Phenolic acids | [15] |
| 76 | 4-p-Coumaroylquinic acid | C_17_H_22_O_7_ | 338.1365 | Phenolic acids | [15] |
| 77 | 3,4-Demethyleudesmin-4-O-glucoside | C_26_H_32_O_11_ | 520.1945 | Lignans | [17] |
| 78 | 5'-Hydroxyl-demethoxylpiperitol-4’-O-glccoside | C_25_H_28_O_11_ | 504.1632 | Lignans | [17] |
| 79 | 9α-Hhydroxy-d-sesamin-O-glcoside | C_26_H_28_O_12_ | 532.1581 | Lignans | [17] |
| 80 | 9α-Hydroxy-d-sesamin | C_20_H_18_O_7_ | 370.1053 | Lignans | [17] |
| 81 | d-Sesamin | C_20_H_18_O_6_ | 354.1103 | Lignans | [17] |
| 82 | 2'-Hydroxyasarinin-2'-O-rhamnosyl-glucoside | C_32_H_38_O_16_ | 694.2109 | Lignans | [17] |
| 83 | Cuscutoside A/B | C_31_H_37_O_17_ | 664.2003 | Lignans | [19] |
| 84 | Cuscutoside C | C_27_H_28_O_12_ | 532.1581 | Lignans | [19] |
| 85 | Cuscutoside D | C_37_H_47_O_21_ | 826.2532 | Lignans | [19] |
| 86 | Neocuscutoside A | C_37_H_47_O_21_ | 826.2532 | Lignans | [19] |
| 87 | Neocuscutoside B | C_35_H_40_O_21_ | 796.2426 | Lignans | [19] |
| 88 | Neocuscutoside C | C_33_H_40_O_17_ | 694.2109 | Lignans | [19] |
| 89 | Cuscutaresinol A | C_30_H_34_O_10_ | 554.2152 | Lignans | [19] |
| 90 | Cuscutaresinol B | C_30_H_34_O_10_ | 554.2152 | Lignans | [19] |
| 91 | Cuscutaresinol C | C_30_H_32_O_9_ | 536.2046 | Lignans | [19] |
| 92 | (+)-Pinoresinol | C_20_H_22_O_7_ | 358.1416 | Lignans | [19] |
| 93 | (-)-Pinoresinol | C_20_H_22_O_7_ | 358.1416 | Lignans | [19] |
| 94 | (+)-Epipinoresinol | C_20_H_22_O_7_ | 358.1416 | Lignans | [19] |
| 95 | Feruoylcaffeoyl-d-pinoresinol | C_39_H_37_O_12_ | 696.2207 | Lignans | [19] |
| 96 | Dicaffeoyl-d-pinoresinol | C_38_H_34_O_12_ | 682.205 | Lignans | [19] |
| 97 | Neosesamin | C_20_H_18_O_7_ | 370.1053 | Lignans | [19] |
| 98 | 5,5’-Dihydroxy-d-sesamin-5-O-glucoside | C_27_H_28_O_13_ | 548.153 | Lignans | [19] |
| 99 | 4-Methyl-3-methoxy-9α-hydroxyligballinol-O-glucoside | C_27_H_32_O_11_ | 504.1996 | Lignans | [19] |
| 100 | Piperitol-4'-O-glucoside | C_27_H_30_O_11_ | 518.1788 | Lignans | [19] |
| 101 | 9a-Hydroxy-d-sesamin-O-acetylglucoside | C_28_H_30_O_13_ | 574.1686 | Lignans | [15] |
| 102 | Sesaminol | C_20_H_18_O_7_ | 370.1052 | Lignans | [15] |
| 103 | (+)-Medioresinol | C_21_H_24_O_7_ | 356.126 | Lignans | [15] |
| 104 | (+)-Piperitol | C_20_H_20_O_6_ | 566.1999 | Lignans | [15] |
| 105 | 7-Methoxypinoresinol-4′-O-glucopyranoside | C_27_H_34_O_13_ | 518.1788 | Lignans | [15] |
| 106 | 5′-Methoxy-dimethyl piperonyl-4‘-O-glucoside | C_29_H_36_O_12_ | 504.1632 | Lignans | [15] |
| 107 | 3-Demethoxy-9α-hydroxypiperonol-O-diglucoside | C_25_H_28_O_11_ | 290.079 | Lignans | [15] |
| 108 | Epicatechol | C_15_H_14_O_6_ | 264.1838 | Lignans | [15] |
| 109 | Cuscutamine | C_15_H_14_N_2_O_3_ | 270.1004 | Alkaloids | [20] |
| 110 | Lupanine | C_15_H_24_N_2_O | 148.1889 | Alkaloids | [21] |
| 111 | Sparteine | C_15_H_26_N_2_ | 234.2096 | Alkaloids | [21] |
| 112 | Agroclavine | C_16_H_18_N_2_ | 238.147 | Alkaloids | [22] |
| 113 | Matrine | C_15_H_24_N_2_O | 248.1889 | Alkaloids | [22] |
| 114 | Cytisine | C_11_H_14_N_2_O | 190.1106 | Alkaloids | [21] |
| 115 | Methylcytisine | C_13_H_18_N_2_O | 218.1419 | Alkaloids | [6] |
| 116 | Sophoranol | C_15_H_24_N_2_O_2_ | 388.1522 | Alkaloids | [19] |
| 117 | Cuscutic acid A | C_38_H_78_O_21_ | 860.4253 | Resin glycosides | [19] |
| 118 | Cuscutic acid B | C_37_H_77_O_20_ | 830.4147 | Resin glycosides | [19] |
| 119 | Cuscutic acid C | C_38_H_78_O_20_ | 844.4304 | Resin glycosides | [19] |
| 120 | Cuscutic acid D | C_40_H_72_O_21_ | 888.4566 | Resin glycosides | [19] |
| 121 | Cuscutic resinoside A | C_31_H_54_O_12_ | 618.3615 | Resin glycosides | [19] |
| 122 | Cus-1 | C_39_H_78_O_19_ | 840.4355 | Resin glycosides | [19] |
| 123 | Cus-2 | C_41_H_72_O_19_ | 868.4668 | Resin glycosides | [19] |
| 124 | Cus-3 | C_37_H_77_O_18_ | 798.4249 | Resin glycosides | [19] |
| 125 | Cus-4 | C_37_H_72_O_18_ | 782.3936 | Resin glycosides | [19] |
| 126 | Cus-5 | C_43_H_73_O_17_N | 875.4879 | Resin glycosides | [19] |
| 127 | Cus-6 | C_47_H_79_O_18_N | 933.5297 | Resin glycosides | [19] |
| 128 | Cus-7 | C_45_H_79_O_17_N | 903.5192 | Resin glycosides | [19] |
| 129 | 11,14-Eicosadienoic acid methyl ester | C_21_H_38_O_2_ | 322.2872 | Fatty acids | [15] |
| 130 | Docosanoic acid methyl ester | C_23_H_46_O_2_ | 354.3498 | Fatty acids | [15] |
| 131 | Dotriacontanoic acid | C_32_H_64_O_2_ | 480.4906 | Fatty acids | [15] |
| 132 | Eicosanoic acid | C_20_H_40_O_2_ | 312.3028 | Fatty acids | [15] |
| 133 | Heptadecanoic acid | C_17_H_34_O_2_ | 270.2559 | Fatty acids | [15] |
| 134 | Methyl arachidate | C_21_H_42_O_2_ | 326.3185 | Fatty acids | [15] |
| 135 | Methyl cis-11-eicosenoate | C_21_H_40_O_2_ | 324.3028 | Fatty acids | [15] |
| 136 | Methyl heptadecanoate | C_18_H_36_O_2_ | 284.2715 | Fatty acids | [15] |
| 137 | Methyl hexadecanoate | C_17_H_34_O_2_ | 270.2559 | Fatty acids | [15] |
| 138 | Methyl linolelaidate | C_19_H_34_O_2_ | 294.2559 | Fatty acids | [15] |
| 139 | Methyl linolenate | C_19_H_32_O_2_ | 292.2402 | Fatty acids | [15] |
| 140 | Methyl myristate | C_15_H_30_O_2_ | 242.2246 | Fatty acids | [15] |
| 141 | Methyl oleate | C_19_H_36_O_2_ | 296.2715 | Fatty acids | [15] |
| 142 | Methyl palmitoleate | C_17_H_32_O_2_ | 268.2402 | Fatty acids | [15] |
| 143 | Methyl pentadecanoate | C_16_H_32_O_2_ | 256.2402 | Fatty acids | [15] |
| 144 | Methyl sinapate | C_12_H_14_O_5_ | 238.0841 | Fatty acids | [15] |
| 145 | Methyl stearate | C_19_H_38_O_2_ | 298.2872 | Fatty acids | [15] |
| 146 | Methyl tetracosanoate | C_25_H_50_O_2_ | 382.3811 | Fatty acids | [15] |
| 147 | Methyl tricosanoate | C_24_H_48_O_2_ | 368.3654 | Fatty acids | [15] |
| 148 | Palmitic acid | C_16_H_32_O_2_ | 256.2402 | Fatty acids | [15] |
| 149 | Stearic acid | C_18_H_36_O_2_ | 284.2715 | Fatty acids | [15] |
| 150 | LPC16-0 | C_24_H_50_NO_7_P | 495.3325 | Phospholipids | [15] |
| 151 | LPC18-1 | C_26_H_52_NO_7_P | 521.3481 | Phospholipids | [15] |
| 152 | LPC18-2 | C_26_H_50_NO_7_P | 519.3325 | Phospholipids | [15] |
| 153 | LPE18-0 | C_23_H_48_NO_7_P | 481.3168 | Phospholipids | [15] |
| 154 | 1-Palmitoyl-2-hydroxy-sn-glycero-3 | C_23_H_44_NO_7_P | 477.2861 | Phospholipids | [15] |

[1] Patel, S., Sharma, V., Chauhan, N. S., Dixit, V. K. (2012). An updated review on the parasitic herb of *Cuscuta reflexa* Roxb, *Journal of Chinese Integrative Medicine*, 10 (3), 249–258.

[2] Saini, P., Mithal, R., Menghani, E. (2015). A parasitic Medicinal plant *Cuscuta reflexa*: An Overview, *International Journal of Scientific & Engineering Research*, 6 (12), 951-959.

[3] Petra, H., Milan, Z., Tomas, B., Svajdlenka, E., Listy, C. (2009). Newly identified phenolic compounds in parasitic plants *Cuscuta europaea* and *Cuscuta campestris*, *Chemicke Listy*, 103 (3), 243–245.

[4] Dandapani, M., Franz, C., Nagarajan, S. (1989). Isorhamnetin 3-O-neohesperidoside from *Cuscuta reflexa*, *Indian Journal of Chemistry*, 28, 606–607.

[5] Chatterjee, D., Sahu, R. K. (2014). Chemical characterization of the flavonoid constituents of *Cuscuta reflexa*, UK, *Journal of Pharmaceutical and BioSciences*, 2 (3), 13–16.

[6] Vijikumar, S., Ramanathan, K., Devi, B.P. (2016). *Cuscuta reflexa* Roxb A wonderful miracle plant in ethnomedicine, *Indian Journal of Natural Sciences*, 1I (201), 0976–0997.

[7] Li, W. L., Zhang, Y., Bai, J., Xiang, Z., Ding, J. X., Ji, Y. B. (2013). Identification of chemical constituents in *Cuscuta chinensis* Using hplc-ESI/ Q-TOF MS/MS, Biotechnology: An Indian Journal, 8 (4), 563–567.

[8] Xiang, S. X., He, Z. S., Ye, Y. (2001). Furofuran lignans from *Cuscuta chinensis*, *Chinese Journal of Chemistry*, 19 (3), 282–285.

[9] Pan, S., Wang, X., Duan, W., Yu, Z., Zhang, L., Liu, W. (2013). Preparative isolation and purification of flavonoids from *Cuscuta chinensis* lam. by high-speed counter current chromatography, *Journal of Liquid Chromatography & Related Technologies*, 2162-2171.

[10] Ferraz, H., Silva, O., Magali G., Carvalho, R., Suffredini, I. B., Kato, E. T. M., Arakaki, F., Bacchi, E. M. (2011). Phytochemical study and evaluation of the antimicrobial activity and cytotoxicity of *Cuscuta racemosa*, ‌*[Revista Brasileira de Farmacognosia-Brazilian Journal of Pharmacognosy](https://www.baidu.com/s?rsv_dl=re_dqa_generate&sa=re_dqa_generate&wd=Revista%20Brasileira%20de%20Farmacognosia-Brazilian%20Journal%20of%20Pharmacognosy&rsv_pq=a69cfeae009cb4a6&oq=REVISTA%20BRASILEIRA%20DE%20FARMACOGNOSIA-BRAZILIAN%20JOURNAL%20OF%20PHARMACOGNOSY&rsv_t=5b14WMmZFlWwlAX8CBmhb9xXcQZKCMWkiodPugtc63XEj7tifeU4B/2KPQ8sK6eq1vQwQA&tn=40020637_1_dg&ie=utf-8)*, 21 (1), 41–46.

[11] Wang, Z., He, Z. S. (2017). Studies on the chemical constituents of the seed of chinese Dodder (*Cuscuta Chinensis*), *Chinese Traditional and Herbal Drugs*, 1998-2009.

[12] Mahmood, N., Piacente, S., Burke, A., Khan, A., Pizza, C. (1997). Constituents of *Cuscuta reflexa* are anti-HIV agent, *Antiviral Chemistry & Chemotherapy*, 8, 70–74.

[13] Tripathi, V. J., Yadav, S. B., Upadhyay, A. (2005). A new flavanone, reflexin, from *cuscuta reflexa* and its selective sensing of nitric oxide, *Applied Biochemistry and Biotechnology*, 127 (1), 63–67.

[14] Ahmad, A., Tandon, S., Xuan, T. D., Nooreen, Z. (2017). A Review on Phytoconstituents and Biological activities of *Cuscuta* species, *Biomedicine & Pharmacotherapy*, 92, 772-795.

[15] Chen, L. N., Hu, Y., Xin, S. G., Chen, Y. X., Song, Z., Song, H., Sun, X. M., Xu, B. L., Zhang, W. J., Wang, H., Yang, Z. H., Li, W. L. (2024), Research progress on chemical constituents, pharmacological effects of *Cuscutae Semen* and its quality marker prediction, *Chinese Traditional and Herbal Drugs*, 55(15), 5298-5314.

[16] Yong, S. K., Chang, B. S., Chang, M. K. (2000). Antioxidative Constituents from the Seeds of *Cuscuta chinensis*, *Natural Product Sciences*, 6(3), 135–138.

[17] Ye, M., Yan, Y. N., Guo, D. A. (2005). Characterization of phenolic compounds in the Chinese herbal drug Tu-Si-Zi by liquid chromatography coupled to electrospray ionization mass spectrometry, *Rapid Communications in Mass Spectrometry*, 19(11), 1469-1484.

[18] Li, W. D., Ding, J. X., Zang, B. S., Gao, S., Yang, B., Ji, Y. B. (2014). Analysis of Purified Active Ingredients of Estrogen Mimic Effect in *Cuscuta chinensis* by HPLC-QTOF MS/MS, *Chinese Pharmaceutical Journal*, 49(20), 1791-1795.

[19] Wang, M., Xu, X. Y., Wang, H. D., Wang, H. M., Liu, M. Y., Hu, W. D., Chen, B. X., Jiang, M. T., Qi, J., Li, X. H., Yang, W. Z., Gao, X. M. (2022). A multi-dimensional liquid chromatography/high-resolution mass spectrometry approach combined with computational data processing for the comprehensive characterization of the multicomponents from *Cuscuta chinensis*, *Journal of Chromatography A*, 1675, 463162.

[20] Thomas, S., Velmurugan, C., Kumar, B.S.A. (2015). Evaluation of anxioltic effect of whole plant of *Cuscuta reflexa*, *World Journal of Pharmacy and Pharmaceutical Sciences*, 4(8), 1245-1253.

[21] Bäumel, P., Witte, L., Czygan, F. C, Proksch, P. (1994). Transfer of qinolizidine alkaloids from various host plants of the Fabaceae to parasitizing *Cuscuta* species, *Biochemical Systematics and Ecology*, 22(7), 647-656.

[22] Garcia, M. R., Erazo, G., Peña, R. (1995). Flavonoids and alkaloids from *Cuscut*a (Cuscutaceae), *Biochemical Systematics and Ecology*, 23(5), 571-572.

**Table S3.** Characterization of chemical constituents of *Cuscuta* seeds

| NO. | tR (min) | Formula | Selected ion | Measured mass | Calculated mass | Mass error /mDa | MS fragmentation | Identification | Structure |
| --- | --- | --- | --- | --- | --- | --- | --- | --- | --- |
| 1 | 0.64 | C_18_H_32_O_16_ | [M+Na]+ | 527.1578 | 527.1588 | -1 | 365.1057, 347.0950, 203.0530, 185.0423 | Raffinose* | Oligosaccharides |
| 2 | 0.7 | C_12_H_22_O_11_ | [M+Na]+ | 365.1063 | 365.106 | 0.3 | 203.0533, 185.0425 | Sucrose* | Oligosaccharides |
| 3 | 0.74 | C_30_H_46_O_22_ | [M+NH_4_]+ | 776.2808 | 776.2824 | -1.6 | 399.1055, 325.1134, 163.0602, 145.0503, 127.0398, 109.0303, 97.0297, 85.0286 | Hydroxyphenyl+4Hex# | Oligosaccharides |
|  |  |  | [M+HCOO]- | 803.2463 | 803.2457 | 0.6 | 757.2401, 647.2045, 569.1757, 545.1759, 383.1196, 341.1135, 323.0932, 221.0665, 179.0563, 161.0463, 125.0247, 101.0242 |  |  |
| 4 | 1 | C_6_H_8_O_7_ | [M-H]- | 191.0206 | 191.0192 | 1.4 | 111.0088 | Citric acid | Others |
| 5 | 1.44 | C_10_H_13_N_5_O_5_ | [M+H]+ | 284.0991 | 284.0995 | -0.4 | 152.0569 | Guanosin | Others |
|  |  |  | [M-H]- | 282.0842 | 282.0838 | 0.4 | 150.0424 |  |  |
| 6 | 1.64 | C_30_H_46_O_22_ | [M+NH_4_]+ | 776.2815 | 776.2824 | -0.9 | 399.1047, 325.1130, 163.0605, 145.0501, 127.0396, 109.0291, 97.0290, 85.0290 | Hydroxyphenyl+4Hex# | Oligosaccharides |
|  |  |  | [M+HCOO]- | 803.2471 | 803.2457 | 1.4 | 757.2407, 647.2029, 569.1721, 545.1722, 485.1512, 383.1196, 341.1090, 323.0984, 263.0778, 221.0670, 179.0560, 161.0455, 125.0245, 101.0243 |  |  |
| 7 | 1.82 | C_20_H_34_O_15_ | [M+HCOO]- | 559.1876 | 559.1874 | 0.2 | 393.1406, 351.1282, 223.0820, 205.0686, 163.0628 | 2MePen+AcetylHex# | Oligosaccharides |
| 8 | 2.1 | C_24_H_42_O_18_ | [M-H]- | 617.2296 | 617.2293 | 0.3 | 451.1853, 433.1718, 407.1560, 365.1454, 351.1303, 333.1208, 315.1099, 281.1248, 263.1146, 237.0983, 205.0722, 187.0620, 163.0611, 145.0518, 127.0405, 117.0558, 99.0454 | 3MePenHex# | Oligosaccharides |
| 9 | 2.2 | C_22_H_28_O_14_ | [M-H]- | 515.1404 | 515.1401 | 0.3 | 353.0883, 341.0880, 191.0562, 179.0354, 173.0462, 135.0453 | β-D-glucopyranosyl-caffeoyl quinic acid | Phenolic acids |
| 10 | 2.31 | C_24_H_42_O_18_ | [M-H]- | 617.2287 | 617.2293 | -0.6 | 451.1740, 433.1718, 407.1564, 365.1449, 351.1305, 333.1196, 315.1103, 281.1253, 263.1148, 237.0988, 205.0724, 187.0619, 163.0613, 143.0358, 127.0406, 117.0557, 99.0453 | 3MePenHex# | Oligosaccharides |
| 11 | 2.34 | C_28_H_38_O_19_ | [M-H]- | 677.1932 | 677.1929 | 0.3 | 485.1229, 353.0884, 191.0563, 179.0355, 173.0452, 161.0281, 135.0461 | di-β-D-glucopyranosyl-caffeoyl quinic acid | Phenolic acids |
| 12 | 2.46 | C_22_H_28_O_14_ | [M-H]- | 515.1439 | 515.1401 | 3.8 | 353.0879, 191.0569, 179.0352, 161.0247, 135.0452 | β-D-glucopyranosyl-caffeoyl quinic acid | Phenolic acids |
| 13 | 2.6 | C_6_H_6_O_3_ | [M+H]+ | 127.0399 | 127.0395 | 0.4 | 109.0286 | 5-HMF | Others |
| 14 | 2.66 | C_27_H_38_O_19_ | [M+H]+ | 665.1924 | 665.1929 | -0.5 | 503.1420, 459.1485, 341.0900, 221.0473, 179.0353, 161.0240, 145.0294, 135.0461 | O-caffeoyl-raffinose | Phenolic acids |
| 15 | 2.69 | C_27_H_38_O_18_ | [M+HCOO]- | 695.204 | 695.2035 | 0.6 | 487.1452, 325.0936, 307.0831, 265.0718, 235.0616, 205.0509, 163.0401, 145.0295, 119.0500, 117.0343 | O-p-coumaroyl-di-β-D-glucopyranosyl-β-D-glucopyranoside | Phenolic acids |
| 16 | 2.72 | C_15_H_18_O_9_ | [M-H]- | 341.088 | 341.0873 | 0.7 | 179.0359, 135.0453 | O-caffeoyl-β-D-glucopyranoside | Phenolic acids |
| 17 | 2.73 | C_16_H_18_O_9_ | [M-H]- | 353.0876 | 353.0873 | 0.3 | 191.0560, 179.0351, 135.0451 | Neochlorogenic acid* | Phenolic acids |
| 18 | 2.78 | C_15_H_18_O_8_ | [M+HCOO]- | 371.098 | 371.0978 | 0.2 | 163.0404, 119.0503 | O-p-coumaroyl-β-D-glucopyranosyl | Phenolic acids |
| 19 | 2.83 | C_22_H_28_O_14_ | [M-H]- | 515.14 | 515.1401 | -0.1 | 353.0882, 341.0883, 191.0562, 179.0354, 173.0458, 135.0454 | β-D-glucopyranosyl-caffeoyl quinic acid | Phenolic acids |
| 20 | 2.92 | C_30_H_34_O_10_ | [M+HCOO]- | 599.2172 | 599.2129 | 4.3 | 337.2070, 281.1225, 263.1201, 163.0622, 127.0404, 117.0561 | Cuscutaresinol A/Cuscutaresinol B | Lignans |
| 21 | 2.97 | C_28_H_40_O_19_ | [M+HCOO]- | 725.2136 | 725.214 | -0.4 | 517.1563, 355.1058, 337.0999, 295.0819, 265.0734, 235.0625, 193.0517, 175.0408, 134.0376 | O-feruloyl-raffinose | Phenolic acids |
| 22 | 3.09 | C_22_H_28_O_14_ | [M-H]- | 515.1393 | 515.1401 | -0.8 | 353.0883, 341.0900, 191.0563, 179.0360, 173.0462, 161.0247, 135.0454 | β-D-glucopyranosyl-caffeoyl quinic acid | Phenolic acids |
| 23 | 3.32 | C_16_H_18_O_8_ | [M-H]- | 337.0931 | 337.0923 | 0.8 | 163.0419, 119.0508 | O-p-coumaroyl quinic acid | Phenolic acids |
| 24 | 3.38 | C_27_H_30_O_17_ | [M-H]- | 625.1409 | 625.1405 | 0.4 | 463.0869, 301.0354, 299.0201, 151.0019 | Quercetin-3-O-galactoside-7-O-glucoside | Flavonols |
| 25 | 3.46 | C_16_H_18_O_9_ | [M+H]^+^ | 355.1031 | 355.1029 | 0.2 | 163.0400, 145.0294, 135.0449, 89.0365 | Chlorogenic acid* | Phenolic acids |
|  |  |  | [M-H]- | 353.0883 | 353.0873 | 1 | 191.0563 |  |  |
| 26 | 3.55 | C_27_H_30_O_17_ | [M-H]- | 625.1403 | 625.1405 | -0.2 | 463.0862, 301.0365, 299.0197, 243.0381, 191.0553 | Quercetin-3-O-glucoside-7-O-glucoside | Flavonols |
| 27 | 3.59 | C_21_H_28_O_13_ | [M-H]- | 487.1445 | 487.1452 | -0.7 | 307.0758, 265.0729, 235.0629, 205.0496, 163.0410, 145.0297, 119.0512, 117.0344 | O-p-coumaroyl-β-D-glucopyranosyl-β-D-glucopyranoside | Phenolic acids |
| 28 | 3.64 | C_26_H_44_O_19_ | [M-H]- | 659.2403 | 659.2399 | 0.4 | 433.1696, 407.1564, 351.1294, 333.1185, 281.1225, 263.1123, 205.0720, 187.0622, 163.0616, 143.0350, 117.0557, 99.0244 | AcetylMePen2MePenHex# | Oligosaccharides |
| 29 | 3.65 | C_16_H_18_O_9_ | [M-H]- | 353.0878 | 353.0873 | 0.5 | 191.0517, 173.0512, 149.0617, 134.0382 | Cryptochlorogenic acid* | Phenolic acids |
| 30 | 3.69 | C_17_H_20_O_9_ | [M-H]- | 367.1034 | 367.1029 | 0.5 | 193.0512, 191.0562, 173.0459, 134.0375, 93.0350 | Feruloylquinic acid | Phenolic acids |
| 31 | 3.75 | C_27_H_32_O_15_ | [M-H]- | 595.1667 | 595.1663 | 0.4 | 433.1154, 323.0807, 179.0339, 161.0257, 133.0273 | C_21_H_20_O_10_+glucoside# | Phenolic acids |
| 32 | 3.8 | C_15_H_18_O_8_ | [M-H]- | 325.0928 | 325.0923 | 0.5 | 163.0387, 119.0496 | O-p-coumaroyl-β-D-glucopyranoside | Phenolic acids |
| 33 | 3.86 | C_9_H_8_O_4_ | [M-H]- | 179.035 | 179.0344 | 0.6 | 135.0451, 107.0507 | Caffeic acid* | Phenolic acids |
| 34 | 3.95 | C_21_H_28_O_13_ | [M-H]- | 487.1445 | 487.1452 | -0.7 | 341.1157, 307.0758, 265.0729, 235.0629, 205.0496, 163.0410, 145.0297, 119.0512, 117.0344 | O-p-coumaroyl-β-D-glucopyranosyl-β-D-glucopyranoside | Phenolic acids |
| 35 | 3.98 | C_27_H_30_O_16_ | [M-H]- | 609.1456 | 609.1456 | 0 | 447.0948, 283.0251, 255.0303, 227.0331, 161.0255 | Luteolin 3',7-di-O-β-D-glucoside | Flavonols |
| 36 | 4.16 | C_15_H_12_N_2_O_4_ | [M+H]+ | 285.0878 | 285.0875 | 0.3 | 267.0771,249.0668, 221.0718, 193.0768, 166.0659 | hydroxyl-cuscutamine-4H# | Alkaloids |
|  |  |  | [M-H]- | 283.0728 | 283.0719 | 0.9 | 221.0728, 195.0934, 193.0779, 180.0697 |  |  |
| 37 | 4.19 | C_43_H_56_O_26_ | [M+HCOO]- | 1033.3044 | 1033.3036 | 0.8 | 825.2454, 663.1964, 603.1747, 517.1547, 501.1420, 355.1110, 337.0938, 307.0807, 295.0836, 235.0607, 193.0508, 175.0406, 163.0398, 145.0299 | O-feruloyl-O-p-coumaroyl-stachyose | Phenolic acids |
| 38 | 4.24 | C_42_H_54_O_26_ | [M-H]- | 973.2827 | 973.2825 | 0.2 | 811.2306, 649.1775, 487.1348, 323.0780, 265.0731, 235.0621, 205.0514, 179.0356, 163.0406, 145.0297, 119.0505 | O-p-coumaroyl-O-caffeoyl-stachyose | Phenolic acids |
| 39 | 4.36 | C_42_H_54_O_25_ | [M+HCOO]- | 1003.2946 | 1003.2931 | 1.5 | 795.2348, 633.1818, 573.1606, 513.1389, 487.1442, 471.1288, 307.0817, 265.0724, 235.0609, 205.0508, 163.0400, 145.0292, 119.0506 | di-O-p-coumaroyl-stachyose | Phenolic acids |
| 40 | 4.45 | C_42_H_54_O_26_ | [M-H]- | 973.2834 | 973.2825 | 0.9 | 811.2294, 649.1882, 487.1370, 323.0769, 265.0749, 179.0328, 163.0396, 145.0304 | O-p-coumaroyl-O-caffeoyl-stachyose | Phenolic acids |
| 41 | 4.45 | C_43_H_56_O_26_ | [M+HCOO]- | 1033.3047 | 1033.3036 | 1.1 | 825.2456, 663.1964, 603.1731, 543.1512, 517.1589, 501.1410, 487.1461, 337.0938, 307.0840, 265.0706, 235.0619, 205.0533, 193.0512, 175.0406, 163.0407, 145.0305 | O-feruloyl-O-p-coumaroyl-stachyose | Phenolic acids |
| 42 | 4.45 | C_32_H_38_O_21_ | [M-H]- | 757.1829 | 757.1827 | 0.2 | 625.1438, 595.1300, 301.0335, 300.0280, 271.0241, 255.0293, 151.0037 | Quercetin-O-apiofuranosyl-O-diglucoside | Flavonols |
| 43 | 4.53 | C_15_H_16_N_2_O_4_ | [M+H]+ | 289.1186 | 289.1188 | -0.2 | 272.0920, 226.0870, 216.1025, 180.0815, 170.0972, 168.0811, 155.0731, 130.0661 | hydroxyl-cuscutamine# | Alkaloids |
|  |  |  | [M-H]- | 287.1037 | 287.1032 | 0.5 | 269.0934, 243.1140, 225.1039, 199.1239, 156.0812, 116.0513, 114.0558, |  |  |
| 44 | 4.56 | C_42_H_54_O_25_ | [M+HCOO]- | 1003.2943 | 1003.2931 | 1.2 | 795.2375, 633.1890, 513.1389, 487.1461, 471.1300, 307.0832, 265.0733, 235.0613, 205.0515, 163.0404, 145.0295 | di-O-p-coumaroyl-stachyose | Phenolic acids |
| 45 | 4.6 | C_37_H_44_O_21_ | [M-H]- | 823.2308 | 823.2297 | 1.1 | 661.1782, 649.1840, 487.1264, 395.0982, 323.0786, 191.0563, 179.0360, 173.0457, 163.0409, 135.0449 | di-β-D-glucopyranosyl-O-p-coumaroyl-caffeoyl quinic acid | Phenolic acids |
| 46 | 4.62 | C_17_H_20_O_9_ | [M-H]- | 367.1039 | 367.1029 | 1 | 193.0512, 191.0562, 173.0459, 134.0375, 93.0350 | Feruloylquinic acid | Phenolic acids |
| 47 | 4.65 | C_37_H_44_O_20_ | [M+HCOO]- | 853.2413 | 853.2402 | 1.1 | 807.2432, 691.1915, 645.1840, 499.1504, 483.1315, 337.0951, 319.0831, 191.0574, 173.0464, 163.0412, 137.0245, 119.0495 | di-β-D-glucopyranosyl-di-O-p-coumaroyl-quinic acid | Phenolic acids |
| 48 | 4.67 | C_43_H_56_O_26_ | [M+HCOO]- | 1033.3051 | 1033.3036 | 1.5 | 825.2478, 663.1864, 543.1574, 517.1548, 501.1407, 487.1474, 337.0938, 307.0830, 265.0684, 235.0622, 205.0484, 193.0504, 175.0400, 163.0404, 145.0291 | O-feruloyl-O-p-coumaroyl-stachyose | Phenolic acids |
| 49 | 4.7 | C_28_H_32_O_15_ | [M-H]- | 607.1663 | 607.1663 | 0 | 445.1332, 281.0714, 163.0401, 137.0266, 119.0505 | C_22_H_22_O_10_+glucoside# | Phenolic acids |
| 50 | 4.74 | C_15_H_16_N_2_O_4_ | [M+H]+ | 289.1186 | 289.1188 | 0.2 | 272.0915, 226.0875, 216.1024, 180.0808, 170.0967, 168.0818, 155.0734, 130.0653 | hydroxyl-cuscutamine# | Alkaloids |
|  |  |  | [M-H]- | 287.1041 | 287.1032 | 0.9 | 269.0941, 243.1150, 225.1036, 199.1251, 156.0815, 116.0510, 114.0554, 92.0508 |  |  |
| 51 | 4.85 | C_31_H_36_O_16_ | [M-H]- | 663.1934 | 663.1925 | 0.9 | 501.1412, 383.1089, 341.0881, 323.0783, 307.0818, 221.0457, 193.0511, 179.0349, 161.0254, 145.0302, 135.0450 | O-feruloyl-O-p-coumaroyl-sucrose | Phenolic acids |
| 52 | 4.87 | C_9_H_8_O_3_ | [M-H]- | 163.0407 | 163.0395 | 1.2 | 119.0503 | p-Coumaric acid* | Phenolic acids |
| 53 | 4.89 | C_30_H_34_O_17_ | [M-H]- | 665.1721 | 665.1718 | 0.3 | 503.1196, 341.0886, 323.0778, 221.0469, 179.0355, 161.0247, 135.0455 | di-O-caffeoyl-sucrose | Phenolic acids |
| 54 | 4.91 | C_15_H_12_N_2_O_4_ | [M+H]+ | 285.0868 | 285.0875 | -0.7 | 267.0765, 239.0818, 221.0713, 193.0763 | hydroxyl-cuscutamine-4H# | Alkaloids |
|  |  |  | [M-H]- | 283.0727 | 283.0719 | 0.8 | 239.0825, 221.0729, 195.0930, 193.0775, 180.0695 |  |  |
| 55 | 4.91 | C_30_H_34_O_16_ | [M-H]- | 649.1769 | 649.1769 | 0 | 487.1255, 323.0782, 179.0357, 163.0406, 161.0252, 145.0303, 119.0506 | O-caffeoyl-O-p-coumaroyl-sucrose | Phenolic acids |
| 56 | 5.01 | C_16_H_16_N_2_O_6_ | [M+H]+ | 333.1094 | 333.1087 | 0.7 | 316.0813, 289.1182, 260.0921, 242.0816, 217.0977, 196.0762, 186.0919, 168.0811, 154.0655 | hydroxyl-carboxyl-cuscutamine# | Alkaloids |
|  |  |  | [M-H]- | 331.0942 | 331.093 | 1.2 | 313.0836, 287.1042, 269.0938, 243.1146, 225.1039, 199.1248, 156.0813, 116.0509, 114.0559, 92.0505 |  |  |
| 57 | 5.11 | C_30_H_34_O_16_ | [M-H]- | 649.1769 | 649.1769 | 0 | 487.1251, 323.0780, 221.0462, 179.0354, 163.0405, 161.0247, 135.0454, 119.0502 | O-caffeoyl-O-p-coumaroyl-sucrose | Phenolic acids |
| 58 | 5.11 | C_30_H_34_O_15_ | [M-H]- | 633.1821 | 633.1819 | 0.2 | 471.1306, 307.0830, 205.0510, 163.0405, 145.0297, 119.0503 | di-O-p-coumaroyl-sucrose | Phenolic acids |
| 59 | 5.21 | C_26_H_28_O_16_ | [M-H]- | 595.1294 | 595.1305 | -1.1 | 301.0335, 300.0282, 271.0254, 255.0305, 151.0040 | Quercetin-3-O-β-D-apiofuranosyl-(1→2)-β-D-glucoside | Flavonols |
| 60 | 5.23 | C_31_H_36_O_16_ | [M-H]- | 663.1909 | 663.1925 | -1.6 | 501.1412, 337.0948, 193.0507, 175.0411, 163.0406, 145.0302, 119.0506 | O-feruloyl-O-p-coumaroyl-sucrose | Phenolic acids |
| 61 | 5.3 | C_31_H_34_O_17_ | [M-H]- | 677.1722 | 677.1718 | 0.4 | 515.1226, 353.0881, 323.0782, 191.0565, 179.0356, 173.0456, 161.0254, 135.0454 | β-D-glucopyranosyl-di-caffeoyl quinic acid | Phenolic acids |
| 62 | 5.35 | C_30_H_34_O_15_ | [M-H]- | 633.1821 | 633.1819 | 0.2 | 471.1301, 307.0831, 205.0514, 163.0403, 145.0298, 119.0503 | di-O-p-coumaroyl-sucrose | Phenolic acids |
| 63 | 5.5 | C_31_H_36_O_16_ | [M-H]- | 663.1934 | 663.1925 | 0.9 | 501.1411, 337.0925, 323.0783, 307.0837, 235.0616, 205.0529, 193.0513, 179.0349, 178.0272, 175.0400, 163.0401, 149.0613, 145.0302, 134.0380, 119.0508 | O-feruloyl-O-p-coumaroyl-sucrose | Phenolic acids |
| 64 | 5.55 | C_31_H_34_O_17_ | [M-H]- | 677.1722 | 677.1718 | 0.4 | 515.1312, 487.1460, 353.0898, 341.0893, 307.0834, 191.0595, 179.0360, 173.0460, 163.0407, 145.0298, 135.0467 | β-D-glucopyranosyl-di-caffeoyl quinic acid | Phenolic acids |
| 65 | 5.6 | C_21_H_20_O_12_ | [M+H]+ | 465.1024 | 465.1033 | -0.9 | 303.0505, 257.0448, 229.0503, 165.0188, 153.0193 | Hyperoside* | Flavonols |
|  |  | C_21_H_20_O_12_ | [M-H]- | 463.0888 | 463.0877 | 1.1 | 301.0345, 300.0282, 271.0250, 255.0301, 243.0302, 227.0350, 178.9987, 151.0038 |  |  |
| 66 | 5.75 | C_21_H_20_O_12_ | [M+H]+ | 465.1021 | 465.1033 | -1.2 | 303.0499, 257.0474, 229.0503, 165.0195, 153.0186 | Isoquercitrin* | Flavonols |
|  |  | C_21_H_20_O_12_ | [M-H]- | 463.0885 | 463.0877 | 0.8 | 301.0349, 300.0283, 271.0253, 255.0306, 243.0299, 178.9992, 151.0039 |  |  |
| 67 | 5.83 | C_36_H_36_O_19_ | [M-H]- | 771.1773 | 771.1773 | 0 | 609.1232, 463.0872, 301.0340, 300.0275, 271.0254, 255.0291, 243.0314, 178.9992, 151.0034 | Quercetin-O-galactopyranoside-O-coumaroyl-O-galactopyranoside | Flavonols |
| 68 | 5.83 | C_26_H_28_O_15_ | [M-H]- | 579.1336 | 579.135 | -1.4 | 285.0406, 284.0336, 255.0295, 227.0339 | kaemferol-O-galactopyranoside-O-apiofuranoside | Flavonols |
| 69 | 5.95 | C_26_H_28_O_15_ | [M-H]- | 579.1353 | 579.135 | 0.3 | 285.0349, 284.0326, 255.0301, 227.0371 | kaemferol-O-galactopyranoside-O-apiofuranoside | Flavonols |
| 70 | 5.97 | C_36_H_44_O_20_ | [M-H]- | 795.2347 | 795.2348 | -0.1 | 633.1841, 573.1606, 513.1494, 487.1477, 471.1296, 307.0832, 265.0726, 235.0604, 205.0519, 163.0404, 145.0295, 119.0507 | di-O-p-coumaroyl-raffinose | Phenolic acids |
| 71 | 6.07 | C_27_H_30_O_16_ | [M-H]- | 609.1456 | 609.1456 | 0 | 315.0508, 314.0435, 299.0208, 285.0475, 271.0250, 243.0314, 145.0293 | Nelumboroside A | Flavonols |
| 72 | 6.19 | C_36_H_44_O_20_ | [M-H]- | 795.2357 | 795.2348 | 0.9 | 633.1853, 573.1581, 513.1418, 487.1458, 471.1306, 307.0844, 265.0716, 235.0616, 205.0513, 163.0408, 145.0303, 119.0508 | di-O-p-coumaroyl-raffinose | Phenolic acids |
| 73 | 6.19 | C_37_H_46_O_21_ | [M-H]- | 825.2462 | 825.2453 | 0.9 | 663.1941, 603.1779, 543.1476, 501.1404, 487.1463, 337.0941, 307.0824, 265.0730, 235.0616, 205.0513, 193.0500, 175.0427, 163.0409, 145.0301, 119.0506 | O-feruloyl-O-p-coumaroyl-raffinose | Phenolic acids |
| 74 | 6.22 | C_23_H_22_O_13_ | [M-H]- | 505.0979 | 505.0982 | -0.3 | 463.0923, 301.0370, 300.0284, 271.0255, 255.0298 | Quercetin-O-acetyl-β-D-galactopyranoside | Flavonols |
| 75 | 6.24 | C_21_H_20_O_11_ | [M-H]- | 447.0928 | 447.0927 | 0.1 | 285.0395, 284.0333, 255.0306, 227.0351, 151.0040 | Astragalin isomer | Flavonols |
| 76 | 6.48 | C_45_H_50_O_23_ | [M-H]- | 957.2664 | 957.2665 | -0.1 | 795.2155, 633.1819, 471.1297, 307.0833, 163.0403, 145.0302, 119.0510 | β-D-glucopyranosyl-di-O-p-coumaroyl-raffinose | Phenolic acids |
| 77 | 6.54 | C_24_H_24_O_11_ | [M-H]- | 487.1244 | 487.124 | 0.4 | 323.0777, 221.0474, 179.0353, 163.0403, 161.0248, 135.0462, 133.0291, 119.0504 | O-caffeoyl-O-p-coumaroyl-β-D-glucopyranoside | Phenolic acids |
| 78 | 6.55 | C_21_H_20_O_11_ | [M-H]- | 447.0925 | 447.0927 | -0.2 | 285.0395, 284.0331, 255.0301, 227.0351, 151.0041 | Astragalin* | Flavonols |
| 79 | 6.61 | C_22_H_22_O_12_ | [M+H]+ | 479.1186 | 479.119 | -0.4 | 317.0662, 302.0419, 153.0181 | Isorhamnetin-3-O-Glucoside* | Flavonols |
|  |  | C_22_H_22_O_12_ | [M-H]- | 477.1029 | 477.1033 | -0.4 | 315.0483, 314.0432, 299.0199, 285.0407, 271.0250, 257.0460, 243.0300, 227.0348, 215.0360, 151.0042 |  |  |
| 80 | 6.76 | C_22_H_22_O_12_ | [M+H]+ | 479.1183 | 479.119 | -0.7 | 317.0661, 302.0434, 285.0404, 153.0194 | Isorhamnetin-7-O-Glucoside* | Flavonols |
|  |  | C_22_H_22_O_12_ | [M-H]- | 477.1034 | 477.1033 | 0.1 | 315.0482, 314.0432, 299.0199, 285.0406, 271.0248, 257.0456, 243.0299, 227.0352, 215.0347, 151.0039 |  |  |
| 81 | 6.81 | C_21_H_20_O_11_ | [M-H]- | 447.093 | 447.0927 | 0.3 | 285.0404, 257.0469, 255.0305, 227.0354, 151.0025 | Kaempferol-O-galactoside | Flavonols |
| 82 | 6.84 | C_23_H_22_O_13_ | [M-H]- | 505.0988 | 505.0982 | 0.6 | 463.0877, 301.0276, 300.0276, 271.0253, 255.0299, 243.0308, 178.9999, 151.0038 | Quercetin-O-acetyl-β-D-galactopyranoside | Flavonols |
| 83 | 6.9 | C_32_H_36_O_16_ | [M-H]- | 675.1918 | 675.1925 | -0.7 | 513.1394, 499.1455, 337.0932, 193.0498, 173.0462, 163.0412, 119.0495 | β-D-glucopyranosyl-O-feruoyl-O-p-coumaroyl quinic acid | Phenolic acids |
| 84 | 7 | C_9_H_16_O_4_ | [M-H]- | 187.0976 | 187.097 | 0.6 | 125.0971 | Azelaic acid* | Organic acids |
| 85 | 7.02 | C_41_H_48_O_19_ | [M-H]- | 843.2716 | 843.2712 | 0.4 | 681.2247, 323.0802, 161.0249 | β-D-glucopyranosyl+C20H22O6+β-D-glucopyranosyl+caffeic acid# | Phenolic acids |
| 86 | 7.1 | C_30_H_32_O_14_ | [M+HCOO]- | 661.1757 | 661.1769 | -1.2 | 615.1726, 555.1483, 513.1443, 471.1300, 307.0839, 265.0779, 205.0515, 163.0410, 161.0611, 145.0296, 119.0501 | O-acetyl-p-coumaroyl-di-acetyl-dihydrocaffeoyl-β-D-glucopyranoside | Phenolic acids |
| 87 | 7.16 | C_25_H_24_O_12_ | [M-H]- | 515.1185 | 515.119 | -0.5 | 353.0876, 191.0558, 173.0453, 135.0447 | Isochlorogenic acid C* | Phenolic acids |
| 88 | 7.2 | C_23_H_22_O_13_ | [M-H]- | 505.0986 | 505.0982 | 0.4 | 463.0857, 300.0278, 271.0261, 255.0291, 243.0292, 173.0459, 151.0025 | Quercetin-O-acetyl-β-D-galactopyranoside | Flavonols |
| 89 | 7.28 | C_10_H_10_O_4_ | [M-H]- | 193.0509 | 193.0501 | 0.8 | 161.0249, 134.0368, 133.0299 | Ferulic acid* | Phenolic acids |
| 90 | 7.35 | C_24_H_24_O_10_ | [M-H]- | 487.1245 | 487.124 | 0.5 | 307.0833, 179.0354, 145.0304, 135.0454 | O-caffeoyl-O-p-coumaroyl-β-D-glucopyranoside | Phenolic acids |
| 91 | 7.37 | C_41_H_48_O_18_ | [M-H]- | 827.2768 | 827.2762 | 0.6 | 665.2178, 307.0785, 179.0341, 163.0375, 145.0314 | C_35_H_38_O_13_+glucopyranoside# | Phenolic acids |
| 92 | 7.37 | C_28_H_24_O_14_ | [M-H]- | 583.1089 | 583.1088 | 0.1 | 463.0899, 301.0338, 300.0280, 271.0256, 255.0334, 243.0397, 178.9975, 151.0043 | p-Hydroxybenzoyl-quercetin-3-O-galactoside | Flavonols |
| 93 | 7.43 | C_30_H_26_O_15_ | [M-H]- | 625.1198 | 625.1193 | 0.5 | 463.0891, 301.0354, 300.0281, 271.0259, 255.0231, 178.9992, 151.0031 | Quercetin 3-O-(6’’-caffeoyl)-β-D-glucopyranoside | Flavonols |
| 94 | 7.43 | C_41_H_48_O_19_ | [M-H]- | 843.2722 | 843.2712 | 1 | 681.2228, 323.0800, 179.0359, 161.0249 | β-D-glucopyranosyl+C20H22O6+β-D-glucopyranosyl+caffeic acid# | Phenolic acids |
| 95 | 7.64 | C_24_H_24_O_10_ | [M-H]- | 471.1295 | 471.1291 | 0.4 | 307.0832, 163.0403, 145.0299, 119.0504 | di-O-p-coumaroyl-β-D-glucopyranoside | Phenolic acids |
| 96 | 7.67 | C_41_H_48_O_18_ | [M-H]- | 827.2769 | 827.2762 | 0.7 | 665.2316, 307.0848, 193.0511, 163.0399, 145.0288 | C_35_H_38_O_13_+glucopyranoside# | Phenolic acids |
| 97 | 7.76 | C_31_H_36_O_15_ | [M+HCOO]- | 693.2027 | 693.2031 | -0.4 | 485.1455, 337.0932, 307.0831, 193.0513, 177.0569, 175.0403, 163.0398, 145.0306 | O-feruloyl-O-p-coumaroyl-rhamnose-glucoside | Phenolic acids |
| 98 | 7.81 | C_16_H_14_N_2_O_5_ | [M+H]+ | 315.0974 | 315.0981 | -0.7 | 269.0922, 225.1024, 223.0869, 167.0130, 141.9593, 125.9868 | carboxyl-cuscutamine# | Alkaloids |
|  |  |  | [M-H]- | 313.0831 | 313.0824 | 0.7 | 269.0938, 225.1041, 183.0929, 156.0822 |  |  |
| 99 | 7.81 | C_25_H_26_O_11_ | [M-H]- | 501.1405 | 501.1397 | 0.8 | 337.0935, 323.0762, 193.0510, 175.0404, 163.0405, 119.0505 | O-feruloyl-O-p-coumaroyl-β-D-glucopyranoside | Phenolic acids |
| 100 | 7.99 | C_28_H_24_O_13_ | [M-H]- | 567.1128 | 567.1154 | -2.6 | 477.0970, 285.0415, 284.0328, 255.0315, 227.0350, 151.0024 | Kaempferol-O-p-hydroxybenzoylglucoside | Flavonols |
| 101 | 8.03 | C_30_H_26_O_14_ | [M-H]- | 609.125 | 609.1244 | 0.6 | 447.0907, 323.0777, 285.0415, 284.0335, 255.0298, 227.0322, 179.0359, 161.0248, 135.0459 | Kaempferol O-(caffeoyl)-β-D-glucopyranoside | Flavonols |
| 102 | 8.2 | C_25_H_24_O_11_ | [M-H]- | 499.1241 | 499.124 | 0.1 | 337.0953, 179.0359, 173.0466, 163.0405, 119.0497 | O-caffeoyl-O-p-coumaroyl quinic acid | Phenolic acids |
| 103 | 8.32 | C_28_H_24_O_13_ | [M-H]- | 567.1129 | 567.1139 | -1 | 285.0400, 284.0336, 255.0321, 227.0338, 137.0251 | Kaempferol-O-p-hydroxybenzoylglucoside | Flavonols |
| 104 | 8.32 | C_30_H_26_O_14_ | [M-H]- | 609.1238 | 609.1244 | -0.6 | 447.0936, 323.0800, 285.0408, 284.0305, 255.0298, 227.0350, 179.0370, 161.0248, 135.0446 | Kaempferol O-(caffeoyl)-β-D-glucopyranoside | Flavonols |
| 105 | 8.34 | C_15_H_14_N_2_O_3_ | [M+H]+ | 271.1083 | 271.1083 | 0 | 225.1027, 197.1081, 180.0816, 169.0773, 130.0660 | Cuscutamine isomer | Alkaloids |
|  |  |  | [M-H]- | 269.0931 | 269.0926 | 0.5 | 225.1038, 183.0932, 166.0666, 156.0825 |  |  |
| 106 | 8.4 | C_26_H_26_O_12_ | [M-H]- | 529.1351 | 529.1346 | 0.5 | 367.1025, 193.0511, 173.0466, 134.0384 | O-Feruloyl-O-caffeoylquinic acid | Phenolic acids |
| 107 | 8.44 | C_37_H_46_O_21_ | [M+HCOO]- | 871.2501 | 871.2508 | -0.7 | 825.2449, 663.1931, 369.0981, 323.0983, 311.0931, 263.0765, 221.0671, 219.0658, 191.0566, 179.0556, 161.0462, 125.0247, 119.0346, 101.0250, 99.0087 | Cuscutoside D | Lignans |
| 108 | 8.54 | C_26_H_26_O_12_ | [M-H]- | 529.1342 | 529.1346 | -0.4 | 367.1103, 353.0876, 193.0485, 191.0555, 179.0358, 173.0462, 161.0269, 135.0450 | O-Feruloyl-O-caffeoylquinic acid | Phenolic acids |
| 109 | 8.58 | C_30_H_26_O_14_ | [M-H]- | 609.1247 | 609.1244 | 0.3 | 463.0885, 301.0341, 300.0277, 271.0249, 255.0311, 151.0031 | Quercetin-3-O-coumaroylgalactoside | Flavonols |
| 110 | 8.64 | C_39_H_40_O_17_ | [M-H]- | 779.2184 | 779.2187 | -0.3 | 633.1850, 615.1743, 471.1298, 307.0834, 205.0526, 163.0408, 145.0302, 119.0510 | Tricoumaroyl-di-β-D-glucopyranoside | Phenolic acids |
| 111 | 8.76 | C_31_H_28_O_15_ | [M-H]- | 639.1346 | 639.135 | -0.4 | 463.0905, 370.0356, 301.0349, 271.0232, 255.0307, 151.0034 | Quercetin 3-O-(6''-feruloyl)-β-D-galactopyranoside | Flavonols |
| 112 | 8.81 | C_15_H_14_N_2_O_3_ | [M+H]+ | 271.1084 | 271.1083 | 0.1 | 225.1026, 197.1071, 180.0812, 169.0770, 130.0658 | Cuscutamine* | Alkaloids |
|  |  |  | [M-H]- | 269.0938 | 269.0926 | 1.2 | 225.1040, 183.0932, 166.0666, 156.0822 |  |  |
| 113 | 8.98 | C_36_H_44_O_20_ | [M+HCOO]- | 841.2415 | 841.2402 | 1.3 | 795.2363, 369.0994 | Neocuscutoside B | Lignans |
| 114 | 9.03 | C_15_H_10_O_7_ | [M+H]+ | 303.0502 | 303.0505 | -0.3 | 257.0439, 229.0506, 201.0549, 153.0186, 137.0236 | Quercetin* | Flavonols |
|  |  | C_15_H_10_O_7_ | [M-H]- | 301.0356 | 301.0348 | 0.8 | 273.0414, 178.9988, 151.0038, 121.0295, 107.0141 |  |  |
| 115 | 9.15 | C_30_H_26_O_13_ | [M-H]- | 593.1299 | 593.1295 | 0.4 | 447.0938, 285.0389, 284.0328, 255.0286, 227.0367, 163.0362, 145.0299 | Tiliroside (Kaempferol-3-O-β-D-(6''-trans-p-coumaroyl) glucopyranoside) | Flavonols |
| 116 | 9.19 | C_28_H_36_O_10_ | [M-H]- | 531.2237 | 531.223 | 0.7 | 163.0405, 145.0313, 119.0498, 117.0349 | C_19_H_28_O_7_+coumaric acid# | Phenolic acids |
| 117 | 9.25 | C_32_H_38_O_17_ | [M+HCOO]- | 739.2086 | 739.2086 | 0 | 369.0988, 340.0944, 323.0988, 311.0973, 263.0803, 221.0673, 219.0644, 191.0731, 179.0563, 161.0462, 125.0240, 119.0364, 113.0274, 101.0239 | Sesaminol diglucoside* | Lignans |
| 118 | 9.35 | C_30_H_26_O_13_ | [M-H]- | 593.1295 | 593.1295 | 0 | 447.0933, 285.0398, 284.0326, 255.0296, 227.0349, 151.0043, 145.0307 | Tiliroside isomer (Kaempferol-3-O-β-D-(6''-trans-p-coumaroyl) glucopyranoside) | Flavonols |
| 119 | 9.39 | C_25_H_24_O_10_ | [M-H]- | 483.1301 | 483.1291 | 1 | 337.0944, 191.0554, 173.0454, 163.0482, 119.0523 | Di-O-p-coumaroylquinic acid | Phenolic acids |
| 120 | 9.63 | C_26_H_26_O_11_ | [M-H]- | 513.1397 | 513.1397 | 0 | 367.1033, 193.0502, 173.0453 | O-p-coumaroyl-O-feruloylquinic acid | Phenolic acids |
| 121 | 9.75 | C_31_H_36_O_16_ | [M+HCOO]- | 709.1973 | 709.198 | -0.7 | 663.1945, 369.0982, 340.0956, 311.0904, 293.0879, 219.0667, 191.0688 | Cuscutoside A | Lignans |
| 122 | 10.35 | C_31_H_36_O_16_ | [M+HCOO]- | 709.1988 | 709.198 | 0.8 | 663.1924, 369.0976, 340.0953, 307.0830, 219.0672, 191.0578, 161.0405, 145.0292 | Cuscutoside B | Lignans |
| 123 | 10.44 | C_36_H_54_O_17_ | [M+H]+ | 759.3428 | 759.3439 | -1.1 | 369.2411, 351.2201, 163.0399 | C_20_H_36_O_8_+caffeoylquinic acid# | Fatty acids/Phenolic acids |
|  |  |  | [M-H]- | 757.3287 | 757.3283 | 0.4 | 353.0888, 191.0567, 179.0352, 173.0469, 161.0246, 135.0454 |  |  |
| 124 | 10.52 | C_35_H_38_O_13_ | [M-H]- | 665.2249 | 665.2234 | 1.5 | 307.0837, 187.0393, 163.0405, 145.0298, 119.0516, 117.0349 | C_35_H_38_O_13_# | Phenolic acids |
| 125 | 10.79 | C_15_H_10_O_6_ | [M+H]+ | 287.0554 | 287.0556 | 0.2 | 258.0523, 213.0527, 165.0182, 153.0184, 121.0282 | Kaempferol* | Flavonols |
|  |  | C_15_H_10_O_6_ | [M-H]- | 285.0411 | 285.0399 | 1.2 | 239.0358 |  |  |
| 126 | 11.16 | C_16_H_12_O_7_ | [M+H]+ | 317.0676 | 317.0661 | 1.6 | 302.0445, 274.0477, 229.0515, 187.0889, 153.0200 | Isorhamnetin* | Flavonols |
|  |  | C_16_H_12_O_7_ | [M-H]- | 315.0510 | 315.0505 | 0.5 | 300.0285, 283.0271, 271.0265, 255.0314, 243.0317, 227.0352 |  |  |
| 127 | 11.28 | C_43_H_74_O_23_ | [M+NH_4_]+ | 976.4943 | 976.4965 | -2.2 | 393.1805, 375.1671, 257.1006, 247.1179, 225.1857, 207.1739, 189.1167, 111.0484 | Unknown# | Resin glycosides |
|  |  |  | [M+HCOO]- | 1003.4610 | 1003.4597 | 1.3 | 939.4430, 913.4293, 857.4027, 839.3917, 711.3467, 693.3366, 565.2869, 547.2750, 403.2343, 337.2043 |  |  |
| 128 | 11.42 | C_18_H_36_O_5_ | [M-H]- | 327.218 | 327.2171 | 0.9 | 219.1986, 239.1299, 229.1451, 221.1349, 183.1407, 171.1032 | Trihydroxyoctadecanoic acid | Fatty acids |
| 129 | 11.83 | C_13_H_25_NO_3_ | [M-H]- | 242.1769 | 242.1756 | 1.3 | 225.1505, 181.1606 | N-Undecanoylglycine | Fatty acids |
| 130 | 12.31 | C_42_H_72_O_22_ | [M+NH_4_]+ | 946.4854 | 946.4859 | -0.5 | 393.1721, 375.1662, 257.1044, 247.1197, 225.1854, 207.1753, 189.1653, 111.0450 | rhamnose+6-O-Acetyl-D-glucosyl+rhamnosyl+rhamnosyl+11-Hydroxytetradecanoic acid+acetyl# | Resin glycosides |
|  |  |  | [M+HCOO]- | 973.4502 | 973.4492 | 1.0 | 909.4375, 883.4206, 827.3908, 809.3813, 681.3386, 663.3314, 535.2753, 517.2720, 469.2461, 373.2232, 337.2027 |  |  |
| 131 | 12.47 | C_18_H_34_O_5_ | [M-H]- | 329.2332 | 329.2328 | 0.4 | 229.1447, 211.1345, 183.1394, 171.1035, 139.1140 | Trihydroxyoctadecenoic acid | Fatty acids |
| 132 | 12.52 | C_42_H_66_O_22_ | [M-H]- | 921.3969 | 921.3967 | 0.2 | 713.3397, 487.1455, 307.0831, 265.0718, 235.0615, 205.0514, 163.0412, 145.0299 | glucose+6-O-Acetyl-D-glucosyl+1-deoxy-1-[(4-methoxyphenyl)amino]-D-glucitol+1-deoxy-1-[(4-methoxyphenyl)amino]-D-glucitol# | Resin glycosides |
| 133 | 12.62 | C_18_H_34_O_5_ | [M-H]- | 329.2339 | 329.2328 | 1.1 | 229.1471, 211.1353, 183.1404, 171.1031, 139.1134 | Trihydroxyoctadecenoic acid | Fatty acids |
| 134 | 12.75 | C_37_H_66_O_18_ | [M+H]+ | 799.4312 | 799.4327 | -1.5 | 781.4206, 763.4079, 619.3680, 601.3574, 583.3474, 473.3102, 455.3005, 437.2892, 419.1930, 355.2498, 337.2389, 319.2237, 227.2008, 209.1941, 191.1806, 111.0445 | Cus 3 isomer | Resin glycosides |
|  |  |  | [M+HCOO]- | 843.4225 | 843.4226 | -0.1 | 659.3639, 633.3501, 243.1973, 205.0724, 163.0619, 117.0556 |  |  |
| 135 | 12.84 | C_32_H_58_O_16_ | [M+NH_4_]+ | 716.4036 | 716.4069 | -3.3 | 391.2336, 373.2482, 255.0804, 239.0852, 227.1999, 209.1900, 147.0632, 129.0553, 111.0432 | Cuscutic acid A2 | Resin glycosides |
|  |  |  | [M-H]- | 697.3648 | 697.3647 | 0.1 | 551.3069, 533.2987, 389.2547, 371.2447, 243.1982, 225.1864 |  |  |
| 136 | 12.86 | C_16_H_32_O_4_ | [M-H]- | 287.2230 | 287.2222 | 0.8 | 269.2097, 243.2044 | Dihydroxyhexadecanoic acid | Fatty acids |
| 137 | 12.86 | C_36_H_62_O_18_ | [M+HCOO]- | 827.3904 | 827.3913 | -0.9 | 737.3560, 681.3345, 663.3223, 535.2756, 469.2451, 373.2225, 337.2032, 243.1950 | rhamnose+6-O-Acetyl-D-glucosyl+rhamnosyl+11-Hydroxytetradecanoic acid+acetyl# | Resin glycosides |
| 138 | 12.89 | C_43_H_74_O_22_ | [M+NH_4_]+ | 960.4987 | 960.5015 | -2.8 | 393.1764, 375.1633, 275.1117, 257.0998, 247.1178, 225.1846, 207.1752, 189.1632, 111.0453 | xylose+6-O-Acetyl-D-glucosyl+rhamnosyl+rhamnosyl+11-Hydroxyhexadecanoic acid+acetyl# | Resin glycosides |
|  |  |  | [M+HCOO]- | 987.4654 | 987.4648 | 0.6 | 923.4451, 897.4341, 841.4069, 823.3967, 695.3511, 549.2909, 531.2810, 483.2597, 387.2388 |  |  |
| 139 | 12.95 | C_36_H_54_O_16_ | [M+H]+ | 743.3457 | 743.349 | -3.3 | 371.2409, 353.2286, 335.2230, 317.2126, 163.0392 | C_20_H_36_O_7_+caffeoylquinic acid# | Fatty acids/Pbenolic acids |
|  |  |  | [M-H]- | 741.3337 | 741.3334 | 0.3 | 353.0879, 191.0563, 179.0350, 135.0464 |  |  |
| 140 | 12.98 | C_16_H_32_O_4_ | [M-H]- | 287.2235 | 287.2222 | 1.1 | 269.2094, 243.2043 | Dihydroxyhexadecanoic acid | Fatty acids |
| 141 | 13.07 | C_32_H_58_O_16_ | [M-H]- | 697.364 | 697.3647 | -0.7 | 551.3068, 533.2964, 389.2553, 371.2453, 243.1981, 225.1842 | Cuscutic acid A2 isomer | Resin glycosides |
| 142 | 13.1 | C_37_H_66_O_18_ | [M+H]+ | 799.4286 | 799.4327 | -4.1 | 781.4183, 763.4051, 619.3660, 601.3556, 583.3464, 473.3089, 455.2986, 437.2885, 419.1925, 355.2477, 337.2386, 319.2268, 227.2009, 209.1900, 191.1790, 111.0446 | Cus 3 | Resin glycosides |
|  |  |  | [M+HCOO]- | 843.4219 | 843.4226 | -0.7 | 633.3476, 577.3259, 407.1578, 351.1309, 243.1978, 205.0726, 163.0608, 117.0554 |  |  |
| 143 | 13.27 | C_43_H_74_O_22_ | [M+HCOO]- | 987.4659 | 987.4648 | 1.1 | 923.4352, 897.4367, 841.4130, 823.3934, 695.3426, 549.2922, 531.2842, 483.2584, 387.2375 | xylose+6-O-Acetyl-D-glucosyl+rhamnosyl+rhamnosyl+11-Hydroxyhexadecanoic acid+acetyl# | Resin glycosides |
| 144 | 13.44 | C_37_H_66_O_18_ | [M+HCOO]- | 843.4214 | 843.4226 | -1.2 | 659.3639, 633.3501, 243.1973, 205.0701, 163.0603, 117.0558 | Cus 3 isomer | Resin glycosides |
| 145 | 13.7 | C_18_H_34_O_5_ | [M-H]- | 329.234 | 329.2328 | 1.2 | 201.1133, 199.1370, 171.1027 | Trihydroxyoctadecenoic acid | Fatty acids |
| 146 | 13.79 | C_38_H_66_O_19_ | [M+H]+ | 827.4257 | 827.4277 | -2 | 441.2857, 423.2710, 337.2316, 319.1162, 209.1906, 191.1802, 111.0447 | rhamnose+6-O-Acetyl-D-glucosyl+6-O-Acetyl-D-glucosyl+11-Hydroxyhexadecanoic acid# | Resin glycosides |
|  |  |  | [M+HCOO]- | 871.418 | 871.4175 | 0.5 | 645.3460, 619.3329, 577.3229, 559.3105, 351.1299, 243.1970, 205.0717, 187.0605, 163.0617, 145.0508, 143.0345, 103.0398 |  |  |
| 147 | 14.18 | C_39_H_68_O_19_ | [M+H]+ | 841.4423 | 841.4433 | -1 | 601.3577, 583.3480, 473.3101, 455.3003, 437.2893, 355.2480, 337.2377, 327.2533, 319.2273, 227.2011, 209.1905, 191.1799 | Cus 1 isomer | Resin glycosides |
|  |  | C_39_H_68_O_19_ | [M+HCOO]- | 885.4358 | 885.4331 | 2.7 | 659.3651, 633.3492, 577.3232, 559.3132, 407.1564, 351.1299, 333.1195, 315.1089, 243.1973, 205.0719, 187.0614, 163.0611, 143.0351, 117.0557 |  |  |
| 148 | 14.30 | C_36_H_62_O_18_ | [M+H]+ | 783.4009 | 783.4014 | -0.5 | 765.3906, 747.3801, 729.3654, 637.3437, 619.3356, 601.3450, 561.3278, 543.3162, 525.3067, 415.2686, 397.2583, 355.2504, 337.2468, 319.2282, 209.1902, 111.0447 | Cus 4 isomer | Resin glycosides |
|  |  |  | [M+HCOO]- | 827.3908 | 827.3913 | -0.5 | 619.3341, 577.3226, 559.3138, 393.1393, 351.1293, 333.1203, 285.2076, 257.2122, 243.1972, 223.0825, 205.0730, 187.0614, 163.0607, 143.0349, 115.0394, 103.0399 |  |  |
| 149 | 14.48 | C_39_H_68_O_19_ | [M+H]+ | 841.4413 | 841.4433 | -2 | 601.3597, 583.3496, 473.3108, 455.2999, 355.2494, 337.2372, 319.2273, 301.2155, 227.2002, 209.1904, 191.1803 | Cus 1 isomer | Resin glycosides |
|  |  | C_39_H_68_O_19_ | [M+HCOO]- | 885.4334 | 885.4331 | 0.3 | 659.3725, 633.3529, 243.1968, 163.0610, 117.0555 |  |  |
| 150 | 14.54 | C_38_H_66_O_19_ | [M+H]+ | 827.4248 | 827.4277 | -2.9 | 441.2857, 423.2710, 337.2316, 319.1162, 209.1906, 191.1802, 111.0447 | rhamnose+6-O-Acetyl-D-glucosyl+6-O-Acetyl-D-glucosyl+11-Hydroxyhexadecanoic acid# | Resin glycosides |
|  |  |  | [M+HCOO]- | 871.4175 | 871.4175 | 0 | 645.3500, 619.3336, 577.3227, 559.3110, 351.1297, 243.1971, 205.0719, 187.0608, 163.0612, 145.0506, 143.0348, 103.0401 |  |  |
| 151 | 14.83 | C_32_H_58_O_16_ | [M-H]- | 697.3651 | 697.3647 | 0.4 | 487.3006, 299.2263, 285.2100, 257.2115, 243.1969, 117.0556 | rhamnose+galactosyl+rhamnosyl+11-Hydroxytetradecanoic acid# | Resin glycosides |
| 152 | 14.89 | C_39_H_68_O_19_ | [M+H]+ | 841.4415 | 841.4433 | -1.8 | 601.3573, 583.3477, 473.3101, 455.3002, 437.2901, 411.2741, 355.2480, 337.2376, 319.2273, 227.2010, 209.1905, 191.1798 | Cus 1 | Resin glycosides |
|  |  | C_39_H_68_O_19_ | [M+HCOO]- | 885.4402 | 885.4331 | 7.1 | 659.3653, 633.3496, 577.3234, 559.3123, 433.1719, 407.1562, 351.1300, 333.1195, 243.1973, 205.0719, 187.0615, 163.0610, 143.0348, 117.0557, 99.045 |  |  |
| 153 | 15.00 | C_36_H_62_O_18_ | [M+H]+ | 783.3998 | 783.4014 | -1.6 | 765.3893, 747.3790, 729.3696, 637.3435, 619.3361, 601.3454, 561.3263, 543.3158, 525.3050, 415.2686, 397.2583, 355.2472, 337.2384, 319.2271, 209.1910, 111.0447 | Cus 4 | Resin glycosides |
|  |  |  | [M+HCOO]- | 827.3928 | 827.3913 | 1.5 | 619.3335, 577.3245, 559.3118, 393.1409, 351.1299, 333.1199, 285.2085, 257.2133, 243.1970, 223.0819, 205.0726, 187.0620, 163.0612, 143.0345, 115.0397, 103.0406 |  |  |
| 154 | 15.31 | C_39_H_66_O_19_ | [M+NH_4_]+ | 856.4536 | 856.4542 | -0.6 | 471.2943, 427.2715, 353.2313, 325.2373, 243.1955, 225.1860, 111.0447 | rhamnose+6-O-Acetyl-D-glucosyl+rhamnosyl+3-Hydroxy-2-methylbutanasyl+Hydroxytetradecenoic acid# | Resin glycosides |
|  |  |  | [M+HCOO]- | 883.4185 | 883.4175 | 1 | 657.3493, 631.3364, 575.3065, 557.2943, 433.1738, 407.1561, 351.1302, 333.1208, 241.1819, 205.0727, 163.0616, 117.0559 |  |  |
| 155 | 15.38 | C_43_H_72_O_21_ | [M+H]+ | 925.4651 | 925.4644 | 0.7 | 599.3459, 455.2994, 437.2891, 371.2418, 353.2296, 209.1924, 177.0258, 127.0388, 111.0453 | rhamnose+6-O-Acetyl-D-glucosyl+rhamnosyl+3-Hydroxy-2-methylbutanasyl+11-Hydroxytetradecanoic acid+acetyl+acetyl# | Resin glycosides |
|  |  |  | [M+HCOO]- | 969.4562 | 969.4543 | 1.9 | 633.3517, 559.3116, 407.1596, 351.1305, 243.1977, 163.0617, 117.0561 |  |  |
| 156 | 15.56 | C_43_H_74_O_22_ | [M+NH_4_]+ | 960.5029 | 960.5015 | 1.4 | 473.3091, 455.3037, 437.2979, 355.2455, 337.2395, 319.2280, 227.2011, 209.1920, 111.0460 | rhamnose+6-O-Acetyl-D-glucosyl+rhamnosyl+3-Hydroxy-2-methylbutanasyl+11-Hydroxytetradecanoic acid+acetyl+acetyl+H_2_O# | Resin glycosides |
|  |  |  | [M-H]- | 941.4593 | 941.4593 | 0 | 923.4473, 779.4046, 761.4106, 735.3824, 679.3568, 647.3304, 455.2655, 345.2287, 327.2180, 269.2128, 243.1978, 225.1887, 117.0559 |  |  |
| 157 | 15.67 | C_43_H_72_O_21_ | [M+H]+ | 925.4648 | 925.4644 | 0.4 | 599.3401, 581.3430, 455.2982, 437.3954, 353.2350, 209.1904, 145.0508, 127.0399, 111.0443 | rhamnose+6-O-Acetyl-D-glucosyl+rhamnosyl+3-Hydroxy-2-methylbutanasyl+11-Hydroxytetradecanoic acid+acetyl+acetyl# | Resin glycosides |
|  |  |  | [M+HCOO]- | 969.4567 | 969.4543 | 0.4 | 659.3604, 633.3497, 351.1299, 243.1974, 205.0708, 163.0609, 117.0558 |  |  |
| 158 | 15.81 | C_38_H_66_O_18_ | [M+H]+ | 811.4322 | 811.4327 | -0.5 | 793.4230, 775.4119, 589.3593, 571.3488, 553.3382, 443.3015, 425.2911, 355.2530, 337.2380, 319.2284, 227.2035, 209.1913, 199.0977, 191.1802, 111.0452, 83.0498 | rhamnose+Arabinopyranosyl+galactosyl+rhamnosyl+11-Hydroxytetradecanoic acid+acetyl# | Resin glycosides |
|  |  |  | [M+HCOO]- | 855.424 | 855.4226 | 1.4 | 647.3638, 559.3128, 421.1724, 351.1286, 333.1205, 313.2400, 285.2081, 243.1975, 205.0725, 187.0623, 163.0612, 143.0352, 87.0451 |  |  |
| 159 | 15.92 | C_33_H_58_O_15_ | [M+HCOO]- | 739.3773 | 739.3752 | 2.1 | 693.3712, 487.2906, 299.2239, 285.2076, 257.2131, 243.1977, 159.0658, 117.0555 | rhamnose+6-O-Acetyl-D-glucosyl+3-Hydroxy-2-methylbutanasyl+11-Hydroxytetradecanoic acid# | Resin glycosides |
| 160 | 16.01 | C_40_H_70_O_19_ | [M+HCOO]- | 899.4494 | 899.4488 | 0.6 | 619.3264, 577.3322, 351.1306, 243.1976, 205.0709, 187.0611, 163.0621, 143.0366, 131.0718 | rhamnosyl+6-O-Acetyl-D-glucosyl+rhamnosyl+3-Hydroxy-2-methylbutanasyl+11-Hydroxytetradecanoic acid# | Resin glycosides |
| 161 | 16.15 | C_40_H_70_O_19_ | [M+HCOO]- | 899.4495 | 899.4488 | 0.7 | 673.3839, 647.3668, 573.3301, 351.1300, 257.2126, 205.0701, 163.0612, 117.0561 | rhamnosyl+6-O-Acetyl-D-glucosyl+rhamnosyl+3-Hydroxy-2-methylbutanasyl+11-Hydroxytetradecanoic acid# | Resin glycosides |
| 162 | 16.19 | C_43_H_74_O_22_ | [M+NH_4_]+ | 960.5029 | 960.5015 | 1.4 | 601.3530, 525.3078, 473.3095, 455.3027, 437.2971, 355.2451, 337.2391, 319.2279, 227.2009, 209.1919, 111.0458 | rhamnose+6-O-Acetyl-D-glucosyl+rhamnosyl+3-Hydroxy-2-methylbutanasyl+11-Hydroxytetradecanoic acid+acetyl+acetyl+H_2_O# | Resin glycosides |
|  |  |  | [M-H]- | 941.46 | 941.4593 | 0.7 | 923.4318, 779.4069, 761.3958 735.3799, 679.3592, 647.3312, 455.2655, 345.2282, 327.2188, 269.2199 243.1969, 225.1868, 117.0559 |  |  |
| 163 | 16.32 | C_33_H_58_O_15_ | [M+HCOO]- | 739.3762 | 739.3752 | 1 | 487.2920, 299.2238, 285.2088, 257.2142, 243.1977, 187.0625, 159.0659, 117.0562, 73.0290 | rhamnose+6-O-Acetyl-D-glucosyl+3-Hydroxy-2-methylbutanasyl+11-Hydroxytetradecanoic acid# | Resin glycosides |
| 164 | 16.35 | C_44_H_72_O_22_ | [M+HCOO]- | 997.4485 | 997.4492 | -0.7 | 393.1365, 351.1266, 285.2066, 243.1980, 205.0720, 187.0624, 163.0614, 143.0351, 103.0396 | Unknown# | Resin glycosides |
| 165 | 16.39 | C_38_H_66_O_18_ | [M+H]+ | 811.4335 | 811.4327 | 0.8 | 793.4230, 775.4119, 589.3593, 571.3488, 553.3382, 443.3015, 425.2911, 355.2530, 337.2380, 319.2284, 227.2035, 209.1913, 199.0977, 191.1802, 111.0452, 83.0498 | rhamnose+Arabinopyranosyl+galactosyl+rhamnosyl+11-Hydroxytetradecanoic acid+acetyl# | Resin glycosides |
|  |  |  | [M+HCOO]- | 855.4227 | 855.4226 | 0.1 | 647.3651, 559.3135, 421.1722, 351.1293, 333.1198, 313.2396, 285.2066, 243.1971, 205.0717, 187.0614, 163.0614, 87.0450 |  |  |
| 166 | 16.68 | C_45_H_74_O_22_ | [M+H]+ | 967.4739 | 967.475 | -1.1 | 821.4152, 803.4045, 785.3939, 745.3987, 727.3881, 709.3785, 599.3419, 581.3312, 563.3224, 481.2795, 473.3110, 463.2685, 455.3004, 445.2591, 437.2893, 371.2429, 353.2323, 337.2375, 319.2269, 237.0764, 227.2012, 209.1905, 191.1802, 177.0553, 145.0502, 127.0397, 111.0447 | Unknown# | Resin glycosides |
|  |  |  | [M+HCOO]- | 1011.4662 | 1011.4648 | 1.4 | 659.3656, 633.3499, 577.3238, 559.3136, 433.1724, 407.1567, 351.1300, 333.1201, 243.1974, 205.0724, 187.0618, 163.0613, 117.0558 |  |  |
| 167 | 16.86 | C_41_H_72_O_19_ | [M+H]+ | 869.4745 | 869.4746 | -0.1 | 851.4605, 833.4497, 847.3972, 629.3871, 611.3768, 501.3383, 483.3305, 465.3127, 457.3108, 439.3056, 383.2789, 365.2685, 347.2575, 329.2484, 255.2326, 237.2213, 219.2105, 111.0450, 83.0497 | Cus 2 isomer | Resin glycosides |
|  |  |  | [M+HCOO]- | 913.4683 | 913.4644 | 3.9 | 687.3990, 661.3807, 587.3420, 433.1719, 407.1579, 351.1313, 333.1197, 271.2291, 205.0723, 187.0621, 163.0618, 117.0559, 99.0458 |  |  |
| 168 | 16.88 | C_14_H_28_O_3_ | [M-H]- | 243.1971 | 243.196 | 1.1 | 225.1868, 197.1920, 155.1428 | Hydroxytetradecanoic acid | Fatty acids |
| 169 | 16.88 | C_42_H_70_O_21_ | [M+HCOO]- | 953.4242 | 953.423 | 1.2 | 351.1307, 285.2078, 243.1974, 223.0818, 205.0705, 163.0612 | xylose+6-O-Acetyl-D-glucosyl+rhamnosyl+3-Hydroxy-2-methylbutanasyl+11-Hydroxytetradecanoic acid+acetyl+acetyl# | Resin glycosides |
| 170 | 16.96 | C_44_H_72_O_22_ | [M+HCOO]- | 997.4512 | 997.4492 | 2 | 351.1299, 333.1177, 285.2077, 243.1974, 205.0722, 187.0621, 163.0618, 143.0354, 103.0403 | Unknown# | Resin glycosides |
| 171 | 17.05 | C_41_H_72_O_19_.. | [M+HCOO]- | 913.4655 | 913.4644 | 1.1 | 351.1313, 271.2286, 205.0734, 163.0609, 117.0549 | Cus 2 isomer | Resin glycosides |
| 172 | 17.29 | C_45_H_74_O_22_ | [M+H]+ | 967.474 | 967.475 | -1 | 821.4152, 803.4045, 785.3939, 745.3987, 727.3881, 709.3785, 599.3419, 581.3312, 563.3224, 481.2795, 473.3110, 463.2685, 455.3004, 445.2591, 437.2893, 371.2429, 353.2323, 337.2375, 319.2269, 237.0764, 227.2012, 209.1905, 191.1802, 177.0553, 145.0502, 127.0397, 111.0447 | Unknown# | Resin glycosides |
|  |  |  | [M+HCOO]- | 1011.4681 | 1011.4648 | 3.3 | 659.3663, 633.3499, 577.3241, 559.3132, 433.1724, 407.1567, 351.1305, 333.1196, 243.1975, 205.0723, 187.0621, 163.0611, 117.0558 |  |  |
| 173 | 17.44 | C_41_H_72_O_19_.. | [M+H]+ | 869.4706 | 869.4746 | -4.1 | 851.4605, 833.4497, 847.3972, 629.3871, 611.3768, 501.3383, 483.3305, 465.3127, 457.3108, 439.3056, 383.2789, 365.2685, 347.2575, 329.2484, 255.2326, 237.2213, 219.2105, 111.0450, 83.0497 | Cus 2 | Resin glycosides |
|  |  |  | [M+HCOO]- | 913.4647 | 913.4644 | 0.3 | 687.3951, 661.3805, 587.3431, 433.1715, 407.1557, 351.1298, 333.1191, 271.2285, 205.0722, 187.0615, 163.0610, 117.0556, 99.0452 |  |  |
| 174 | 17.47 | C_42_H_70_O_21_ | [M+HCOO]- | 953.4239 | 953.423 | 0.9 | 619.3330, 577.3299, 351.1305, 285.2074, 243.1974, 223.0826, 205.0712, 163.0614 | xylose+6-O-Acetyl-D-glucosyl+rhamnosyl+3-Hydroxy-2-methylbutanasyl+11-Hydroxytetradecanoic acid+acetyl+acety# | Resin glycosides |
| 175 | 18.1 | C_18_H_34_O_4_ | [M-H]- | 313.239 | 313.2379 | 1.1 | 201.1127 | Methyl palmitoleate | Fatty acids |
| 176 | 18.21 | C_44_H_72_O_21_ | [M+H]+ | 937.4637 | 937.4644 | -0.7 | 569.3467, 551.3137, 533.3098, 495.2807, 481.2757, 463.2680, 443.3054, 425.2864, 371.2415, 353.2355, 337.2396, 319.2289, 301.2148, 283.2313, 255.0872, 237.0781, 219.0653, 209.1904, 199.0979, 177.0564, 145.0496, 127.0400, 111.0454 | Unknown# | Resin glycosides |
|  |  |  | [M+HCOO]- | 981.4546 | 981.4543 | 0.3 | 647.3658, 559.3118, 421.1721, 351.1317, 243.1971, 205.0722, 163.0612, 145.0499, 127.0400, 87.0455 |  |  |
| 177 | 18.73 | C_44_H_72_O_21_ | [M+H]+ | 937.4628 | 937.4644 | -1.6 | 569.3466, 551.3188, 533.3153, 481.2853, 463.2652, 443.3008, 425.2909, 371.2431, 353.2322, 337.2379, 319.2260, 283.2274, 255.0889, 237.0750, 219.0654, 209.1905, 199.0976, 177.0557, 145.0504, 127.0396, 111.0448 | Unknown# | Resin glycosides |
|  |  |  | [M+HCOO]- | 981.4561 | 981.4543 | 1.8 | 647.3645, 559.3150, 421.1705, 389.2541, 371.2463, 351.1276, 333.1177, 315.1112, 243.1976, 205.0722, 187.0630, 163.0611, 143.0356, 125.0246, 103.0404, 87.0454 |  |  |
| 178 | 19.04 | C_47_H_78_O_22_ | [M+H]+ | 995.5038 | 995.5063 | -2.5 | 609.3692, 509.3165, 491.2964, 483.3300, 465.3267, 439.3120, 399.3654, 381.2601, 365.2769, 347.2644, 255.2316, 237.2218, 219.2112, 195.0679, 177.0559, 167.0688, 145.0504, 127.0397, 111.0450, 85.0292 | Unknown# | Resin glycosides |
|  |  |  | [M+HCOO]- | 1039.4971 | 1039.4961 | 1 | 745.3954, 687.3940, 661.3873, 587.3416, 433.1739, 407.1613, 351.1294, 271.2289, 205.0700, 163.0608, 117.0560 |  |  |
| 179 | 19.08 | C_26_H_48_NO_7_P | [M+H]+ | 518.3242 | 518.3247 | -0.5 | 335.2578, 184.0742, 125.0008, 86.0972 | LPC(18:3) isomer | Phospholipids |
| 180 | 19.56 | C_26_H_48_NO_7_P | [M+H]+ | 518.323 | 518.3247 | -1.7 | 500.3139, 335.2577, 184.0739, 125.0001, 104.1077, 86.0969 | LPC(18:3) | Phospholipids |
|  |  |  | [M+HCOO]- | 562.3149 | 562.3145 | 0.4 | 502.2939, 227.2185, 224.0699, 168.0432, 152.9957, 78.9588 |  |  |
| 181 | 19.59 | C_18_H_30_O_3_ | [M-H]- | 293.2133 | 293.2117 | 1.6 | 275.2004, 183.0129, 171.1033 | Oxo-octadecadienoic acid or Hydroxyoctadecatrienoic acid | Fatty acids |
| 182 | 19.63 | C_47_H_78_O_22_ | [M+H]+ | 995.5024 | 995.5063 | -3.9 | 609.3602, 509.3094, 491.2999, 483.3304, 465.3199, 439.3153, 399.3738, 381.2639, 365.2714, 347.2579, 255.2313, 237.2224, 219.2109, 195.0649, 177.0551, 167.0706, 145.0498, 127.0402, 111.0445, 85.0290 | Unknown# | Resin glycosides |
|  |  |  | [M+HCOO]- | 1039.499 | 1039.4961 | 2.9 | 745.4093, 687.3955, 661.3807, 587.3370, 433.1707, 407.1520, 351.1301, 271.2287, 205.0734, 163.0611, 117.0563 |  |  |
| 183 | 19.79 | C_18_H_30_O_3_ | [M-H]- | 293.213 | 293.2117 | 1.3 | 183.0125 | Oxo-octadecadienoic acid or Hydroxyoctadecatrienoic acid | Fatty acids |
| 184 | 19.79 | C_27_H_49_O_12_P | [M-H]- | 595.2893 | 595.2883 | 1 | 315.0496, 279.2339, 241.0121, 223.0013, 152.9960, 96.9701, 78.9590 | [2-hydroxy-3-[hydroxy-[2,3,4,5,6-pentahydroxycyclohexyl]oxyphosphoryl]oxypropyl] octadeca-9,12-dienoate | Others |
| 185 | 20.35 | C_27_H_49_O_12_P | [M-H]- | 595.2887 | 595.2883 | 0.4 | 415.2257, 315.0490, 279.2342, 241.0122, 223.0009, 152.9959, 96.9695, 78.9591 | [2-hydroxy-3-[hydroxy-[2,3,4,5,6-pentahydroxycyclohexyl]oxyphosphoryl]oxypropyl] octadeca-9,12-dienoate | Others |
| 186 | 20.43 | C_23_H_44_NO_7_P | [M+H]+ | 478.2919 | 478.2934 | -1.5 | 337.2735, 306.2808, 263.2330 | LPC (16:1) isomer | Phospholipids |
|  |  |  | [M-H]- | 476.278 | 476.2777 | 0.3 | 279.2336 |  |  |
| 187 | 20.45 | C_16_H_32_O_3_ | [M-H]- | 271.2284 | 271.2273 | 1.1 | 253.2253 | Hydroxyhexadecanoic acid | Fatty acids |
| 188 | 20.55 | C_26_H_50_NO_7_P | [M+H]+ | 520.3402 | 520.3403 | -0.1 | 502.3289, 337.2741, 184.0741, 166.0636, 125.0004, 86.0971 | LPC (18:2) isomer | Phospholipids |
|  |  |  | [M+HCOO]- | 564.3307 | 564.3301 | 0.6 | 504.3107, 279.2341, 242.0805 |  |  |
| 189 | 20.77 | C_25_H_49_O_12_P | [M-H]- | 571.2889 | 571.2883 | 0.6 | 315.0475, 255.2345, 241.0112, 223.0017, 152.9960, 96.9702, 78.9591 | [2-hydroxy-3-[hydroxy-[2,3,4,5,6-pentahydroxycyclohexyl]oxyphosphoryl]oxypropyl] hexadecanoate | Others |
| 190 | 20.84 | C_18_H_32_O_3_ | [M-H]- | 295.2282 | 295.2273 | 0.9 | 183.0127 | Hydroxyoctadecadienoic acid | Fatty acids |
| 191 | 20.88 | C_23_H_44_NO_7_P | [M+H]+ | 478.2911 | 478.2934 | -2.3 | 460.2812, 337.2734, 306.2792, 263.2368, 155.0105 | LPC (16:1) | Phospholipids |
|  |  |  | [M-H]- | 476.278 | 476.2777 | 0.3 | 279.2337 |  |  |
| 192 | 20.97 | C_26_H_50_NO_7_P | [M+H]+ | 520.3423 | 520.3403 | 2 | 502.3284, 337.2737, 184.0737, 166.0634, 125.0004, 104.1076, 86.0970 | LPC (18:2) | Phospholipids |
|  |  |  | [M+HCOO]- | 564.3306 | 564.3301 | 0.5 | 504.3091, 279.2337, 242.0808, 224.0698 |  |  |
| 193 | 21.13 | C_18_H_32_O_3_ | [M+H-H_2_O]+ | 279.2319 | 279.2324 | -0.5 | 201.0466, 173.1323, 159.1199, 149.1344 | Hydroxyoctadecadienoic acid | Fatty acids |
|  |  |  | [M-H]- | 295.2284 | 295.2273 | 1.1 | 277.2185, 195.1402, 183.0125, 171.1038 |  |  |
| 194 | 21.33 | C_25_H_49_O_12_P | [M-H]- | 571.288 | 571.2883 | -0.3 | 391.2263, 315.0487, 255.2338, 241.0124, 223.0008, 152.9959, 96.9692, 78.9589 | [2-hydroxy-3-[hydroxy-[2,3,4,5,6-pentahydroxycyclohexyl]oxyphosphoryl]oxypropyl] hexadecanoate | Others |
| 195 | 21.42 | C_24_H_50_NO_7_P | [M+H]+ | 496.339 | 496.3403 | -1.3 | 478.3242, 313.2787, 184.0736, 125.0003, 86.0971 | LPC (16:0) isomer | Phospholipids |
|  |  |  | [M+HCOO]- | 540.3319 | 540.3301 | 1.8 | 480.3106, 255.2348, 224.0741 |  |  |
| 196 | 21.51 | C_18_H_30_O_3_ | [M+H]+ | 295.2268 | 295.2273 | -0.5 | 277.2144, 235.1700, 201.0467 | Oxo-octadecadienoic acid or Hydroxyoctadecatrienoic acid | Fatty acids |
|  |  |  | [M-H]- | 293.2127 | 293.2117 | 1 | 183.0137 |  |  |
| 197 | 21.66 | C_18_H_30_O_3_ | [M+H]+ | 295.2269 | 295.2273 | -0.4 | 277.2158, 235.1609, 201.0471, 173.0526 | Oxo-octadecadienoic acid or Hydroxyoctadecatrienoic acid | Fatty acids |
|  |  |  | [M-H]- | 293.2128 | 293.2117 | 1.1 | 183.0137 |  |  |
| 198 | 21.78 | C_21_H_44_NO_7_P | [M+H]+ | 454.2915 | 454.2934 | -1.9 | 436.2814, 393.2411, 313.2737, 282.2802, 155.0140 | Lysophosphatidylethanolamine 16:0 | Phospholipids |
|  |  |  | [M-H]- | 452.2786 | 452.2777 | 0.9 | 255.2345, 196.0390, 140.0117 |  |  |
| 199 | 21.83 | C_18_H_30_O_3_ | [M+H]+ | 295.2267 | 295.2273 | -0.6 | 277.2168, 259.2037, 231.2106, 201.0464, 173.0518 | Oxo-octadecadienoic acid or Hydroxyoctadecatrienoic acid | Fatty acids |
|  |  |  | [M-H]- | 293.2133 | 293.2117 | 1.6 | 183.0117 |  |  |
| 200 | 21.83 | C_24_H_50_NO_7_P | [M+H]+ | 496.3397 | 496.3403 | -0.6 | 478.3292, 313.2739, 184.0737, 125.0005, 104.1075, 86.0969 | LPC (16:0)* | Phospholipids |
|  |  |  | [M+HCOO]- | 540.3311 | 540.3301 | 1 | 480.3103, 255.2339, 224.0700 |  |  |
| 201 | 22.1 | C_18_H_30_O_3_ | [M+H]+ | 295.2279 | 295.2273 | 0.6 | 277.2176, 259.2051, 201.0476, 173.0526, 149.0240 | Oxo-octadecadienoic acid or Hydroxyoctadecatrienoic acid | Fatty acids |
|  |  |  | [M-H]- | 293.213 | 293.2117 | 1.3 | 183.0137 |  |  |
| 202 | 22.31 | C_26_H_52_NO_7_P | [M+H]+ | 522.3563 | 522.356 | 0.3 | 504.3462, 339.2865, 184.0743, 166.0672, 125.0006, 104.1081, 86.0968 | LPC (18:1) | Phospholipids |
| 203 | 24.36 | C_16_H_32_O_3_ | [M-H]- | 271.2281 | 271.2273 | 0.8 | 225.2246 | Hydroxyhexadecanoic acid | Fatty acids |

*: Confirmed with reference standards

#: Unreported compounds

**Table S4.** Prediction accuracy of compounds by different software

|  | **Sirius** | | **Unifi** | | **MATLAB** | |
| --- | --- | --- | --- | --- | --- | --- |
| **Ion mode** | **Positive** | **Negative** | **Positive** | **Negative** | **Positive** | **Negative** |
| **Filtration of features** | 320 | 265 | 366 | 252 | 291 | 215 |
| **Identification of features** | 110 | 135 | 91 | 84 | 233 | 197 |
| **Accuracy rate** | 34.37% | 50.94% | 24.86% | 33.33% | 80.07% | 91.63% |

**Table S5.** The nodes and edges of various structural types with different α and β values in positive ion.

|  | **α = 1, β = 0** | | **α = 0.9, β = 0.1** | | **α = 0.8, β = 0.2** | | **α = 0.7, β = 0.3** | | **α = 0.6, β = 0.4** | | **α = 0.5, β = 0.5** | |
| --- | --- | --- | --- | --- | --- | --- | --- | --- | --- | --- | --- | --- |
|  | $\boldsymbol{S}\boldsymbol{imilarity}\boldsymbol{=}\boldsymbol{\alpha}\boldsymbol{\times}\boldsymbol{Sim}_{\boldsymbol{frag}\boldsymbol{ment}}\boldsymbol{+}\boldsymbol{\beta}\boldsymbol{\times}\boldsymbol{Sim}_{\boldsymbol{neutral loss}}\boldsymbol{\geq}\mathbf{0.6}$ | | | | | | | | | | | |
|  | **Nodes** | **Edges** | **Nodes** | **Edges** | **Nodes** | **Edges** | **Nodes** | **Edges** | **Nodes** | **Edges** | **Nodes** | **Edges** |
| **Phenolic acids** | 22 | 86 | 34 | 104 | 24 | 80 | 12 | 42 | 4 | 4 | 2 | 1 |
| **Flavonols** | 24 | 70 | 33 | 91 | 20 | 51 | 8 | 22 | 2 | 1 | 0 | 0 |
| **Resin glycosides** | 60 | 386 | 65 | 469 | 51 | 386 | 26 | 120 | 8 | 10 | 4 | 7 |
| **Fatty acids** | 24 | 51 | 23 | 49 | 12 | 30 | 6 | 15 | 6 | 10 | 2 | 1 |
| **Phospholipids** | 15 | 32 | 18 | 60 | 16 | 58 | 12 | 10 | 4 | 6 | 4 | 3 |
| **Alkaloids** | 7 | 15 | 7 | 14 | 4 | 6 | 4 | 4 | 2 | 1 | 2 | 1 |

**Table S6.** The nodes and edges of various structural types with different α and β values in negative ion.

|  | **α = 1, β = 0** | | **α = 0.9, β = 0.1** | | **α = 0.8, β = 0.2** | | **α = 0.7, β = 0.3** | | **α = 0.6, β = 0.4** | | **α = 0.5, β = 0.5** | |
| --- | --- | --- | --- | --- | --- | --- | --- | --- | --- | --- | --- | --- |
|  | $\boldsymbol{S}\boldsymbol{imilarity}\boldsymbol{=}\boldsymbol{\alpha}\boldsymbol{\times}\boldsymbol{Sim}_{\boldsymbol{frag}\boldsymbol{ment}}\boldsymbol{+}\boldsymbol{\beta}\boldsymbol{\times}\boldsymbol{Sim}_{\boldsymbol{neutral loss}}\boldsymbol{\geq}\mathbf{0.}\mathbf{6}$ | | | | | | | | | | | |
|  | **Nodes** | **Edges** | **Nodes** | **Edges** | **Nodes** | **Edges** | **Nodes** | **Edges** | **Nodes** | **Edges** | **Nodes** | **Edges** |
| **Phenolic acids** | 45 | 198 | 55 | 210 | 30 | 111 | 15 | 89 | 9 | 20 | 3 | 5 |
| **Flavonols** | 25 | 118 | 28 | 126 | 16 | 87 | 10 | 55 | 5 | 12 | 2 | 1 |
| **Resin glycosides** | 46 | 694 | 56 | 732 | 35 | 516 | 19 | 101 | 6 | 9 | 0 | 0 |
| **Fatty acids** | 8 | 8 | 8 | 9 | 6 | 5 | 4 | 3 | 3 | 2 | 0 | 0 |
| **Phospholipids** | 16 | 40 | 20 | 52 | 11 | 29 | 4 | 3 | 0 | 0 | 0 | 0 |
| **Alkaloids** | 5 | 6 | 6 | 7 | 4 | 3 | 2 | 1 | 2 | 1 | 0 | 0 |

**Table S7.** The regression equations and linear ranges of 9 analytes (n = 3) in rat plasma

| Analytes | t_R_(min) | LLOQ  (ng/ml) | Range | Linear regression equation (n=3) | Correlation cofficient(r) |
| --- | --- | --- | --- | --- | --- |
| Isorhamnetin-7-β-O-glucoside | 3.12 | 0.08 | 0.08-10.00 | Y=5.8205e^-2^X+7.8487e^-4^ | 0.9985 |
| Hyperoside | 2.67 | 0.39 | 0.39-49.98 | Y=1.3916e^-2^X+1.9811e^-3^ | 0.9980 |
| Quercetin | 4.71 | 3.12 | 3.12-399.13 | Y=2.4595e^-3^X+9.0857e^-4^ | 0.9975 |
| Kaempferol | 5.95 | 3.12 | 3.12-398.75 | Y=2.0840e^-3^X+4.5633e^-4^ | 0.9979 |
| Cuscutamine | 4.27 | 6.25 | 6.25-800.04 | Y=3.6089e^-3^X+4.4207e^-3^ | 0.9994 |
| Isorhamnetin | 6.07 | 0.78 | 0.78-99.91 | Y=3.2448e^-2^X+1.4260e^-3^ | 0.9991 |
| Ferulic acid | 3.08 | 6.00 | 6.00-767.6 | Y=7.6232e^-4^X+2.2999e^-4^ | 0.9916 |
| Caffeic acid | 2.46 | 0.60 | 0.60-76.73 | Y=1.0633e^-1^X-5.46503e^-2^ | 0.9973 |
| *p*-Coumaric acid | 2.9 | 6.25 | 6.25-799.68 | Y=6.8816e^-4^X-4.42863e^-3^ | 0.9912 |

**Table S8.** Precision and accuracy of 9 analytes at LLOQ in rat plasma (n=5)

| Analytes | Spiked conc. (ng/mL) | Mean(ng/mL) | RE% | RSD% |
| --- | --- | --- | --- | --- |
| Isorhamnetin-7-β-O-glucoside | 0.08 | 0.07 | -7.77 | 10.89 |
| Hyperoside | 0.39 | 0.37 | -4.27 | 4.93 |
| Quercetin | 3.12 | 3.25 | 4.32 | 5.88 |
| Kaempferol | 3.12 | 3.06 | -1.92 | 10.81 |
| Cuscutamine | 6.25 | 6.06 | -2.98 | 5.34 |
| Isorhamnetin | 0.78 | 0.78 | -0.57 | 6.21 |
| Ferulic acid | 6.00 | 6.38 | 6.36 | 4.49 |
| Caffeic acid | 0.60 | 0.65 | 7.73 | 4.62 |
| *p*-Coumaric acid | 6.25 | 6.51 | 4.25 | 15.53 |

**Table S9.** Recoveries and matrix effects of 9 analytes (n=5) in rat plasma

| Analytes | Spiked conc. (ng/ml) | Matrix effect(n=5) | | Recovery(n=5) | |
| --- | --- | --- | --- | --- | --- |
|  |  | **RE%** | **RSD%** | **Mean%** | **RSD%** |
| Isorhamnetin-7-β-O-glucoside | 0.16 | 87.75 | 3.61 | 109.72 | 4.98 |
|  | 0.63 | 114.62 | 4.05 | 83.32 | 5.76 |
|  | 5.00 | 121.70 | 0.84 | 85.41 | 1.35 |
| Hyperoside | 0.78 | 90.91 | 4.86 | 107.45 | 3.90 |
|  | 3.12 | 116.00 | 2.40 | 94.48 | 2.39 |
|  | 24.99 | 110.84 | 0.79 | 89.58 | 0.78 |
| Quercetin | 6.24 | 118.23 | 10.80 | 90.27 | 3.65 |
|  | 24.95 | 83.05 | 2.04 | 119.20 | 2.07 |
|  | 199.56 | 95.18 | 2.02 | 95.05 | 0.64 |
| Kaempferol | 6.23 | 110.86 | 13.97 | 93.83 | 6.88 |
|  | 24.92 | 113.54 | 3.32 | 91.58 | 3.00 |
|  | 199.38 | 108.26 | 1.33 | 96.24 | 2.04 |
| Cuscutamine | 12.50 | 98.62 | 2.59 | 100.51 | 1.87 |
|  | 50.00 | 103.63 | 3.28 | 103.27 | 2.91 |
|  | 400.02 | 98.61 | 1.84 | 99.55 | 0.83 |
| Isorhamnetin | 1.56 | 111.67 | 5.37 | 97.37 | 1.93 |
|  | 6.24 | 93.69 | 9.09 | 111.29 | 2.54 |
|  | 49.96 | 97.55 | 1.43 | 106.75 | 3.44 |
| Ferulic acid | 11.99 | 102.14 | 13.90 | 124.67 | 6.44 |
|  | 47.98 | 95.69 | 4.15 | 116.72 | 5.31 |
|  | 383.80 | 99.67 | 3.98 | 113.80 | 3.89 |
| Caffeic acid | 1.20 | 88.20 | 13.78 | 105.75 | 3.67 |
|  | 4.80 | 121.70 | 4.93 | 101.49 | 4.08 |
|  | 38.36 | 101.62 | 3.32 | 106.96 | 1.44 |
| *p*-Coumaric acid | 12.50 | 119.78 | 12.78 | 113.30 | 7.81 |
|  | 49.98 | 89.34 | 7.65 | 118.36 | 6.60 |
|  | 399.84 | 98.65 | 1.78 | 111.93 | 1.84 |

**Table S10.** Intra-day and inter-day precision and accuracy of 9 analytes at three concentration (LQC, MQC, HQC) in rat plasma

| Analytes | Inter-day(n=5) | | | | Intra-day(n=15) | | |
| --- | --- | --- | --- | --- | --- | --- | --- |
|  | Spiked conc. (ng/ml) | Obeserved conc. (ng/ml) | Accuracy RE% | Precision RSD% | Obeserved conc. (ng/ml) | Accuracy RE% | Precision RSD% |
| Isorhamnetin-7-β-O-glucoside | 0.16 | 0.16 | 0.39 | 8.24 | 0.15 | -0.95 | 8.59 |
|  | 0.63 | 0.61 | -1.72 | 1.66 | 1.63 | 1.38 | 4.08 |
|  | 5.00 | 5.07 | 1.32 | 2.45 | 5.00 | 0.00 | 2.97 |
| Hyperoside | 0.78 | 0.78 | 0.14 | 5.32 | 0.78 | -0.07 | 3.49 |
|  | 3.12 | 3.10 | -0.60 | 2.76 | 2.99 | -4.18 | 3.66 |
|  | 24.99 | 25.11 | 0.46 | 2.22 | 24.73 | -1.02 | 2.43 |
| Quercetin | 6.24 | 6.21 | -0.45 | 6.41 | 6.21 | -0.47 | 7.61 |
|  | 24.95 | 25.45 | 2.01 | 1.98 | 25.16 | -0.88 | 3.86 |
|  | 199.56 | 196.46 | -1.55 | 2.31 | 198.63 | -0.47 | 2.78 |
| Kaempferol | 6.23 | 6.20 | -0.43 | 8.32 | 6.16 | -1.07 | 8.57 |
|  | 24.92 | 25.39 | 1.89 | 4.75 | 25.06 | 0.54 | 4.66 |
|  | 199.38 | 196.45 | -1.47 | 2.09 | 198.73 | -0.32 | 3.04 |
| Cuscutamine | 12.50 | 12.47 | -0.27 | 3.47 | 12.52 | 0.12 | 2.80 |
|  | 50.00 | 50.60 | 1.19 | 3.55 | 50.66 | 1.31 | 2.72 |
|  | 400.02 | 396.39 | -0.91 | 2.56 | 397.74 | -0.57 | 1.68 |
| Isorhamnetin | 1.56 | 1.55 | -0.49 | 8.73 | 1.56 | 0.12 | 6.85 |
|  | 6.24 | 6.38 | 2.17 | 3.91 | 6.32 | 1.13 | 3.53 |
|  | 49.96 | 49.12 | -1.68 | 2.94 | 49.67 | -0.58 | 2.88 |
| Ferulic acid | 11.99 | 11.89 | -0.90 | 10.05 | 12.06 | 0.55 | 8.40 |
|  | 47.98 | 48.69 | 1.49 | 8.16 | 48.58 | 1.27 | 8.69 |
|  | 383.80 | 379.38 | -1.15 | 4.49 | 376.67 | -1.86 | 3.55 |
| Caffeic acid | 1.20 | 1.20 | 0.49 | 5.94 | 1.20 | 0.25 | 4.38 |
|  | 4.80 | 4.69 | -2.18 | 1.89 | 4.64 | -3.17 | 2.85 |
|  | 38.36 | 39.01 | 1.69 | 2.83 | 39.33 | 2.52 | 2.44 |
| *p*-Coumaric acid | 12.50 | 12.56 | 0.56 | 9.01 | 12.59 | 0.74 | 9.72 |
|  | 49.98 | 48.75 | -2.46 | 7.12 | 50.49 | 1.02 | 7.43 |
|  | 399.84 | 407.47 | 1.91 | 5.17 | 395.31 | -1.13 | 4.72 |

**Table S11. S**hort-term stability, freeze-thaw stability and auto-sampler stability of 9 analytes in rat plasma (n=5)

| Analytes |  | Short-term stability 25°C for 4 h | | | Freeze-thaw stability Three freeze-thaw cycles | | | Auto-sampler stability 15°C for 24h | | |
| --- | --- | --- | --- | --- | --- | --- | --- | --- | --- | --- |
|  | Spiked Conc. (ng/mL) | Observed Conc. (ng/mL) | Accuracy RE% | Precision RE% | Observed Conc.  (ng/mL) | Accuracy RE% | Precision RE% | Observed Conc. (ng/mL) | Accuracy RE% | Precision RE% |
| Isorhamnetin-7-β-O-glucoside | 0.16 | 0.16 | -0.47 | 3.18 | 0.16 | -0.19 | 3.23 | 0.16 | 0.47 | 4.68 |
|  | 0.63 | 0.64 | 2.07 | 2.17 | 0.63 | 0.78 | 6.20 | 0.61 | -2.08 | 10.37 |
|  | 5.00 | 4.92 | -1.61 | 3.01 | 4.97 | -0.62 | 3.58 | 5.08 | 1.60 | 1.52 |
| Hyperoside | 0.78 | 0.78 | -0.38 | 7.54 | 0.78 | -0.53 | 13.16 | 0.78 | 0.01 | 4.30 |
|  | 3.12 | 3.18 | 1.70 | 2.89 | 3.20 | 2.36 | 3.70 | 3.12 | -0.05 | 4.53 |
|  | 24.99 | 24.66 | -1.32 | 1.78 | 24.53 | -1.82 | 1.75 | 25.00 | 0.04 | 5.24 |
| Quercetin | 6.24 | 6.25 | 0.23 | 8.58 | 6.27 | 0.59 | 8.67 | 6.19 | -0.71 | 6.44 |
|  | 24.95 | 24.69 | -1.01 | 2.37 | 24.29 | -2.63 | 5.01 | 25.73 | 3.13 | 5.52 |
|  | 199.56 | 201.13 | 0.78 | 1.65 | 203.63 | 2.04 | 1.92 | 194.73 | -2.42 | 5.10 |
| Kaempferol | 6.23 | 6.22 | -0.17 | 3.30 | 6.26 | 0.45 | 7.91 | 6.18 | -0.82 | 6.57 |
|  | 24.92 | 25.11 | 0.76 | 3.53 | 24.43 | -1.97 | 3.91 | 25.82 | 3.61 | 5.16 |
|  | 199.38 | 198.21 | -0.59 | 2.43 | 202.42 | 1.53 | 2.17 | 193.80 | -2.80 | 6.30 |
| Cuscutamine | 12.50 | 12.48 | -0.19 | 4.02 | 14.48 | -0.17 | 3.32 | 12.46 | -0.34 | 1.97 |
|  | 50.00 | 50.42 | 0.84 | 1.40 | 50.39 | 0.77 | 3.15 | 50.76 | 1.52 | 1.98 |
|  | 400.02 | 397.43 | -0.65 | 3.01 | 397.63 | -0.60 | 1.97 | 395.31 | -1.18 | 1.54 |
| Isorhamnetin | 1.56 | 1.55 | -0.55 | 4.98 | 1.57 | 0.49 | 4.78 | 1.54 | -1.27 | 3.54 |
|  | 6.24 | 6.40 | 2.44 | 3.60 | 6.11 | -2.16 | 4.91 | 6.05 | -3.07 | 1.34 |
|  | 49.96 | 49.01 | -1.89 | 2.11 | 50.79 | 1.67 | 1.09 | 50.63 | 1.35 | 1.77 |
| Ferulic acid | 11.99 | 12.02 | 0.25 | 6.29 | 11.88 | -0.90 | 8.36 | 1.20 | -0.04 | 14.51 |
|  | 47.98 | 47.45 | -1.10 | 3.08 | 49.89 | 4.00 | 3.27 | 48.45 | 0.99 | 12.07 |
|  | 383.80 | 387.07 | 0.85 | 4.69 | 371.92 | -3.10 | 4.24 | 386.44 | 0.69 | 3.29 |
| Caffeic acid | 1.20 | 1.20 | 0.29 | 1.92 | 1.21 | 0.81 | 2.19 | 1.20 | -0.04 | 3.48 |
|  | 4.80 | 4.73 | -1.30 | 5.42 | 4.62 | -3.60 | 5.95 | 4.80 | 0.20 | 3.55 |
|  | 38.36 | 38.75 | 1.00 | 1.09 | 39.43 | 2.79 | 4.77 | 38.30 | -0.15 | 2.30 |
| *p*-Coumaric acid | 12.50 | 12.40 | -0.79 | 6.92 | 12.41 | -0.65 | 3.45 | 12.41 | -0.71 | 14.37 |
|  | 49.98 | 51.74 | 3.51 | 3.77 | 51.42 | 2.89 | 2.09 | 51.55 | 3.14 | 14.18 |
|  | 399.84 | 388.96 | -2.72 | 4.91 | 390.89 | -2.24 | 1.78 | 390.13 | -2.43 | 4.86 |

**Table S12.** The regression equation (n = 3), linear range and LLOQ (n = 5) of progesterone in cell culture medium

| Analyte | t_R_(min) | Range  (ng/ml) | Linear regression equation (n=3) | Correlation cofficient(r) | LLOQ  (ng/ml) | Mean (ng/mL) | RE% | RSD% |
| --- | --- | --- | --- | --- | --- | --- | --- | --- |
| Progesterone | 4.80 | 1-128 | y=1.0473e^-3^x+5.6314e^-3^ | 0.9969 | 1.00 | 0.96 | -3.8 | 7.5 |

**Table S13.** Recoveries and matrix effects of progesterone (n = 5) in cell culture medium

| **Analyte** | **Spiked conc. (ng/mL)** | **Matrix effect(n = 5)** | | **Recovery(n = 5)** | |
| --- | --- | --- | --- | --- | --- |
|  |  | **Mean(%)** | **RSD(%)** | **Mean(%)** | **RSD(%)** |
| **Progesterone** | 2.0 | 104.6 | 1.7 | 100.8 | 2.4 |
|  | 16.0 | 105.5 | 1.6 | 96.4 | 1.4 |
|  | 64.0 | 104.6 | 0.4 | 96.3 | 1.3 |

**Table S14.** Intra-day and inter-day precision and accuracy of progesterone at three concentration (LQC, MQC, HQC) in cell culture medium

| **Analyte** | **Spiked conc.(ng/mL)** | **Inter-day (n = 5)** | | | **Intra-day (n = 15)** | | |
| --- | --- | --- | --- | --- | --- | --- | --- |
|  |  | **Observed conc.(ng/mL)** | **Accuracy** | **Precision** | **Observed conc.(ng/mL)** | **Accuracy** | **Precision** |
|  |  |  | **RE(%)** | **RSD(%)** |  | **RE(%)** | **RSD(%)** |
| **Progesterone** | 2.0 | 2.0 | -0.3 | 5.0 | 2.0 | -0.2 | 4.7 |
|  | 16.0 | 16.7 | 4.4 | 1.7 | 16.6 | 3.8 | 3.9 |
|  | 64.0 | 61.4 | -4.1 | 2.7 | 61.7 | -3.6 | 2.4 |

**Table S15. S**hort-term stability, long-term stability, freeze-thaw stability and auto-sampler stability of progesterone in cell culture medium (n=5)

| **Analytes** | **Spiked conc.**  **(ng/mL)** | **Short-term stability** | | | **Long-term stability** | | | **Freeze-thaw stability** | | | **Auto-sampler stability** | | |
| --- | --- | --- | --- | --- | --- | --- | --- | --- | --- | --- | --- | --- | --- |
|  |  | **25℃ for 4 h** | | | **-80℃ for 2 month** | | | **Three freeze-thaw cycles** | | | **15℃ for 24 h** | | |
|  |  | **Observed conc.** | **Accuracy** | **Precision** | **Observed conc.** | **Accuracy** | **Precision** | **Observed conc.** | **Accuracy** | **Precision** | **Observed conc.** | **Accuracy** | **Precision** |
|  |  | **(ng/mL)** | **RE(%)** | **RSD (%)** | **(ng/mL)** | **RE(%)** | **RSD (%)** | **(ng/mL)** | **RE(%)** | **RSD (%)** | **(ng/mL)** | **RE(%)** | **RSD (%)** |
| **Progesterone** | 2.0 | 2.0 | -0.2 | 3.1 | 2.0 | -0.3 | 0.8 | 2.0 | -0.3 | 6.2 | 2.0 | -0.2 | 5.7 |
|  | 16.0 | 16.6 | 3.5 | 5.0 | 16.9 | 5.4 | 2.1 | 16.8 | 4.9 | 2.2 | 16.6 | 3.6 | 4.2 |
|  | 64.0 | 61.9 | -3.3 | 2.2 | 60.7 | -5.1 | 1.7 | 61.1 | -4.6 | 1.0 | 61.8 | -3.4 | 2.2 |
